# The Black Fungus Gnats (Diptera, Sciaridae) of Norway – Part I: species records published until December 2019, with an updated checklist

**DOI:** 10.3897/zookeys.957.46528

**Published:** 2020-08-10

**Authors:** Frank Menzel, Øivind Gammelmo, Kjell Magne Olsen, Arne Köhler

**Affiliations:** 1 Senckenberg Deutsches Entomologisches Institut, Eberswalder Straße 90, 15374 Müncheberg, Germany Senckenberg Deutsches Entomologisches Institut Müncheberg Germany; 2 BioFokus, Gaustadalléen 21, 0349 Oslo, Norway BioFokus Oslo Norway

**Keywords:** armyworm, diversity, Europe, faunistics, habitats, literature review, localities, phenology, Scandinavia, Sciaroidea

## Abstract

Black Fungus Gnats (Sciaridae) are a megadiverse, cosmopoliltan family of bibionomorph Diptera. Even in Europe, the continent with the longest tradition in sciarid taxonomy, numerous taxonomic issues remain unresolved and countless species await discovery and description. The fauna of Norway is in these respects no exception. Recognising considerable knowledge gaps, the Norwegian Biodiversity Information Centre provided substantial funding for a detailed inventory of the Sciaridae species occurring in Norway, which was realised in 2014–2018. The results of this project will be published in a series of papers, of which the first is presented here, summarising available data on the taxonomy, faunistics, and autecology of Norwegian Sciaridae beginning with Zetterstedt’s pioneering work in 1838 and ending with 31 December 2019 as the cut-off date. All published records from that period were analysed. The result is a list of 143 species and four unplaced names. Following a consistent scheme, verified locality details are provide including alternative spellings, habitats, and flight times of adults in Norway, literature citations for the faunistic records, and general taxonomic references for classification or identification. A checklist of the sciarid fauna of Norway and a complete list of the relevant literature are also presented.

## Introduction

The Sciaridae is one of the largest families of Diptera, rich in both species and individuals, and plays a significant role in natural ecosystems (summarised in Menzel and Mohrig 2000; Menzel and Schulz 2007). For example, the larvae are important for the litter decomposition in forests (Hövemeyer 1989; Deleporte and Rouland 1991; Deleporte and Charrier 1996), and the adults for the transmission of basidiospores of fungi (Schmidt 1979) and the pollination of plants (Vogel and Martens 2000; Rulik et al. 2008). Sciarids are also well-known as pests in mushroom farms and greenhouses, or as common inhabitants of pot plants in houses (e.g., Broadley et al. 2018).

Often sciarids are one of the most dominant Diptera families in ecological studies (e.g., Thiede 1977; Feldmann 1992; Hövemeyer 1992; Bickel and Tasker 2004), and thousands of specimens can be collected in a short time (Menzel and Schulz 2007). Many species prefer moist, shady deciduous and coniferous woods with a high proportion of dead wood (Hövemeyer 1998, 1999, 2002; Menzel and Schulz 2007). Other species can be found in wetlands (e.g., moist meadows, fens) or xerothermic habitats (e.g., dry grassland, heath) (Hövemeyer 1996; Heller 1998, 2000; Menzel et al. 2006).

**Figures 1–3.**
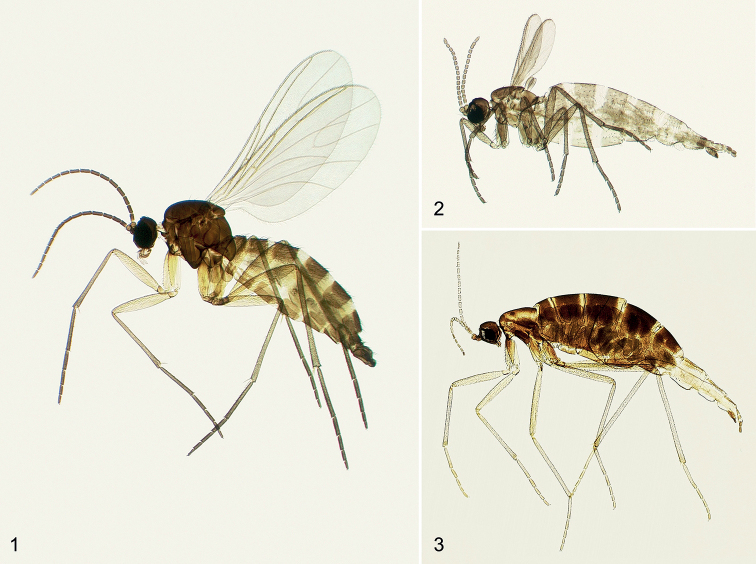
Habitus of Norwegian Sciaridae**1** winged male of *Bradysia fenestralis* (Zetterstedt, 1838) **2** brachypterous female of *Corynoptera minima* (Meigen, 1818) **3** apterous female of *Epidapus gracilis* (Walker, 1848).

The Black Fungus Gnats (Figs 1–3) are inconspicuous, minute to medium-sized flies (0.8–7.0 mm body length) and are fairly uniform in appearance. While adults of most species are completely black or dark brown, others exhibit some yellow or orange. The head is relatively small and usually rounded, with the eyes meeting at a narrow bridge above the antennae. There are three ocelli on the forehead. The antennae are long and thin, with 16 segments. Of the mouthparts, which are generally inconspicuous, only the palpi are of relevance for taxonomy. The body is almost hairless at first glance. The wings are rather broad and rounded at the apex, often smoky-coloured, with a distinctively curved vein fork (M_1_+M_2_) in the middle of the apex of wing. Females of some species have reduced wings (e.g., in *Epidapus* Haliday). The legs are long and slender, but not as long as for example in Mycetophilidae. The larvae are cylindrical, white and shiny, with a clearly sclerotised, dark head capsule. Detailed descriptions for the preimaginal stages and adults, and their importance for the identification and classification of sciarids, are given by Menzel and Mohrig (2000) and Menzel and Smith (2017).

Compared to most other Diptera in Norway, Sciaridae have previously attracted little attention from entomologists. Notorious for their uniformity and small body size, adult sciarids are largely the domain of taxonomic specialists, while larvae of most species remain undiscovered. The earliest mention of Black Fungus Gnats in Norway was by Ramus (1735), who reported about the ‘armyworm’, a migration of thousands of sciarid larvae. However, the first taxonomic studies of Sciaridae in Norway are those of Zetterstedt (1838–1860), Walker (1848), Siebke (1853–1877), and Holmgren (1869). Later, Edwards (1923–1935), Lengersdorf (1926b–1930c), Soot-Ryen (1942), Frey (1948), and Tuomikoski (1960, 1967) contributed to the knowledge of the sciarid fauna of Norway. Some of these early records, but not all, were later treated in a modern review of the family (Menzel and Mohrig 2000). Relatively few recent studies exist, but notably Thunes et al. (2004), Hippa et al. (2010), Köhler et al. (2014), and Heller et al. (2016) have presented new and valuable information on the fauna of Norwegian Sciaridae.

In 2014 the Norwegian Biodiversity Information Centre (NBIC) granted the project ‘Sørgemygg i Norge’ (Sciaridae of Norway) funding for the period 2014–2016. Later NBIC also granted funding for the project ‘Sørgemygg i norske skoger’ (Sciaridae in Norwegian forests), which is effectively the second phase of our research work. This ran from January 2017 to December 2018. Our study collates the records published between 1735 and 2019 and provides many corrected locality data for the Norwegian sciarid fauna. The revised nomenclature and the evaluation of faunistic records at species level form the basis for an updated checklist. For the first time, information is also summarised on the identified habitats and the phenology of species in Norway. Consequently, all results presented here comprise the published ‘status quo’, form the basis for the evaluation of our faunistic work in both mentioned NTI projects, and are the starting point for a series of papers on the Norwegian fauna. Many unpublished data on the Black Fungus Gnats of Norway, based on the identification of specimens in several museum collections, or on the samples collected by the authors between 2014 and 2018, shall be published in this series.

## Material and methods

**Figure 4.**
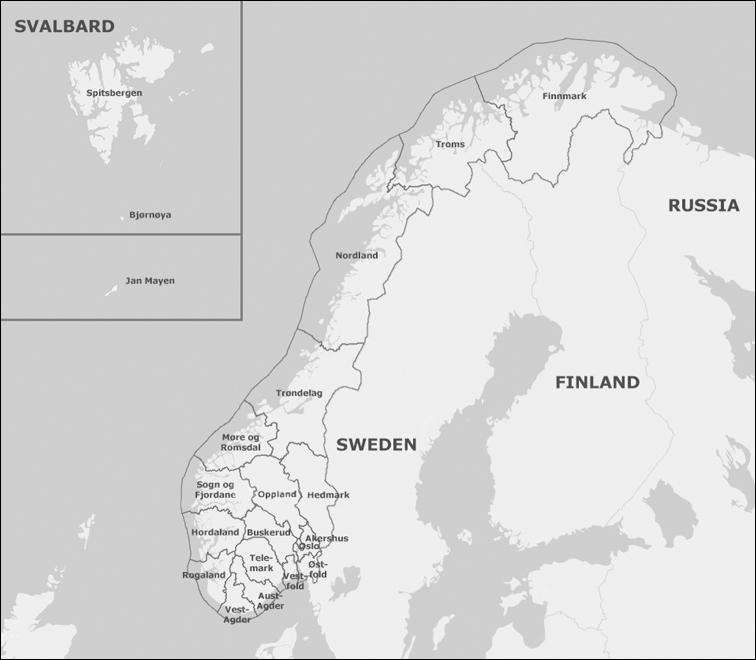
The studied area for the sciarid fauna of Norway, subdivided into 18 mainland counties, the island Jan Mayen and the Svalbard archipelago comprising Bjørnøya and Spitsbergen.

Norway, Europe’s sixth largest country by land area, occupies approximately half of the Scandinavian Peninsula, bordering Sweden to the east, and Finland and Russia to the northeast (Fig. 4). The Norwegian mainland extends from 57.9 to 71.2N. The extensive coastline is dominated by many fjords and numerous islands, making it highly indented and irregular. The remote island of Jan Mayen (70.5–71.1N, 07.6–09.0E) and the archipelago of Svalbard comprising Bjørnøya (74.2–74.3N, 18.4–19.1E) and Spitsbergen (76.3–80.5N, 10.3–33.3E) are also parts of the Kingdom of Norway.

All data analysed here were taken from both the scientific literature and publications in the media. They relate exclusively to the sciarid specimens recorded from Norway. The great total amount of data made it impossible for us to validate all the species identifications on which published records are based. To enable comparison with previous faunal lists, references to earlier records were added to the list and the synonymous names were given for each species.

### Nomenclature and systematics

Employed nomenclature and systematics are mainly based on the revision of Palaearctic fauna (Menzel and Mohrig 2000), the revision of Nearctic fauna (Mohrig et al. 2013), and some works after 2000. These comprise Hippa and Vilkamaa (2004, 2016) [*Xylosciara*, *Claustropyga*]; Hippa et al. (2003, 2010) [*Claustropyga*, *Corynoptera* s. str.]; Vilkamaa and Menzel (2019) [*Lycoriella*, *Hemineurina*, *Trichocoelina*] and Vilkamaa et al. (2004, 2013a) [*Dichopygina*, *Camptochaeta*]. The proposal by Mohrig et al. (2017), who postulated *Ctenosciara* Tuomikoski, 1960 as a junior synonym of *Austrosciara* Schmitz & Mjöberg, 1924, was not followed here, because the procedure used therein is contrary to the International Code of Zoological Nomenclature (ICZN 1999), and the type specimens of the type species have not yet been revised and compared. Some other nomenclatural problems at the species level are discussed at the appropriate places in ‘Taxonomic notes’.

### Presentation of data

All literature sources containing data and information on the Norwegian sciarid fauna are cited for each species under ‘*Faunistics*’. Various outdated catalogues (e.g., Kertész 1902, 1903; Gerbachevskaja-Pavluchenko 1986) were not evaluated because they do not contain primary data for the sciarid fauna of Norway and/or their abstracted and largely unverifiable content may lead to false results. In addition, in the category ‘*Taxonomy*’ publications are mentioned that are important for the classification, nomenclature and/or identification of the included sciarid species.

**Locality data**. Due to the geographical peculiarities, the Norwegian mainland with the offshore islands is treated first in the faunistic section on each species. Counties (fylke) and localities are listed in alphabetical order, unlike the traditional practice in lists of Norwegian fauna, which are arranged from south to north. Because the Arctic island of Jan Mayen and the Arctic archipelago of Svalbard are very remote from the Norwegian mainland, they are considered separately. Faunistic records for these islands are summarised in a separate block at the end of the locality lists, following the ‘mainland records’. Geographic names are given in both modern Norwegian script and the spelling(s) used in the original literature, to facilitate their location in geographical maps and electronic resources. The reclassification of the Norwegian counties valid since 1 January 2020 was not taken into account here.

If available, all information about a locality is presented in a unified data structure as follows:

“• *County; municipality, region/island(s), city/village/collecting place* without or with an explanation of more precise geographic location (= ‘citation of different *original spellings* in the analysed literature, including wrong spellings’)”. For example, datasets of localities in various publication languages and/or with different spellings would look as follows:

“• Finnmark; Vardø, Varangerhalvøya, Persfjorden (= ‘Finmark, Vardø, Persfjord’; = ‘Vardö, Persfjord’) • Oslo; Oslo, Tøyen (= ‘ad Christianiam in Tøien’; = ‘Tøien ad Christianiam’; = ‘in Tøien ad Christ.’; = ‘Töien nahe Kristiania [Oslo]’; = ‘Tøien’; = ‘Töien’; = ‘Toiën’).

• Jan Mayen; without further locality details (= ‘Jan Mayen’; = ‘Jan Mayen Island’) • Svalbard; Bjørnøya, mining camp Tunheim on the NE coast (= ‘Bear Island, Tunheim’) • Spitsbergen, Isfjorden, Dickson Land, Kapp Thordsen (= ‘in Spetsbergia ad Cap Torsden in Isfjorden’; = ‘Cap Torsden’).”

**Ecological data**. If the literature sources provide information on habiat and/or temporal occurrence of a species, these data were summarised in an ‘Ecological note’. All data published here refer exclusively to the Norwegian sciarid fauna. It should be noted that for most species the habitat requirements are poorly known or missing, so notes in this paragraph should be considered as incomplete. For example, the listing of a single habitat type does not necessarily mean that the species is only adapted to that habitat or that this information applies to all published Norwegian records and/or collected specimens. For many Norwegian species with few published data, no information currently exists on the habitat or the flight time of adults. Such ‘negative results’ of our literature study are indicated in the ecological notes of the species concerned by ‘Habitat not specified’ and/or ‘Phenology: without data’.

### Meanings of common Norwegian locality names

Listed here are Norwegian words, or suffixes, frequently occurring in place names together with their English translations:

-**dal**/-**dalen** = valley; -**bukta**/-**bugten** = bay; -**elv**/-**elva** = river; -**fjell**/-**fjellet**/-**fjella** = mountain(s); -**fjellstue** = mountain lodge/inn; -**fjord**/-**fjorden** = fjord; -**halvøya** = peninsula; -**hytta** = hut/bothy; -**øy**/-**øya** = island; -**stua**/-**stue** = cabin/hostel/mountain lodge; -**vann**/-**vannet**/-**vatn**/-**vatnet** = lake; -**vidda** = plateau.

### Abbreviations

The following general abbreviations are used in the text: **E** = eastern; **N** = northern; **NE** = northeastern; **NW** = northwestern; **S** = southern; **SE** = southeastern; **SW** = southwestern; **W** = western; ? = questionable content (concerning locality names, species identifications, or cited references in literature); **SG** = subgenus (only used in the checklist); **Mar.**–**Dec.** = month (March to December) in relation to the collecting time (= flight time of imagines).

Some museums and institutions were abbreviated as follows: **BFCO** = BioFokus Collection, Oslo, Norway; **NHMO** = University of Oslo, Natural History Museum, Oslo, Norway; **NIBR** = Norwegian Institute of Bioeconomy Research, Ås, Norway; **NTNU-VM** = Norwegian University of Science and Technology, University Museum, Department of Natural History, Trondheim, Norway; **PWMP** = Private Collection of Werner Mohrig, Poseritz, Germany; **SDEI** = Senckenberg Deutsches Entomologisches Institut, Müncheberg, Germany; **TMUC** = The Arctic University Museum of Norway, Tromsø Museum, Tromsø, Norway; **UZMH** = University of Helsinki, Finnish Museum of Natural History, Helsinki, Finland.

## Results

### Records of ‘armyworms’

Some publications, especially older ones, contain reports about spectacular processions of sciarid larvae. The up to ten meters long columns are called an ‘armyworm’ in the popular language. In the studied literature sources which refer to Norway, can also be found – partly in other languages – the synonym names ‘hærorm’ (= ‘Heervurm’; = ‘Heerwurm’), ‘dragfæ’ (=‘dragfæe’; = ‘fedrag’), ‘ormedrag’ (= ‘Orme-Drag’; = ‘Wurmdrache’), ‘budrag’, ‘markskrei’, ‘härmask’, ‘hærmygg’, ‘hærsørgemygg’ or ‘sørgemygg’. In Norway, these have been partly associated with the species *Sciara hemerobioides* (Scopoli, 1763) [= *thomae* (Linnaeus, 1767)] (Berthold 1854: 30; Berthold 1856: 66; Schøyen 1926: C31; Hansen and Granrud 2011: 46) and *Sciara militaris* Nowicki, 1868 (Schøyen 1893: 41; Schøyen 1917: 94; Schøyen 1936: 81; Sundby 1967: 6; Hansen and Granrud 2011: 46). No reliable information currently exists on the occurrence of the ‘armyworm creator’ *Sciara militaris* Nowicki, 1868 in Norway. Although adults of *Sciara hemerobioides* have been found several times in Norway, none of the specimens mentioned in the evaluated literature sources was reared from larvae of an ‘armyworm’. Besides the two *Sciara* species discussed above, other sciarids can also form long columns of larvae. The same phenomenon has already been reported from other European countries in the species *Bradysia bicolor* (Meigen, 1818), *Cratyna perplexa* (Winnertz, 1867), *Ctenosciara hyalipennis* (Meigen, 1804) and *Sciara analis* Schiner, 1864 (Menzel and Mohrig 2000: 17). Except for *Sciara analis* these are species that are also very common in Norway. For these reasons, all unspecified ‘armyworm’ records are not assigned to any Norwegian sciarid species and are listed in the section ‘Doubtful species’.

The Norwegian records of ‘armyworms’ were presented or summarised in the following publications: Ramus (1735) [1715]: 240; Berthold (1845): 65, 66; Boheman (1847): 22; Berthold (1854): 4, 5; Berthold (1856): 40, 41; Schøyen (1893): 41; Schøyen (1917): 94; Schøyen (1926): C31; Schøyen (1936): 81; Sundby (1967): 4; Greve Jensen (1979): [unpaginated page]; Menzel and Mohrig (2000): 18; Hansen and Granrud (2011): 45; Stenløkk (2011): 9 [without data]; Giske and Blåsmo Aronsen (2016): [unpaginated page]; Nesvold (2017a): [unpaginated page]; Nesvold (2017b): [unpaginated page]; Ringsaker (2018): [unpaginated page].

Finds of ‘armyworms’ have been reported from the following Norwegian localities: • Norway; without further locality details (= ‘i Norrige’; = ‘in Norwegen’) – Ramus (1735) [1715]: 240; Pontoppidian (1753): 67; Pontoppidian (1755b): 42; Berthold (1845): 65, 66; Boheman (1847): 22; Berthold (1854): 4, 5; Berthold (1856): 40, 41 • Akershus; Aurskog-Høland, N part of the area Aurskog [formerly ‘Urskog’] NW of Aursmoen and SE of Blaker (= ‘ved Blaker i Urskog’) – Schøyen (1917): 94; Sundby (1967): 5 • Buskerud; Modum [without exact locality] – Hansen and Granrud (2011): 48 [based on a forum blog by Haavik (2009)] • Hedmark; Eidskog [in the former district ‘Hedmarka’] (= ‘Eidsskogen, Hedemarken’; = ‘i Eidsskogen på Hedemarken’) – Schøyen (1917): 94; Sundby (1967): 5 • Løten, in Løten (= ‘i Løyten’) – Schøyen (1893): 41; Sundby (1967): 5 • Hordaland; Bergen (= ‘Bergen’) – Menzel and Mohrig (2000): 18 • Bergen, Søreidgrenda, Søviknes [today settlement area at the street Søvikneset] (= ‘på Søviknes i Fana’) – Greve Jensen (1979): [unpaginated page] • Møre Og Romsdal; Eide, Vevang SW of Kristiansund (= ‘ved Vevang pr. Kristiansund’; = ‘på Vevang ved Kristiansund’) – Schøyen (1926): C31; Sundby (1967): 5 • Nordland; Sørfold, Seljåsen (= ‘Seljåsen, Nordland’) – Ringsaker (2018): [unpaginated page] • Oppland; Sel, Heidal, side valley Heidal in the Gudbrandsdalen (‘Heidal i Sel kommune i Gudbrandsdalen’) – Hansen and Granrud (2011): 46 • Østfold; Eidsberg, Mysen (= ‘Mysen i Østfold’) – Schøyen (1936): 81 • Rømskog, Flaten near the Swedish border (= ‘på Flaten i Rømskog’; = ‘Flaten i Rømskog’) – Sundby (1967): 4; Hansen and Granrud (2011): 48 • Troms; Tromsø, village Tromvika on the Kvaløya (= ‘Tromvika i Tromsø’) – Giske and Blåsmo Aronsen (2016): [unpaginated page] • Trøndelag; Holtålen, Ålen, on the Hessjøvegen NW of the lake Hessjøen (= ‘ved Hessjøen i Holtålen, på Hessjøvegen’) – Nesvold (2017a): [unpaginated page]; Nesvold (2017b): [unpaginated page] • Vest-Agder; Flekkefjord, Gyland NE of Flekkefjord (= ‘Gyland ved Flekkefjord’) – Schøyen (1936): 81.

Faunistic note: Most of the Norwegian armyworm records in Menzel and Mohrig (2000: 18), taken from historical literature of the 18^th^ Century (Ramus 1735; Pontoppidan 1753, 1755b), were incorrectly translated or indicate only unspecified localities. So, the expression ‘Baand in Vejen’ (recte ‘og Baand i Vejen’)], originates from Ramus (1735: 240), lines 15–17: ‘*Gemeene Folk*, …, *kaste deres Klæder****og Baand i Vejen****for dem*, …’, which in Danish language simply means ‘*Common people ... throw their clothes and ribbons in their way* …’. The title ‘*Firefodde Dyr*, *som findes****i Norrige****samt krybende Orme*’ [= ‘*Four-footed animals, found****in Norway****as well as creeping worms*’] indicates, that Ramus (1735: 240) mentions the armyworm (‘dragfæ’ or ‘ormedrag’) for the first time for Norway, which was cited by Pontoppidan (1753: 68; 1755b: 42).

Those armyworm records included in Menzel and Mohrig (2000: 18) refer to the preceding definition of the area in the first part of the ‘Natural history of Norway’ by Pontoppidan (1752: 67; 1755a: 41) and are not certain. Accordingly, the following misinterpreted localities are to be deleted from the list of Norwegian armyworm records (cited from the secondary literature): ‘Bygle-Field = Byglefield; Dofre-Field = Dofrefield; File-Field = Filefield; Hardanger-Field = Hardangerfield; Halne-Field = Halnefield; Hekle-Field = Hecklefield [also as ‘Seklefield’]; Jokle-Field = Joklefield; Lang-Field = Langfield; Loms-Field = Lomsfield; Norden-Fields = Nordenfield; Sogne-Field = Sognefield; Sønden-Fields = Søndenfield; Tronhiems Stift; Vesten-Fields = Vestenfield’.

### Alphabetical list of Norwegian species with associated data

#### Bradysia affinis

Taxon classificationAnimaliaDipteraSciaridae

(Zetterstedt, 1838)

77B8EB74-857A-5954-9AC9-00597F0FC537

##### Synonym.

= *pratincola* Tuomikoski, 1960.

##### Literature.

*Faunistics*: Zetterstedt (1851): 3752; Siebke (1877): 214 [both as *Sciara affinis*]; Tuomikoski (1960): 119 [only correctly mentioned under *Bradysia affinis* as ‘in Norwegen’ [in Norway] based on the old records by Zetterstedt (1851) and Siebke (1877); the rest are misidentifications] and 120 [as *Bradysia pratincola*]; Menzel et al. (1990): 368 [as *Bradysia pratincola* only; not *Bradysia affinis* sensu Tuomikoski; misidentification]; Menzel and Mohrig (2000): 178 [as *Bradysia pratincola*]; Köhler et al. (2014): 328 [as *Bradysia affinis*]. *Taxonomy*: Tuomikoski (1960): 116, 120 [as *Bradysia pratincola*]; Menzel and Mohrig (1998): 356 [as *Bradysia affinis*]; Menzel and Mohrig (2000): 172 [as *Bradysia affinis*] and 178 [as *Bradysia pratincola*].

##### Localities.

• Norway; without further locality details (= ‘Norwegen’; = ‘Norway’) • Finnmark; Tana, Tanafjorden, fjord Vestertana (= ‘Finmark, Tana, Vestertana’) • Telemark; Drangedal, Djupedal 1.5 km SE of Henneseid (= ‘Drangedal, Djupedal, Henseid’) • Drangedal, woodland Steinknapp SW of Drangedal (= ‘Drangedal, Steinknapp’) • Troms; Nordreisa, woodland and farm Hallen at the E shore of Reisaelva SE of Storslett (= ‘Nordreisa, Hallen’) • Tromsø (= ‘Tromsø’; = ‘Tromsö’) • Trøndelag; Verdal, Østre Nes at the Jamtlandsvegen [road no. 72] between Verdal and Lysthaugen (= ‘ad diversorium Näs Værdaliæ’; = ‘ad diversorium Næs Værdaliæ’).

##### Ecological note.

Grove meadows. Phenology: Jun.–Aug.

#### Bradysia alpicola

Taxon classificationAnimaliaDipteraSciaridae

(Winnertz, 1867)

A9DC4B50-9682-5C7A-9236-99EDA4C12B4A

##### Synonyms.

= caliginosa (Winnertz, 1867); = concolor (Beling, 1873); = egens (Winnertz, 1867); = meridiana (Lengersdorf, 1926); = moreensis (Lengersdorf, 1926); = *mutabilis* (Lengersdorf, 1926); = obscura (Winnertz, 1867); = rogenhoferi (Winnertz, 1867).

##### Literature.

*Faunistics*: Lengersdorf (1926b): 7 [as *Sciara mutabilis*]; Lengersdorf (1928–30): 6, 31 [as Lycoria (Neosciara) mutabilis]; Soot-Ryen (1942): 78 [as *Neosciara mutabilis*]; Menzel and Mohrig (2000): 164 [as *Sciara mutabilis* under *Bradysia alpicola*]; Köhler et al. (2014): 328 [as *Bradysia alpicola*]. *Taxonomy*: Zetterstedt (1851): 3716 [as *Sciara morio*; misidentification]; Tuomikoski (1960): 125 [as *Bradysia morio* sensu Zetterstedt and Frey; misidentification]; Menzel and Mohrig (2000): 163 [as *Bradysia alpicola*].

##### Localities.

• Norway; without further locality details (= ‘Norwegen’) • Finnmark; Alta, Bojobæskihytta in the Stabbursdalen between Karasjok and Alta (= ‘Bojobæske’; = ‘Bojobaeske’) • Alta, Jotkajavre fjellstue on the Finnmarksvidda between Karasjok and Alta (= ‘Jotkajavre’) • Karasjok, Karasjok at the river Karasjohka (= ‘Karasjok’) • Nordland; Sørfold, Røsvik at the S shore of Sørfolda (= ‘Røsvik’; = ‘Røsvik [? Rørvik]’) • Telemark; Drangedal, woodland Steinknapp SW of Drangedal (= ‘Drangedal, Steinknapp’).

##### Ecological note.

Oak canopies of *Quercus robur*. Phenology: Jun.–Aug.

#### Bradysia angustipennis

Taxon classificationAnimaliaDipteraSciaridae

Winnertz, 1867

E2B0B59C-FA9F-54E5-B054-7B10737B4A9E

##### Synonyms.

= campestris Mohrig & Mamaev, 1970; = pedestris (Kieffer, 1903).

##### Literature.

*Faunistics*: Vilkamaa and Hippa (2004): 21 [as *Bradysia angustipennis*]. *Taxonomy*: Menzel and Mohrig (2000): 119 [as *Bradysia angustipennis*].

##### Locality.

• Norway; without further locality details (= ‘Norway’).

##### Faunistic note.

The single Norwegian record of *Bradysia angustipennis* was published by Vilkamaa and Hippa (2004) in a phylogenetic analysis (appendix 2) as ‘Norway’ without further locality details. The male specimen is deposited in the UZMH collection and was not revised here.

##### Ecological note.

Habitat not specified. Phenology: without data.

#### Bradysia bicolor

Taxon classificationAnimaliaDipteraSciaridae

(Meigen, 1818)

A2CA2E20-B4B9-5F83-BB38-54C488DC3E3F

##### Synonyms.

= abdominalis (Lehmann, 1824); = *bicolor*var. alpestris (Lengersdorf, 1926); = *bore* (Walker, 1848); = *rufiventris* (Macquart, 1834).

##### Literature.

*Faunistics*: Walker (1848): 107 [as *Sciara bore*]; Zetterstedt (1851): 3724 [as *Sciara bicolor*] and 3725 [as *Sciara rufiventris*]; Siebke (1853): 305; Zetterstedt (1855): 4889 [both as *Sciara rufiventris*]; Siebke (1866b): 417; Siebke (1877): 211 [both as *Sciara bicolor* and *Sciara rufiventris*]; Becher (1886): 62; Strand (1904): 9; Edwards (1923): 236 [all as *Sciara bicolor*]; Lengersdorf (1926b): 3 [as *Sciara bicolor*] and 9 [as *Sciara rufiventris* and *Sciara bore*]; Soot-Ryen (1942): 77 [as *Neosciara bicolor*]; Mohrig and Menzel (1993): 270 [as *Bradysia bicolor*]; Menzel and Mohrig (2000): 133 [as *Bradysia bicolor* and *Sciara bore* under *Bradysia bicolor*]. Coulson and Refseth (2004): 102 [as *Bradysia bicolor*]. *Taxonomy*: Tuomikoski (1960): 137, 139; Mohrig and Menzel (1993): 270; Menzel and Mohrig (2000): 133 [all as *Bradysia bicolor*].

##### Localities.

• Norway; without further locality details (= ‘Nord-Norwegen’) • Buskerud; Røyken (= ‘in par. [parochia] Røken’; = ‘Røken’; = ‘Røyken’) • Finnmark; Alta, Jotkajavre fjellstue on the Finnmarksvidda between Karasjok and Alta (= ‘Jotkajavre’) • Hammerfest, Hammerfest (= ‘Hammerfest’; = ‘Hammerfest, Finmark’; = ‘Hammerfest, Finmark [Hammerfest auf der Insel Kvalöya]’) • Nordland; Hattfjelldal, Røssvatnet (= ‘Røsvand’; = ‘Røssvatn’) • Nord-Helgeland, Ranfjorden (= ‘Ranfjord’) • Oppland; Nord-Fron or Sør-Fron in the Gudbrandsdalen (= ‘Gudbrandsdalen, Fron’) • Øyer in the Gudbrandsdalen (= ‘Øier Gudbrandsdaliæ’; = ‘Gudbrandsdalen, Öier’; = ‘Öier’) • Øyer, Moshus in the SE part of Øyer in the Gudbrandsdalen (= ‘Øier Gudbrandsdaliæ ad Moshus’; = ‘Moshus, Øyer’) • Troms; Tromsø, Ramfjorden (= ‘Ramfjord’) • Trøndelag; Levanger, Alstadhaug (= ‘Alstadhaug, Levanger’; = ‘ad Alstadhaug’; = ‘ad Alstahaug’) • Levanger, Levanger (= ‘ad Levanger’; = ‘Levanger’) • Vestfold; Sandefjord (= ‘ad Sandefjord’; = ‘ved Sandefjord’; = ‘Sandefjord’) • Stavern (= ‘ad oppidum Staværn’; = ‘ved Staværn’; = ‘Staværn’).

• Jan Mayen; without further locality details (= ‘Jan Mayen’; = ‘Jan Mayen Island’).

##### Ecological note.

Habitats not specified. Phenology: Jul.–Aug.

#### Bradysia brevispina

Taxon classificationAnimaliaDipteraSciaridae

Tuomikoski, 1960

70DFDF4B-A961-5795-A125-59D17938E191

##### Literature.

*Faunistics*: Thunes et al. (2004): 72, 85 [as *Bradysia brevispina*]. *Taxonomy*: Tuomikoski (1960): 130, 135; Menzel and Mohrig (2000): 151 [both as *Bradysia brevispina*].

##### Localities.

• Norway; without further locality details (= ‘Norway’) • Buskerud; Sigdal, Heimseteråsen (= ‘Sigdal’).

##### Ecological note.

*Pinus sylvestris* dominated boreal forests with *Betula pubescens* and *Picea abies*. Phenology: Jul.

#### Bradysia confinis

Taxon classificationAnimaliaDipteraSciaridae

(Winnertz, 1867)

3D888CA0-6095-5AA7-833C-C81201330AE7

##### Synonyms.

= myrtilli (Winnertz, 1867); = nigrescens (Winnertz, 1869); = occulta (Winnertz, 1867); = *sororcula* (Winnertz, 1867); = tarda (Winnertz, 1867).

##### Literature.

*Faunistics*: Lengersdorf (1926b): 3 [as *Sciara sororcula*]; Soot-Ryen (1942): 78 [in part as *Neosciara nervosa*; misidentification (only cited *sororcula* specimens)]. *Taxonomy*: Tuomikoski (1960): 139, 140; Menzel and Mohrig (2000): 127 [both as *Bradysia confinis*].

##### Localities.

• Finnmark; Alta, Bossekop in Alta (= ‘Bosekop’) • Hammerfest, Hammerfest (= ‘Hammerfest’) • Porsanger, farm Fæstningsstua near Lævnasjarvi W of Skoganvarre (= ‘Fæstningstuen’; = ‘Festningsstuen’) • Møre Og Romsdal/Oppland/Trøndelag; Dovrefjell [Dovre Mountains] (= ‘Dovre’) • Troms; Balsfjord, Svendborg ca. 1.7 km from the N shore of Fjellfrøsvatnet (= ‘Svendborg’; = ‘Svendborg’) • Karlsøy, Torsvåg at the NW coast of Vannøya 15 km N of Tromsø (= ‘Torsvaag’; = ‘Torsvåg’) • Tromsø (= ‘Tromsø’) • Tromsø, Ramfjorden (= ‘Ramfjord’).

##### Ecological note.

Habitats not specified. Phenology: Jul.–Aug.

#### Bradysia distincta

Taxon classificationAnimaliaDipteraSciaridae

(Staeger, 1840)

B45ADABE-E3D4-5D3A-9E98-87070CB4902F

##### Synonyms.

= egregia (Beling, 1873); = fastuosa (Winnertz, 1867); = insignis (Winnertz, 1867).

##### Literature.

*Faunistics*: Siebke (1877): 212 [as *Sciara distincta*]; Soot-Ryen (1942): 78 [in part as *Neosciara morio*; misidentification (only cited *distincta* specimen)]. *Taxonomy*: Menzel and Mohrig (2000): 165 [as *Bradysia distincta*].

##### Locality.

• Møre Og Romsdal/Oppland/Trøndelag; Dovrefjell [Dovre Mountains] (= ‘in alpe Dovre’; = ‘Dovre’).

##### Ecological note.

Habitat not specified. Phenology: Jul.

#### Bradysia fenestralis

Taxon classificationAnimaliaDipteraSciaridae

(Zetterstedt, 1838)

1F00617A-BE4C-5B5E-857B-EBC85EA65238

##### Synonyms.

= *bulbostyla* Mohrig & Menzel, 1990; = *frigida* (Winnertz, 1867); = *signata* (Winnertz, 1867).

##### Literature.

*Faunistics*: Köhler et al. (2014): 328 [as *Bradysia fenestralis*]. *Taxonomy*: Menzel and Mohrig (1998): 354 [as *Bradysia fenestralis*]; Menzel and Mohrig (2000): 120 [as *Bradysia bulbostyla*] and 153 [as *Bradysia fenestralis*]; Menzel and Heller (2005): 349 [as *Bradysia fenestralis*]; Menzel and Heller (2006): 49 [as *Bradysia signata*].

##### Locality.

• Telemark; Drangedal, 300 m SE of Henneseid (= ‘Drangedal, Henseid’).

##### Faunistic note.

The first specimen of *Bradysia fenestralis* from Norway, on which the record in the cited literature was based, was identified in our NTI project 2014–2016.

##### Ecological note.

Oak canopies of *Quercus robur*. Phenology: Jul.

#### Bradysia flavipila

Taxon classificationAnimaliaDipteraSciaridae

Tuomikoski, 1960

6F4406B3-6C86-59BD-90C7-76723F02EBA9

##### Literature.

*Faunistics*: Vilkamaa (2000): 71 [as *Bradysia* sp.]. *Taxonomy*: Tuomikoski (1960): 144, 146; Menzel and Mohrig (2000): 125 [both as *Bradysia flavipila*].

##### Localities.

• Norway; without further locality details (= ‘Norway’) • Rogaland; Finnøy, Finnøy Island, Lasteinvatnet SE of Lastein on the SE coast (published as ‘Norway’; see faunistic note).

##### Faunistic note.

The single Norwegian record of *Bradysia flavipila* published in Vilkamaa (2000) as ‘Norway’ (without collecting details) was based on the following material: Norway • 2 ♂♂; ‘Rogaland; Finnöy, Lasteinvatnet’; 15–23 May 1994; J. Skartveit leg.; Malaise trap; UZMH.

##### Ecological note.

Habitat not specified. Phenology: May.

#### Bradysia forficulata

Taxon classificationAnimaliaDipteraSciaridae

(Bezzi, 1914)

149E94B0-4024-519A-B55E-84D2DC244585

##### Synonyms.

= luravi (Johannsen, 1929); = *nocturna* Tuomikoski, 1960.

##### Literature.

*Faunistics*: Menzel et al. (2013): 286 [as *Bradysia forficulata*]. *Taxonomy*: Tuomikoski (1960): 139, 141 [as *Bradysia nocturna*]; Mohrig and Menzel (1993): 281 [as *Bradysia forficulata*] and 283 [as *Bradysia nocturna*]; Menzel and Mohrig (2000): 119 [as *Bradysia forficulata*] and 141 [as *Bradysia nocturna*]; Menzel et al. (2013): 286; Mohrig et al. (2013): 159 [both as *Bradysia forficulata*].

##### Locality.

• Norway; without further locality details (= ‘Norway’).

##### Ecological note.

Habitat not specified. Phenology: without data.

#### Bradysia fungicola

Taxon classificationAnimaliaDipteraSciaridae

(Winnertz, 1867)

BE8B5309-FAF7-51FA-97C7-5EBE260FEE11

##### Synonyms.

= fera (Winnertz, 1867); = hercyniae (Winnertz, 1869); = *incana* (Strobl, 1910); = ingrata (Winnertz, 1867); = sylvicola (Winnertz, 1869).

##### Literature.

*Faunistics*: Soot-Ryen (1942): 78 [as *Neosciara fungicola*]. *Taxonomy*: Tuomikoski (1960): 115, 119; Menzel and Mohrig (2000): 175 [both as *Bradysia fungicola*].

##### Locality.

• Troms; Balsfjord, Fjellfrøsvatnet [Fjellfroskvannet] N of Øverbygd (= ‘Fjellfrøskvann’).

##### Ecological note.

Habitat not specified. Phenology: Jul.

#### Bradysia giraudii

Taxon classificationAnimaliaDipteraSciaridae

(Egger, 1862)

F069E299-68C8-5090-8708-511A713BDA76

##### Synonyms.

= clavigera (Lengersdorf, 1926); = *nemorum* (Winnertz, 1867).

##### Literature.

*Faunistics*: Soot-Ryen (1942): 78 [as *Neosciara nemorum*]; Menzel et al. (1990): 358 [as *Bradysia giraudii*]. *Taxonomy*: Tuomikoski (1960): 130; Menzel and Mohrig (1993b): 74 [both as *Bradysia giraudi* (Schiner); recte *giraudii* (Egger)]; Menzel and Mohrig (2000): 144 [as *Bradysia giraudii*].

##### Localities.

• Norway; without further locality details (= ‘Norwegen’) • Troms; Tromsø, Ramfjorden (= ‘Ramfjord’).

##### Ecological note.

Habitats not specified. Phenology: Jul.

#### Bradysia hilariformis

Taxon classificationAnimaliaDipteraSciaridae

Tuomikoski, 1960

8CC3548A-9A4B-5630-9DDD-43992D928BCA

##### Literature.

*Faunistics*: Köhler et al. (2014): 328 [as *Bradysia hilariformis*]. *Taxonomy*: Tuomikoski (1960): 125, 127; Menzel and Mohrig (2000): 120 [both as *Bradysia hilariformis*].

##### Locality.

• Telemark; Drangedal, woodland Steinknapp SW of Drangedal (= ‘Drangedal, Steinknapp’).

##### Faunistic note.

The first specimen of *Bradysia hilariformis* from Norway, on which the record in the cited literature was based, was identified in our NTI project 2014–2016.

##### Ecological note.

Oak canopies of *Quercus robur*. Phenology: Jun.

#### Bradysia hilaris

Taxon classificationAnimaliaDipteraSciaridae

(Winnertz, 1867)

EE29C8F0-9D1A-5B4F-9896-AAFB34787302

##### Synonyms.

= betuleti (Lengersdorf, 1940); = dolens (Johannsen, 1912); = fumida (Johannsen, 1912).

##### Literature.

*Faunistics*: Tuomikoski (1960): 125 [as *Bradysia hilaris*]. *Taxonomy*: Tuomikoski (1960): 125; Menzel and Mohrig (2000): 167; Mohrig et al. (2013): 161 [all as *Bradysia hilaris*].

##### Locality.

• Troms; Tromsø (= ‘Tromsø’).

##### Ecological note.

Habitat not specified. Phenology: Aug.

#### Bradysia impatiens

Taxon classificationAnimaliaDipteraSciaridae

(Johannsen, 1912)

89940821-4722-5A95-8047-7452958BBF6E

##### Synonyms.

= *agrestis* Sasakawa, 1978; = hardyi (Shaw, 1952); = *paupera* Tuomikoski, 1960; = tristicula var. difformis Frey, 1948.

##### Literature.

*Faunistics*: Sundbye and Johansen (2002): 26; Sundbye and Johansen (2003): 1; Sundbye (2005): 1 [all as *Bradysia difformis*]. *Taxonomy*: Tuomikoski (1960): 130, 134 [as *Bradysia paupera*]; Menzel and Mohrig (2000): 146 [as *Bradysia agrestis*] and 152 [as *Bradysia difformis*]; Mohrig et al. (2013): 162; Broadley et al. (2018): 205 [both as *Bradysia impatiens*].

##### Localities.

• Norway; without further locality details (= ‘Norway’; = ‘several horticultural localities’) • Akershus; Ås.

##### Ecological note.

In greenhouses and laboratories on poinsettia (*Euphorbia pulcherrima*). Phenology: without data.

#### Bradysia inusitata

Taxon classificationAnimaliaDipteraSciaridae

(Tuomikoski, 1960)

6BC4FCFF-E793-58B3-A873-7B921D676B9B

##### Literature.

*Faunistics*: Komarova (2009): 2 [as *Bradysia inusitata*]. *Taxonomy*: Tuomikoski (1960): 144, 148; Menzel and Mohrig (2000): 128 [both as *Bradysia inusitata*].

##### Locality.

• Norway; without further locality details (= ‘Norway’).

##### Ecological note.

Habitat not specified. Phenology: without data.

#### Bradysia iridipennis

Taxon classificationAnimaliaDipteraSciaridae

(Zetterstedt, 1838)

23BF93CC-8574-5393-8A08-733D13DBFD64

##### Synonyms.

= hirundina (Winnertz, 1867); = latiuscula (Winnertz, 1867); = merula (Winnertz, 1867); = tremulae (Beling, 1873).

##### Literature.

*Faunistics*: Zetterstedt (1855): 4890; Siebke (1877): 213 [both as *Sciara iridipennis*]; Soot-Ryen (1942): 78 [as *Neosciara iridipennis*]. *Taxonomy*: Tuomikoski (1960): 122, 124; Menzel and Mohrig (2000): 178; Mohrig et al. (2013): 163 [all as *Bradysia iridipennis*].

##### Localities.

• Norway; without further locality details (= ‘Norwegia’) • Finnmark; Alta, Jotkajavre fjellstue on the Finnmarksvidda between Karasjok and Alta (= ‘Jotkajavre’) • Nordland; Herøy, Måsvær Island (= ‘Måsvær’) • Øksnes, in the NW part of Langøya of the Vesterålen archipelago (= ‘Øksnes’) • Oslo; Oslo, Tøyen (= ‘in Tøien ad Christianiam’; = ‘Tøyen, Oslo’) • Troms; Balsfjord, Fjellfrøsvatnet [Fjellfroskvannet] N of Øverbygd (= ‘Fjellfrøskvann’) • Tromsø (= ‘Tromsø’) • Trøndelag; Verdal, farm Nes between Verdal and Lysthaugen at the S site of Verdalselva (= ‘Nes, Værdal’).

##### Ecological note.

Habitats not specified. Phenology: Jun.–Jul., Sep.

#### Bradysia lapponica

Taxon classificationAnimaliaDipteraSciaridae

(Lengersdorf, 1926)

108837FD-6357-50A9-B20C-0946513E7FE7

##### Synonyms.

= nigerrima (Lengersdorf, 1940); = pseudopraecox Frey, 1948; = quinquedentata (Lengersdorf, 1936).

##### Literature.

*Faunistics*: Tuomikoski (1960): 123; Menzel et al. (1990): 359 [both as *Bradysia lapponica*]. *Taxonomy*: Tuomikoski (1960): 122, 123; Menzel and Mohrig (2000): 145 [both as *Bradysia lapponica*].

##### Localities.

• Norway; without further locality details (= ‘Norwegen’) • Finnmark; Vardø, Varangerhalvøya, Persfjorden (= ‘Finmark: Varangerhalbinsel, Persfjord’) • Troms; Tromsø (= ‘Tromsø’).

##### Ecological note.

Habitats not specified. Phenology: Aug.

#### Bradysia longicubitalis

Taxon classificationAnimaliaDipteraSciaridae

(Lengersdorf, 1924)

BA1E6C8E-BF87-546B-A222-DAD1C0B0E754

##### Synonym.

= *cinereovittata* Frey, 1948.

##### Literature.

*Faunistics*: Komarova (2009): 3 [as *Bradysia longicubitalis*]. *Taxonomy*: Tuomikoski (1960): 138, 140 [as *Bradysia cinereovittata*]; Mohrig and Menzel (1993): 275; Menzel and Mohrig (2000): 119 [both as *Bradysia longicubitalis*].

##### Locality.

• Norway; without further locality details (= ‘Norway’).

##### Ecological note.

Habitat not specified. Phenology: without data.

#### Bradysia nervosa

Taxon classificationAnimaliaDipteraSciaridae

(Meigen, 1818)

893FDB4A-498E-5B89-9607-5394137350F1

##### Synonyms.

= fucata (Meigen, 1818); = *variabilis* (Zetterstedt, 1838).

##### Literature.

*Faunistics*: Zetterstedt (1838): 827 [as *Sciara variabilis*]; Walker (1848): 108 [as *Sciara nervosa*]; Zetterstedt (1851): 3738 [as *Sciara variabilis*]; Zetterstedt (1855): 4890 [as *Sciara nervosa* and *Sciara variabilis*]; Boheman (1866): 575; Holmgren (1869): 8 [both as *Sciara variabilis*]; Siebke (1877): 212 [as *Sciara variabilis*] and 213 [as *Sciara nervosa*]; Lengersdorf (1926b): 4 [as *Sciara nervosa*] and 9 [as *Sciara variabilis*]; Thor (1930): 4 [as *Sciara variabilis*]; Soot-Ryen (1942): 78 [in part as *Neosciara nervosa*; misidentification (only cited *variabilis* specimens)]; Coulson and Refseth (2004): 102; Coulson (2008): 160; Coulson (2013): 153 [all as *Bradysia nervosa*]. *Taxonomy*: Tuomikoski (1960): 125; Menzel and Mohrig (2000): 161 [both as *Bradysia nervosa*].

##### Localities.

• Norway; without further locality details (= ‘Norwegia’; = ‘Norwegen’; = ‘Nord-Norwegen’) • Finnmark; Hammerfest, Hammerfest (= ‘Hammerfest, Finmark’; = ‘Hammerfest’) • Karasjok, Karasjok at the river Karasjohka (= ‘Karasjok’) • Møre Og Romsdal/Oppland/Trøndelag; Dovrefjell [Dovre Mountains] (= ‘in alpe Dovre’; = ‘Dovre’) • Nordland; Narvik, Bjerkvik at the Ofotfjorden NE of Narvik (= ‘in Nordlandiæ Norvegicæ, ad diversorium Björkvik juxla Ofodenfjorden’; = ‘ad diversorium Bjørkvik prope Ofodenfjord Nordlandiae’; = ‘ad Bjŏrkvik Nordlandiae Norvegicae’; = ‘Bjørkvik, Ofoten’) • Oslo; Oslo, Tøyen (= ‘ad Christianiam in Tøien’; = ‘in Tøien ad Christianiam’; = ‘Tøien ad Christianiam’; = ‘Tøyen, Oslo’) • Oslo, Tøyenhaven (= ‘Töienhaven’; = ‘Tøyenhaven’) • Trøndelag; Verdal, farm Nes between Verdal and Lysthaugen at the S site of Verdalselva (= ‘ad Næs Værdaliæ’; = ‘ad Näs Værdaliæ’; = ‘Nes, Værdal’; = ‘Værdaliæ’) • Verdal, former poststation ‘Suulstuen’ SE of Vuku at the Jamtlandsvegen [road no. 72] (= ‘ad diversorium Suul’; = ‘ad Suul Værdaliæ’; = ‘Suul Værdaliæ’; = ‘Sul, Værdal’; = ‘Værdaliæ’).

• Svalbard; Spitsbergen, Bellsund at the W coast (= ‘ad Bel Sund’) • Spitsbergen, Edgeøya at the Storfjorden, ? Kvalpynten at the N side of the mouth of Tjuvfjorden (= ‘in Spetsbergia ad Whales Point in Storfjorden’; = ‘ad Whales Point in Storfjorden’; = ‘Whales Point, Storfjord’) • Spitsbergen, Isfjorden, Dickson Land, Kapp Thordsen (= ‘ad Cap Thordsen in Isfjorden’) • Spitsbergen, without further locality details (= ‘Spetsbergen; = ‘Spitsbergen’).

##### Ecological note.

Habitats not specified. Phenology: Apr.–Sep.

#### Bradysia nitidicollis

Taxon classificationAnimaliaDipteraSciaridae

(Meigen, 1818)

845F01DE-DFC9-552C-8DE5-2EC183595EC7

##### Synonyms.

= alacris (Winnertz, 1867); = albicans (Winnertz, 1867); = *aprilina* (Meigen, 1818); = atroparva Frey, 1948; = fenestrata (Meigen, 1818); = inornata (Winnertz, 1867); = scatopsoides (Meigen, 1818); = tenella (Winnertz, 1867); = trichoptera (Lengersdorf, 1926).

##### Literature.

*Faunistics*: Walker (1848): 108 [as *Sciara aprilina*]; Zetterstedt (1851): 3737; Zetterstedt (1855): 4890; Siebke (1877): 212 [all as *Sciara nitidicollis*]; Lengersdorf (1926b): 4 [as *Sciara nitidicollis*] and 9 [as *Sciara aprilina*]; Soot-Ryen (1942): 76 [in part as *Neosciara aprilina* (only cited Walker’s specimen)] and 79 [as *Neosciara nitidicollis*]; Tuomikoski (1960): 124; Köhler et al. (2014): 328 [both as *Bradysia nitidicollis*]. *Taxonomy*: Tuomikoski (1960): 122, 124; Menzel and Mohrig (2000): 179 [both as *Bradysia nitidicollis*].

##### Localities.

• Norway; without further locality details (= ‘Norwegia’) • Akershus; Frogn, Sønderstøa-Degerud (= ‘Degerud’) • Finnmark; Alta, Jotkajavre fjellstue on the Finnmarksvidda between Karasjok and Alta (= ‘Jotkajavre’) • Hammerfest, Hammerfest (= ‘Hammerfest, Finmark’; = ‘Hammerfest’) • Vardø, Varangerhalvøya, Persfjorden (= ‘Finmark: Varangerhalbinsel, Persfjord’) • Hordaland; Kvam, ‘Berge landskapsvernområde’ [protected landscape area with the Bergsvatnet] NW of Tørvikbygd (= ‘Kvam, Berge’) • Nordland; Sømna, Sømnes at the bay Sømnesvika N of Vik (= ‘Sømnes’) • Oslo; Oslo, Tøyen (= ‘ad Christianiam in Tøien’; = ‘Tøien’; = ‘Tøyen, Oslo’) • Østfold; Halden, Halden SE of Fredrikstad (= ‘ad Fredrikshald’) • Troms; Nordreisa, woodland and farm Hallen at the E shore of Reisaelva SE of Storslett (= ‘Nordreisa, Hallen’) • Tromsø, lake Prestvannet on the Tromsøya (= ‘Prestvann, Tromsø’) • Trøndelag; Verdal, former poststation ‘Suulstuen’ SE of Vuku at the Jamtlandsvegen [road no. 72] (= ‘ad Suul’; = ‘ad Suul Værdaliæ’; = ‘Sul, Værdal’).

##### Ecological note.

Oak canopies of *Quercus robur*; on mountains. Phenology: May–Oct.

#### Bradysia opaca

Taxon classificationAnimaliaDipteraSciaridae

(Winnertz, 1871)

46DE0F19-73DC-5703-8E05-ADBB10DE9B50

##### Synonym.

= formosa (Winnertz, 1871).

##### Literature.

*Faunistics*: Lengersdorf (1926b): 3 [as *Sciara opaca*]. *Taxonomy*: Menzel and Mohrig (2000): 166 [as *Bradysia opaca*].

##### Localities.

• Finnmark; Alta, Bojobæskihytta in the Stabbursdalen between Karasjok and Alta (= ‘Bojobæske’) • Nordland; Sørfold, Røsvik at the S shore of Sørfolda (= ‘Røsvik’) • Troms; Karlsøy, Nord-Fugløya (= ‘Nord-Fuglø’).

##### Ecological note.

Habitats not specified. Phenology: Jul.–Aug.

#### Bradysia pallipes

Taxon classificationAnimaliaDipteraSciaridae

(Fabricius, 1787)

4EED6651-F699-5E81-8308-FE76654DB344

##### Synonyms.

= agilis (Winnertz, 1867); = *brunnipes* (Meigen, 1804); = conica (Grzegorzek, 1884); = dispar (Winnertz, 1868); = engadinica (Winnertz, 1867); = *fallax* (Winnertz, 1867); = kowarzii (Grzegorzek, 1884); = laeta (Grzegorzek, 1884); = luctuosa (Winnertz, 1867); = morbosa (Winnertz, 1867); = picipes (Zetterstedt, 1838); = prolifica (Felt, 1897); = rufipodex (Frey, 1945); = rufipodex var. elysiaca (Frey, 1945); = spreta (Winnertz, 1867); = subgrandis (Shaw, 1941); = tristis (Winnertz, 1867); = umbratica (Zetterstedt, 1851).

##### Literature.

*Faunistics*: Lengersdorf (1926b): 3 [as *Sciara brunnipes*]; Soot-Ryen (1942): 77 [as *Neosciara brunnipes*]; Tuomikoski (1960): 141 [as *Bradysia brunnipes*]. *Taxonomy*: Tuomikoski (1960): 139, 141; Mohrig and Menzel (1993): 270; Menzel and Mohrig (2000): 134 [all as *Bradysia brunnipes*]; Mohrig et al. (2013): 168; Broadley et al. (2018): 226 [both as *Bradysia pallipes*].

##### Localities.

• Finnmark; Vardø, Varangerhalvøya, Persfjorden (= ‘Finmark: Varangerhalbinsel, Persfjord’) • Nordland; Øksnes, in the NW part of Langøya of the Vesterålen archipelago (= ‘Øksnes’) • Troms; Tromsø (= ‘Tromsö’; = ‘Tromsø’) • Trøndelag; Levanger, Hestøya NW of Alstahaug, southern tip Måkeskjær (= ‘Måkeskjær’).

##### Ecological note.

Habitats not specified. Phenology: Jun.–Sep.

#### Bradysia pauperata

Taxon classificationAnimaliaDipteraSciaridae

(Winnertz, 1867)

7893968A-AA5B-5C61-9FF8-A82CA8E79435

##### Synonyms.

= aestivalis (Winnertz, 1871); = antennata (Winnertz, 1867); = *lugubris* (Winnertz, 1867); = rustica (Winnertz, 1867).

##### Literature.

*Faunistics*: Lengersdorf (1926b): 3 [as *Sciara lugubris*]; Soot-Ryen (1942): 78 [in part as *Neosciara morio*; misidentification (only cited *lugubris* specimens)]. *Taxonomy*: Tuomikoski (1960): 123; Menzel and Mohrig (2000): 166 [both as *Bradysia pauperata*].

##### Localities.

• Finnmark; Alta, Bossekop in Alta (= ‘Bosekop’) • Troms; Tromsø (= ‘Tromsø’).

##### Ecological note.

Habitats not specified. Phenology: Jun.–Jul.

#### Bradysia placida

Taxon classificationAnimaliaDipteraSciaridae

(Winnertz, 1867)

6FD4CF59-ED56-592F-9D8D-79F381FE85DC

##### Synonym.

= *fimbricauda* Tuomikoski, 1960.

##### Literature.

*Faunistics*: Lengersdorf (1926b): 3 [as *Sciara placida*]; Soot-Ryen (1942): 78 [in part as *Neosciara nervosa*; misidentification (only cited *placida* specimen)]. *Taxonomy*: Tuomikoski (1960): 125, 128 [as *Bradysia fimbricauda*]; Menzel and Mohrig (2000): 162 [as *Bradysia placida*].

##### Localities.

• Finnmark; Alta, Jotkajavre fjellstue on the Finnmarksvidda between Karasjok and Alta (= ‘Jotkajavre’) • Troms; Målselv, Takvatnet (= ‘Takvand’; = ‘Takvann’).

##### Ecological note.

Habitats not specified. Phenology: Jun.–Jul.

#### Bradysia praecox

Taxon classificationAnimaliaDipteraSciaridae

(Meigen, 1818)

6CC8B617-A814-5F8A-BA81-6EE55D0AB4FD

##### Synonyms.

= *albinervis* (Winnertz, 1867); = brevipalpis (Winnertz, 1868); = leclerqi (Lengersdorf, 1950); = macilenta (Winnertz, 1867); = morosa (Winnertz, 1867); = nocticolor (Winnertz, 1867); = simplex (Winnertz, 1867); = simplex var. subsimplex (Lengersdorf, 1926); = unicolor (Winnertz, 1868).

##### Literature.

*Faunistics*: Zetterstedt (1851): 3735; Siebke (1863): 176; Siebke (1866a): 385, 388; Siebke (1877): 212; Strand (1904): 10; Summerhayes and Elton (1923): 222, 262 [all as *Sciara praecox*]; Lengersdorf (1926b): 3 [as *Sciara praecox*] and 4 [as *Sciara albinervis*]; Summerhayes and Elton (1928): 236 [as *Sciara praecox*]; Soot-Ryen (1942): 76 [in part as *Neosciara aprilina*; misidentification (only cited *albinervis* specimens)] and 79 [as *Neosciara praecox*]; Coulson and Refseth (2004): 102; Coulson (2008): 160; Coulson (2013): 153 [all as *Bradysia praecox*]. *Taxonomy*: Tuomikoski (1960): 122, 123; Menzel and Mohrig (2000): 181 [both as *Bradysia praecox*].

##### Localities.

• Norway; without further locality details (= ‘Norwegen’) • Finnmark; Karasjok, Ravnastua fjellstue NW of Karasjok (= ‘Ravnastuen’) • Porsanger, farm Fæstningsstua near Lævnasjarvi W of Skoganvarre (= ‘Fæstningstuen’; = ‘Festningsstuen’) • Møre Og Romsdal; Fræna, Hammarøya NW of Hopadalen (= ‘Hammarøy’) • Haram, ? Ormeneset (= in Romsdalia ad Ormen’; = ‘Romsdals Amt, omkring Ormen’; = ‘Ormem’) • Rauma, between Veblungsnes and Romsdalshornet Mountain in the Romsdalsalpene SE of Åndalsnes (= ‘Romsdals Amt, mellem Veblungsnæsset og Romsdalshorn’) • Rauma, Horgheim SE of Åndalsnes in the Romsdalen (= ‘in Romsdalia ad Horgheim; = ‘Romsdals Amt, Horgheim’; = ‘Horgheim’) • Rauma, Veblungsnes at the Romsdalsfjorden SW of Åndalsnes (= ‘in Romsdalia ad Veblungsnæs; = ‘Veblungsnes, Romsdal’) • Smøla, Smøla Island (= ‘in insula Smølen in Nordmøre’; = ‘ad Smölen’; = ‘Smøla’) • Nordland; Hamarøy (= ‘Hammerø’) • Oppland; Lesja, Fogstuen on the Dovrefjell plateau (= ‘Fogstuen’; = ‘ad Fokstuen’; = ‘in alpe Dovre ad Fokstuen’; = ‘in alpe Dovre’) • Oslo; Oslo (= ‘ad Christianiam’; = ‘Oslo’) • Oslo, Botanisk hage (= ‘in horto botanico ad Christianiam’; = ‘Botanical Garden, Oslo’) • Troms; Balsfjord, Øverbygd (= ‘Øverbygd’) • Karlsøy, Vannøya (= ‘Vannø’; = ‘Vannøy’) • Tromsø, Ramfjorden (= ‘Ramfjord’) • Tromsø (= ‘Tromsø’) • Trøndelag; Levanger, Skogn SE of Levanger (= ‘ad Thyæs in par. [parochia] Skogn’; = ‘ad Thyæs in Skogn’; = ‘Thynäs’; = ‘Tynes, Værdal’) [= in the accommodation of Thy in Skogn] • Meråker, NE of mountain Kølhaugan near the Swedish border [maybe a collecting place in Sweden: Jämtland, village Skalstugan close to the border with Norway] (= ‘ad diversorium Skalstugan prope jugum alpinum Norwegiæ’) • Oppdal, Kongsvoll near Kongsvold Fjeldstue in the Drivdalen (= ‘Kongsvold’; = ‘ad Kongsvold’; = ‘in alpe Dovre ad Kongsvold’; = ‘in alpe Dovre’).

• Svalbard; Bjørnøya (= ‘Bear Island, southern part’) • Spitsbergen, Aldert Dirkses Bugt in the Wijdefjorden (= ‘Spitsbergen, Aldert Dirkses Bay District [Wijde Bay]’) • Spitsbergen, Bünsow Land, Brucebyen 0.5 km S of Kapp Napier (= ‘Spitsbergen, Klaas Billen Bay (Bruce City Region), around Bruce City’).

##### Ecological note.

On beaches with *Salix polaris* and mosses; *Cassiope* heath; plant community ‘fjaeldmark’ (= feldmark; mountain field) with phanerogams, mosses, lichens and *Salix polaris* (all Svalbard records); on mountains; in botanical gardens. Phenology: Jun.–Aug.

#### Bradysia quercina

Taxon classificationAnimaliaDipteraSciaridae

Menzel & Köhler, 2014

B7A8ADB1-5AC3-5B3A-8AD8-CBE21BFA3854

##### Literature.

*Faunistics*: Köhler et al. (2014): 325 [as *Bradysia quercina*]. *Taxonomy*: Köhler et al. (2014): 325; Heller et al. (2015): 12 [both as *Bradysia quercina*].

##### Locality.

• Telemark; Drangedal, Djupedal 1.5 km SE of Henneseid (= ‘Drangedal, Djupedal, Henseid’).

##### Faunistic note.

The first specimens of *Bradysia quercina* from Norway, on which the cited literature based, were identified in our NTI project 2014–2016.

##### Ecological note.

Oak canopies of *Quercus robur*. Phenology: Jul.

#### Bradysia rufescens

Taxon classificationAnimaliaDipteraSciaridae

(Zetterstedt, 1852)

FCABA35A-D60E-5670-9890-DD1C337E7BE0

##### Synonyms.

= *pullula* (Winnertz, 1867); = somnians (Winnertz, 1867); = *testacea* (Zetterstedt, 1851) [preocc.]; = villosa (Winnertz, 1867).

##### Literature.

*Faunistics*: Zetterstedt (1851): 3763 [as *Sciara testacea*; preocc.]; Siebke (1853): 305; Zetterstedt (1855): 4890; Siebke (1863): 177 [all as *Sciara rufescens*]; Siebke (1877): 212 [as *Sciara testacea*] and 214 [as *Sciara rufescens*]; Lengersdorf (1926b): 3 [as *Sciara pullula* and *Sciara rufescens*]; Soot-Ryen (1942): 80 [as *Neosciara pullula* and *Neosciara rufescens*]; Tuomikoski (1960): 145 [as *Bradysia rufescens*]. *Taxonomy*: Zetterstedt (1852): 4545 [*Sciara rufescens* as new name for *Sciara testacea* [preocc.; not *Sciara testacea* Zetterstedt, 1838]; Tuomikoski (1960): 143, 145 [as *Bradysia rufescens*]; Menzel et al. (1990): 370 [as *Bradysia rufescens*, in part]; Menzel and Mohrig (2000): 129 [as *Bradysia rufescens*].

##### Localities.

• Finnmark; Alta, Bojobæskihytta in the Stabbursdalen between Karasjok and Alta (= ‘Bojobæske’) • Alta, Bossekop in Alta (= ‘Bosekop’) • Vardø, Vardø (= ‘Vardø’) • Møre Og Romsdal; Smøla, Smøla Island (= ‘in insula Smølen’; = ‘ad Smölen’; = ‘Smøla’) • Oppland; Dovre, Hjerkinn NW of Folldal in the Gudbrandsdalen (= ‘in alpe Dovre ad Jerkin’; = ‘Jerkin’; = ‘Hjerkin, Dovre’) • Lesja, Fogstuen on the Dovrefjell plateau (= ‘Fogstuen’; = ‘ad Fokstuen’; = ‘Fokstuen, Dovre’; = ‘in alpe Dovre ad Fokstuen’; = ‘in alpe Dovre’) • Øyer in the Gudbrandsdalen (= ‘in parochiis Øyer … Gudbrandsdaliæ’; = ‘Gudbrandsdalen, Öier’; = ‘Öier’; = ‘Øyer’) • Ringebu in the Gudbrandsdalen (= ‘in parochiis … Ringebo Gudbrandsdaliæ’; = ‘Gudbrandsdalen, Ringebo’; = ‘Ringebo’; = ‘Ringebu, Gudbrandsdal’) • Oslo; Oslo, Botanisk hage (= ‘in horto botanico ad Christianiam’) • Oslo, Tøyen (= ‘ad Töien prope Christianiam’; = ‘Töien nahe Kristiania [Oslo]’) • Troms; Karlsøy, Torsvåg at the NW coast of Vannøya 15 km N of Tromsø (= ‘Torsvaag’) • Nordreisa, woodland and farm Hallen at the E shore of Reisaelva SE of Storslett (= ‘Nordreisa, Hallen’; = ‘Nordreisa’) • Tromsø (= ‘Tromsø’) • Trøndelag; Levanger, Hestøya NW of Alstahaug, southern tip Måkeskjær (= ‘Måkeskjær’) • Oppdal, in the Drivdalen (= ‘Drivdalen’) • Oppdal, Kongsvoll near Kongsvold Fjeldstue in the Drivdalen (= ‘Kongsvold’; = ‘ad Kongsvold’; = ‘Kongsvold, Dovre’; = ‘in alpe Dovre ad Kongsvold’; = ‘in alpe Dovre’).

##### Taxonomic note.

After Lengersdorf (1930a: 52) and Tuomikoski (1960: 145) *Sciara testacea* Zetterstedt, described in Zetterstedt (1838: 826), does not belong to the Sciaridae, but to the Diadocidiidae [= *Diadocidia testacea* (Zetterstedt, 1938)]. Zetterstedt (1851: 3763) later describes another ‘*Sciara testacea*’ [preocc.; not *Sciara testacea* Zetterstedt, 1838], which without doubt belongs to the Sciaridae and was renamed by Zetterstedt himself as *Sciara rufescens* [see Zetterstedt (1852: 4545)]. Siebke (1877: 212) used the name ‘*Sciara testacea* Zetterstedt’ in connection with Zetterstedt’s original description in volume ‘X’ of ‘Diptera scandinaviae disposita et descripta’ (Zetterstedt 1851). For that reason the citation by Siebke (1877) and the Norwegian record on page 212 is preliminarily included here.

In the here presented checklist of Norwegian Sciaridae, ***Bradysia vagans* (Winnertz, 1868)** is missing, with its synonyms *B. angustipennis* Frey, 1948 [preocc.], *B. callicera* Frey, 1948 and *B. richardi* Gerbachevskaja, 1986. This is a very common species throughout Europe. It is dark brown, with rather broad wings and unicoloured dark-brown antennae, but is not distinguishable by the male genitalia from the reddish-yellow *Bradysia rufescens* (Zetterstedt). It is possible, that there are some misidentified specimens of *Bradysia vagans* (Winnertz) among the records of ‘*Bradysia rufescens*’, published before Tuomikoski (1960).

##### Ecological note.

In the grass in humid places; in botanical gardens. Phenology: Jun.–Aug.

#### Bradysia sordida

Taxon classificationAnimaliaDipteraSciaridae

(Zetterstedt, 1838)

6D698182-2AF0-527C-8A02-BE35059F72AD

##### Literature.

*Faunistics*: Siebke (1863): 176; Siebke (1877): 212 [both as *Sciara sordida*]. *Taxonomy*: Menzel and Mohrig (2000): 185 [as *Bradysia sordida*].

##### Localities.

• Norway; without further locality details (= ‘Norwegen’) • Oppland; Dovre, Hjerkinn NW of Folldal in the Gudbrandsdalen (= ‘in alpe Dovre, ad Jerkin’; = ‘Jerkin’) • Trøndelag; Oppdal, in the Drivdalen (= ‘Drivdalen’) • Oppdal, Kongsvoll near Kongsvold Fjeldstue in the Drivdalen (= ‘in alpe Dovre ad Kongsvold’).

##### Ecological note.

Habitats not specified. Phenology: Jul.–Aug.

#### Bradysia strenua

Taxon classificationAnimaliaDipteraSciaridae

(Winnertz, 1867)

56032FDC-1A49-5936-9262-AAE5D6F3FD83

##### Synonyms.

= *annulata absoloni* (Bezzi, 1911); = ardua (Grzegorzek, 1884); = watsoni Colless, 1962.

##### Literature.

*Faunistics*: Broadley et al. (2018): 230 [as *Bradysia strenua*]. *Taxonomy*: Mohrig and Menzel (1993): 283; Menzel and Mohrig (2000): 142; Broadley et al. (2018): 229 [all as *Bradysia strenua*].

##### Locality.

• Norway; without further locality details (= ‘Norway’).

##### Ecological note.

Habitat not specified. Phenology: without data.

#### Bradysia strigata

Taxon classificationAnimaliaDipteraSciaridae

(Staeger, 1840)

5B08F214-31AB-5C7E-9FA6-D8B21CA70C70

##### Synonym.

= robusta (Lengersdorf, 1926) [preocc.].

##### Literature.

*Faunistics*: Zetterstedt (1851): 3747 [as *Sciara strigata*] and 3749 [as *Sciara persicariae* (Linnaeus); misidentification]; Siebke (1863): 177; Siebke (1870): 304; Siebke (1877): 213 [all as *Sciara persicariae* sensu Zetterstedt; misidentification]; Menzel et al. (1990): 372 [as *Bradysia strigata*]. *Taxonomy*: Frey (1948): 54, 77; Tuomikoski (1960): 144, 149; Menzel and Mohrig (1993b): 77; Menzel and Mohrig (2000): 130 [all as *Bradysia strigata*].

##### Localities.

• Norway; without further locality details (= ‘in jugo alpino Norwegiæ’; = ‘in Norwegia’; = ‘Norwegen’) • Oppland; Dovre, Hjerkinn NW of Folldal in the Gudbrandsdalen (= ‘in alpe Dovre, ad Jerkin’; = Dovre ad Jerkin; = ‘Jerkin’; = ‘Hjerkin, Dovre’) • Lesja, Fogstuen on the Dovrefjell plateau (= ‘Dovre ad Fogstuen’; = ‘Fogstuen’) • Vang, Nystuen at the Otrøvatnet NW of Vang (= ‘in alpibus Filefjeld ad Nystuen; = ‘Nystuen’) • Trøndelag; Oppdal, Kongsvoll near Kongsvold Fjeldstue in the Drivdalen (= ‘Dovre ad Kongsvold’; = ‘Dovre ad Kongsvoll’) • Verdal, Kjølhaugan mountain SE of Sul, close to the Swedish border (= ‘in summo cacumine alpis … Kälahög (4000 ped. supra mare elevato)’; = ‘in summo cacumine alpis Kælahøg Værdaliae’).

##### Taxonomic note.

The taxon ‘*Tipula persicariae*’ was originally described by Linnaeus (1767: 977). The revision of the types revealed, that this species belongs to the gall midges and was placed in the genus *Wachtiella* Rübsaamen, 1915 [= *Wachtliella persicariae* (Linnaeus, 1767); Cecidomyiidae: Cecidomyiinae, Dasineurini]. Soot-Ryen (1942: 74) interpreted ‘*Sciara persicariae* (Linnaeus)’ in Zetterstedt (1871) and Siebke (1863, 1870, 1877) as a species of ‘*Dasyneura*’ [recte ‘*Dasineura*’ (Cecidomyiidae)] and ignored therefore all records of ‘*Sciara persicariae*’ in his list of Norwegian Sciaridae. Frey (1948: 54, 77) states however, that some specimens of ‘*Sciara persicariae* (Linnaeus)’ sensu Zetterstedt were interpreted incorrectly and might belong to *Bradysia strigata* (Staeger) [misidentification]. One specimen in the TMUC collection (belonging to Sciaridae) was found under ‘*sororcula* Zetterstedt’ (det. Siebke), which – following the synonymy of types – would be *Bradysia confinis* (Winnertz). We are following Frey (1948) and list under *Bradysia strigata* (Staeger) all Norwegian records until a final revision of misidentified ‘*Sciara persicariae* (Linnaeus)’ sensu Zetterstedt is undertaken.

##### Ecological note.

On sides and peaks of mountains, up to 4,000 ft (1,219 m). Phenology: Jul.–Aug.

#### Bradysia tilicola

Taxon classificationAnimaliaDipteraSciaridae

(Loew, 1850)

7778FC54-5236-53DA-BDD5-3869FAE4FD29

##### Synonyms.

= *amoena* (Winnertz, 1867); = alma (Winnertz, 1871); = caldaria (Lintner, 1895); = coprophila (Lintner, 1895); = domestica Frey, 1948; = incomta (Winnertz, 1867); = marcilla (Hutton, 1902); = nanella (Frey, 1936); = selecta (Winnertz, 1871); = setigera (Winnertz, 1867); = *silvatica* (Meigen, 1818); = sexdentata (Pettey, 1918); = triseriata (Winnertz, 1867); = turbida (Winnertz, 1867); = vana (Winnertz, 1871); = vividula (Winnertz, 1867); = volucris (Winnertz, 1867); = wendalinae (van Bruggen, 1954).

##### Literature.

*Faunistics*: ? Schøyen (1889) 14; ? Trail (1889) 203, 215 [both as *Sciara tilicola*]; Soot-Ryen (1942) 78 [as *Neosciara modesta*; misidentification] and 80 [as *Sciara silvatica*]. *Taxonomy*: Tuomikoski (1960): 130, 132; Menzel and Mohrig (2000): 147 [both as *Bradysia amoena*]; Menzel and Heller (2005): 351; Menzel et al. (2013): 286; Mohrig et al. (2013): 171; Broadley et al. (2018): 224 [all as *Bradysia tilicola*].

##### Localities.

• Finnmark; Alta, Jotkajavre fjellstue on the Finnmarksvidda between Karasjok and Alta (= ‘Jotkajavre’) • ? Hordaland; Hardanger, Granvin, Eide [= ‘vicinity of Eide’; = ‘Eide i Hardanger’ = ‘Eide, Hardanger’) • Oslo; Oslo, Tøyen (= ‘Tøyen, Oslo’) • Troms; Balsfjord, Øverbygd (= ‘Øverbygd’) • Tromsø (= ‘Tromsø’) • Trøndelag; Levanger, Hestøya NW of Alstahaug, southern tip Måkeskjær (= ‘Måkeskjær’) • Verdal, Tromsdal SE of Lysthaugen (= ‘Tromsdal’).

##### Ecological note.

On twig of *Tilia parvifolia* [questionable record based on galls]. Phenology: Jun.–Oct.

#### Bradysia trivittata

Taxon classificationAnimaliaDipteraSciaridae

(Staeger, 1840)

F772A0B7-C9DD-57D1-AD74-0C5603904AA7

##### Synonyms.

= basalis (Winnertz, 1867); = decipiens (Winnertz, 1867); = devittata Tuomikoski, 1959; = lignorum (Kieffer, 1919); = spectrum (Winnertz, 1867); = versicolorea (Lengersdorf, 1940).

##### Literature.

*Faunistics*: Siebke (1877): 215 [as *Sciara trivittata*]. *Taxonomy*: Tuomikoski (1960): 130, 133; Menzel and Mohrig (2000): 156 [both as *Bradysia trivittata*].

##### Locality.

• Oslo; Oslo, Botanisk hage (= ‘in horto botanico ad Christianiam’).

##### Ecological note.

In botanical gardens. Phenology: Jun.

#### Bradysia vernalis

Taxon classificationAnimaliaDipteraSciaridae

(Zetterstedt, 1851)

0FCEE81C-C89D-5FF0-9E19-434AB5C28DC3

##### Synonyms.

= monticola (Winnertz, 1867); = *vallestris* (Lengersdorf, 1926).

##### Literature.

*Faunistics*: Lengersdorf (1926b): 5 [as *Sciara vallestris*]; Soot-Ryen (1942): 80 [as *Neosciara vernalis*]; Menzel et al. (1990): 377 [as *Bradysia vernalis*]. *Taxonomy*: Tuomikoski (1960): 123, 124; Menzel and Mohrig (2000): 183 [both as *Bradysia vernalis*].

##### Localities.

• Norway; without further locality details (= ‘Norwegen’) • Finnmark; Porsanger, farm Fæstningsstua near Lævnasjarvi W of Skoganvarre (= ‘Fæstningsstuen’; = ‘Festningsstuen’) • Troms; Karlsøy, Vannøya (= ‘Vannö’; = ‘Vannø’; = ‘Vannøy’).

##### Ecological note.

Habitats not specified. Phenology: Jul.–Aug.

#### Bradysiopsis vittigera

Taxon classificationAnimaliaDipteraSciaridae

(Zetterstedt, 1851)

F698D58A-9406-50E2-967C-3493AA1638BE

##### Literature.

*Faunistics*: Zetterstedt (1851): 3751; Siebke (1877): 213 [both as *Sciara vittigera*]; Soot-Ryen (1942): 80 [as *Neosciara vittigera*; in part]; Menzel and Mohrig (2000): 189 [as *Bradysiopsis vittigera*]. *Taxonomy*: Tuomikoski (1960): 74 [as Lycoriella (Bradysiopsis) vittigera]; Menzel and Mohrig (1998): 361; Menzel and Mohrig (2000): 189 [both as *Bradysiopsis vittigera*].

##### Localities.

• Norway; without further locality details (= ‘in Norwegia’; = ‘Norwegia [Norwegen]’) • Oslo; Oslo (= ‘ad Christianiam’; = ‘Oslo’) • Oslo, Bekkelaget (= ‘Bækkelaget propre Christ.’; = ‘Bekkelaget’) • Østfold; Halden, Halden SE of Fredrikstad (= ‘ad Fredrikshald’; = ‘Fredrikshald’).

##### Ecological note.

Habitats not specified. Phenology: May, Jul.

#### Camptochaeta bournei

Taxon classificationAnimaliaDipteraSciaridae

(Shaw, 1941)

790385E3-E645-5A4A-9115-B26FCAB47C7E

##### Synonym.

= *subvivax* (Mohrig, 1985).

##### Literature.

*Faunistics*: Hippa and Vilkamaa (1994): 29 [as *Camptochaeta bournei*]. *Taxonomy*: Mohrig (1985): 233 [as *Corynoptera subvivax*]; Hippa and Vilkamaa (1994): 29; Menzel and Mohrig (2000): 194; Mohrig et al. (2013): 174 [all as *Camptochaeta bournei*].

##### Locality.

• Finnmark; Vardø, Varangerhalvøya, Persfjorden (= ‘Persfjord, Varranger’).

##### Ecological note.

Habitat not specified. Phenology: without data.

#### Camptochaeta camptochaeta

Taxon classificationAnimaliaDipteraSciaridae

(Tuomikoski, 1960)

5E2FE799-939C-56AC-B722-649A7F920945

##### Literature.

*Faunistics*: Hippa and Vilkamaa (1994): 27; Thunes et al. (2004): 72, 85 [both as *Camptochaeta camptochaeta*]. *Taxonomy*: Tuomikoski (1960): 67, 69 [as *Corynoptera camptochaeta*]; Hippa and Vilkamaa (1994): 27; Menzel and Mohrig (2000): 195; Komarova et al. (2007): 4 [all as *Camptochaeta camptochaeta*].

##### Localities.

• Norway; without further locality details (= ‘Norway’) • Buskerud; Sigdal, Heimseteråsen (= ‘Sigdal’) • Finnmark; Alta, Leirbotn at the E side of Altafjorden (= ‘Leirbotn’) • Porsanger, Børselv NE of Lakselv at the E coast of Porsangerfjorden (= ‘Börselv’).

##### Ecological note.

*Pinus sylvestris* dominated boreal forests with *Betula pubescens* and *Picea abies*. Phenology: Jun.–Jul.

#### Camptochaeta consimilis

Taxon classificationAnimaliaDipteraSciaridae

(Holmgren, 1869)

6A9DD237-72DE-520E-BB99-2B3EACBE6607

##### Synonym.

= *glacialis* (Rübsaamen, 1898)

##### Literature.

*Faunistics*: Holmgren (1869): 6, 16, 54 [as *Sciara consimilis*]; Edwards (1922): 198; Edwards (1924): 164 [both as *Sciara praecox*; misidentification]; Lengersdorf (1930a): 55 [as *Sciara consimilis* and *Sciara ecalcarata*; misidentification]; Lengersdorf (1930c): 52 [as *Sciara ecalcarata* sensu Lengersdorf; misidentification, and *Sciara glacialis* Rübsamer; recte Rübsaamen]; Edwards (1935): 534; Bertram and Lack (1938): 51 [both as *Sciara consimilis*]; Soot-Ryen (1942): 78 [as *Neosciara glacialis*]; Tuomikoski (1967): 46 [as *Corynoptera consimilis*]; Hippa and Vilkamaa (1994): 12 [as *Camptochaeta consimilis*]; Menzel and Mohrig (2000): 197 [as *Camptochaeta consimilis*] and 198 [as *Camptochaeta consimilis* in the discussion of *Camptochaeta delicata*]; Coulson and Refseth (2004): 102; Komarova et al. (2007): 6; Coulson (2008): 161; Coulson (2013): 153; Mohrig et al. (2013): 174 [all as *Camptochaeta consimilis*]. *Taxonomy*: Hippa and Vilkamaa (1994): 12; Menzel and Mohrig (2000): 197; Mohrig et al. (2013): 174 [all as *Camptochaeta consimilis*].

##### Localities.

• Norway; without further locality details (= ‘Norway’) • Finnmark; Sør-Varanger, Bugøyfjord (= ‘Buköyfjord’) • ? Troms; Tromsø (= ‘Tromsø’).

• Svalbard; Bjørnøya (= ‘Beeren Eiland’; = ‘Beeren Island’; = ‘Bear Island’) • Bjørnøya, Gravodden [grave point (graveyard)] at the N coast (= ‘Bear Island, Gravodden’) • Bjørnøya, Haussvatnet in the N part of island (= ‘Bear Island, Hausvatnet’) • Bjørnøya, Kvalrossbukta [formerly ‘Hvalrosbugten’] at the SE side of island (= ‘Bear Island, Walrus Bay, S.E.’) • Bjørnøya, Laksvatnet in the N part of island (= ‘Bear Island, Laksvatnet’) • Bjørnøya, near the Steelva at the Laksvatnet in the N part of island (= ‘bei Steelva’, am Laksvatnet (B.)’) • Bjørnøya, Røyevatnet in the SW part of island (= ‘Bear Island, Röyevatnet’) • Spitsbergen, Adventdalen near Adventfjorden at the W coast (= ‘Spitzbergen, Adventdalen’; = ‘Adventdalen’) • Spitsbergen, Adventfjorden at the W coast (= ‘in Spetsbergia ad Advent Bay’; = ‘Spetsbergia ad Advent Bay’ [Spitzbergen, bei der Advent Bay]’; = ‘Spitsbergen, near Advent Bay’; = ‘Spitzbergen, Advent Bay’) • Spitsbergen, Albert I Land, Cape Flathuken on the Vasahalvøya (= ‘Spitzbergen, Flathuken’; = ‘Flathuken’) • Spitsbergen, Albert I Land, strait Sørgattet between Reuschhalvøya and Danskøya (= ‘Sørgattet’; = ‘Sörgatt’) • Spitsbergen, Billefjorden between Dickson Land and Bünsow Land (= ‘head of Billefjorden [Klaas Billen Bay]’) • Spitsbergen, Bünsow Land, Brucebyen 0.5 km S of Kapp Napier (= ‘Spitsbergen, Bruce City, head of Klaas Billen Bay’; = ‘Brucebyen [Bruce City]’) • Spitsbergen, Grønfjorden, Barentsburg (= ‘Barentsburg’; = ‘bei Barentsburg (S.)’) • Spitsbergen, Haakon VII Land, Liefdefjorden (= ‘N. Spitsbergen, Liefde Bay’) • Spitsbergen, Haakon VII Land at the NW coast, S side of Reinsdyrflya (= ‘middle of S. side of Reindeer Peninsula’; = ‘middle of S. side of Reinsdyrflya [Reindeer Peninsula]’) • Spitsbergen, Hiorthhamn [former mining settlement] at the E side of Adventfjorden (= ‘Hiorthhamn (S.), bei Residensen’) • Spitsbergen, Kobbefjorden at the NW coast near the Danskøya (= ‘in Spetsbergia ad Kobbebay’; = ‘Kobbefjorden [Kobbebay]’; = ‘Kobbebay’) • Spitsbergen, Longyearbyen (= ‘Spitzbergen, Longyearbyen’; = ‘Longyearbyen auf Spitzbergen’; = ‘Longyearbyen’) • Spitsbergen, Nordenskiöld Land, Helvetiadalen between the mountains Helvetiafjellet and Artowskifjellet N of Adventdalen (= ‘front face of Helvetiadalen’) • Spitsbergen, Nordenskiöld Land, Mälardalen at the N side of the mouth of Adventelva (= ‘Mælardalen’) • Spitsbergen, Ny-Ålesund (= ‘Spitsbergen, Ny Ålesund’) • Spitsbergen, Ny-Friesland, Dirksbukta at the S side of the Dirksodden (= ‘N. Spitsbergen, Albert Dirkses Bay’; = ‘Dirksbukta [Albert Dirkses Bay]’) • Spitsbergen, S coast of Kongsfjorden along the N side of Brøggerhalvøya, W of Ny-Ålesund [= ‘NW Spitsbergen, South cost Königsfjord, W Ny Ålesund’] • Spitsbergen, Sassen-Bünsow Land, Sassendalen (= ‘Sassendalen’) • Spitsbergen, Wijdefjorden (= ‘N. Spitsbergen, Wijde Bay’; = ‘Wijdefjorden [Wijde Bay]’) • Spitsbergen, without further locality details (= ‘Spetsbergen’; = ‘Spitzbergen’; = ‘Spitsbergen’).

##### Taxonomic note.

The female holotype of *Sciara glacialis* Rübsaamen was studied by the senior author and identified as a junior synonym to *Camptochaeta consimilis* (Holmgren). More detailed information will be presented in a separate publication about the *Sciara* species described by Rübsaamen (1898).

##### Ecological note.

Bird cliffs; in mosses, lichens and *Salix* plants; on *Cerastium alpinum*, *Salix polaris* and *Cassiope*; on shingly raised beaches with *Dryas*; among stones; on bare rocks (all Svalbard records). Phenology: Jun.–Aug.

#### Camptochaeta delicata

Taxon classificationAnimaliaDipteraSciaridae

(Lengersdorf, 1935)

129E91A7-BDF7-52F7-84CB-B00CAE34FFFF

##### Synonyms.

= *macrodon* (Frey, 1948); = *pallidiventris* (Holmgren, 1869) [preocc.].

##### Literature.

*Faunistics*: Holmgren (1869): 15, 53 [as *Sciara pallidiventris*]; Edwards (1922): 196; Edwards (1924): 164; Summerhayes and Elton (1923): 262; Edwards (1925): 354; Summerhayes and Elton (1928): 209, 218, 228, 236; Lengersdorf (1930a): 55 [all as *Sciara pallidiventris*]; Edwards (1935): 534 [as *Sciara* sp. indet. and *Sciara pallidiventris*]; Lengersdorf (1935): 75; Soot-Ryen (1942): 77 [both as *Neosciara delicata*]; Frey (1948): 86, 91 [as Bradysia (Diorychophthalma) macrodon]; Tuomikoski (1967): 47 [as *Corynoptera macrodon*] and 50 [as *Sciara delicata*]; Hippa and Vilkamaa (1994): 36 [as *Camptochaeta delicata*]; Menzel and Mohrig (2000): 197 [as *Camptochaeta delicata* and *Sciara pallidiventris*]; Coulson and Refseth (2004): 102 [as *Camptochaeta delicata* (Frey); recte (Lengersdorf)]; Hågvar et al. (2007): 67; Komarova et al. (2007): 6 [both as *Corynoptera delicata*]; Coulson (2008): 161; Coulson (2013): 154 [both as *Camptochaeta delicata* (Frey); recte (Lengersdorf)]; Mohrig et al. (2013): 174 [as *Camptochaeta delicata*]. *Taxonomy*: Hippa and Vilkamaa (1994): 36; Menzel and Mohrig (2000): 197; Mohrig et al. (2013): 174 [all as *Camptochaeta delicata*].

##### Localities.

• Norway; without further locality details (= ‘Norway’) • Finnmark; Karasjok, 20 km N of Karasjok (= ‘20 km N of Karasjok’).

• Svalbard; Bjørnøya (= ‘Bear Island’) • Bjørnøya, Brettingsdalen at the E side of Miseryfjellet (= ‘Bear Island, Brettingsdalen’) • Spitsbergen, Adventdalen, Fivelflyane 8 km E of Longyearbyen (= ‘Adventdalen, Fivelflyane’) • Spitsbergen, Adventfjorden at the W coast (= ‘in Spetsbergia ad Advent Bay’; = ‘Spetsbergia, Advent Bay’; = ‘Spitzbergen, bei der Advent Bay’; = ‘Adventfjorden [Advent Bay]’) • Spitsbergen, Aldert Dirkses Bugt in the Wijdefjorden (= ‘Spitsbergen, Aldert Dirkses Bay District [Wijde Bay]’) • Spitsbergen, Billefjorden between Dickson Land and Bünsow Land (= ‘head of Billefjorden [Klaas Billen Bay]’) • Spitsbergen, Bünsow Land, Brucebyen 0.5 km S of Kapp Napier (= ‘Spitsbergen, Bruce City, head of Klaas Billen Bay’; = ‘Spitsbergen, Klaas Billen Bay (Bruce City Region), around Bruce City’; = ‘Bruce City, Klaas Billen Bay (S.)’; = ‘Brucebyen [Bruce City]’) • Spitsbergen, Haakon VII Land, Bockfjorden at the W side of Woodfjorden (= ‘Bockfjorden’) • Spitsbergen, Haakon VII Land, Reinsdyrflya, at the Liefdefjorden (= ‘Spitsbergen, Reindeer Peninsula, at the Liefde Bay’; = ‘N. Spitsbergen, Liefde Bay’; = ‘Liefdefjorden [Liefde Bay]’) • Spitsbergen, Haakon VII Land, S side and centre of Reinsdyrflya (= ‘West Spitsbergen Island, south side and centre of the east half of Reindeer Peninsula’) • Spitsbergen, Isfjorden (= ‘Isfjorden’) • Spitsbergen, Isfjorden, Dickson Land, Kapp Thordsen (= ‘in Spetsbergia ad Cap Torsden in Isfjorden’; = ‘Cap Torsden’) • Spitsbergen, Kobbefjorden at the NW coast near the Danskøya (= ‘in Spetsbergia ad Kobbebay’; = ‘Kobbefjorden [Kobbebay]’) • Spitsbergen, Longyearbyen (= ‘Spitzbergen, Longyearbyen’; = ‘Longyearbyen auf Spitzbergen’; = ‘Longyearbyen’) • Spitsbergen, Nordaustlandet (= ‘Spitsbergen, North-East Land’) • Spitsbergen, Nordenskiöld Land, Arctowskifjellet mountain S of Sassenfjorden (= ‘Arctowskifjellet’) • Spitsbergen, Nordenskiöld Land, Helvetiadalen between the mountains Helvetiafjellet and Artowskifjellet N of Adventdalen (= ‘front face of Helvetiadalen’) • Spitsbergen, Ny-Friesland, Dirksbukta at the S side of the Dirksodden (= ‘Dirksbukta [Aldert Dirkses Bay]’; = ‘N. Spitsbergen, Albert Dirkses Bay’) • Spitsbergen, Sassen-Bünsow Land, Sassendalen (= ‘Sassendalen’) • Spitsbergen, Sigridholmen, Kongsfjorden • Spitsbergen, Wijdefjorden (= ‘N. Spitsbergen, Wijde Bay’; = ‘Wijdefjorden [Wijde Bay]’) • Spitsbergen, without further locality details (= ‘Spetsbergen; = ‘Spitsbergen’; = ‘Spitzbergen’).

##### Ecological note.

From plants on flower slopes; *Dryas* community on mountain slopes (*Dryas octopetala*, *Carex misandra*, *Cerania vermicularis*, *Cetraria nivalis*); over leaves and flowers of *Dryas* plants; *Cassiope* heath; lichen-moss heath; plant community ‘fjaeldmark’ (= feldmark; mountain field) with phanerogams, mosses, lichens and *Salix polaris*; on *Saxifraga oppositifolia*; from grass; under stones with some vegetation; on beaches with *Salix polaris* and mosses; on shingly raised beaches with *Dryas* (all Svalbard records). Phenology: Jul.–Aug.

#### Camptochaeta fallax

Taxon classificationAnimaliaDipteraSciaridae

Hippa & Vilkamaa, 1994

2CBAD233-338D-567C-8123-49FEC1E60850

##### Literature.

*Faunistics*: Hippa and Vilkamaa (1994): 19; Rudzinski and Ševčík (2012): 146 [both as *Camptochaeta fallax*]. *Taxonomy*: Hippa and Vilkamaa (1994): 19; Menzel and Mohrig (2000): 194 [both as *Camptochaeta fallax*].

##### Localities.

• Norway; without further locality details (= ‘Norway’) • Finnmark; Lebesby, at the Matselva (= ‘Mattselva’) • Porsanger, Børselv NE of Lakselv at the E coast of Porsangerfjorden (= ‘Börselv’).

##### Ecological note.

Habitats not specified. Phenology: Jul.

#### Camptochaeta hirtula

Taxon classificationAnimaliaDipteraSciaridae

(Lengersdorf, 1934)

E7E716CC-2407-5164-B0EE-7D5514305D36

##### Synonym.

= *fulvicollis* (Tuomikoski, 1960).

##### Literature.

*Faunistics*: Hippa and Vilkamaa (1994): 14 [as *Camptochaeta fulvicollis*]; Thunes et al. (2004): 85 [as *Camptochaeta hirtula*]. *Taxonomy*: Tuomikoski (1960): 67 [as *Corynoptera fulvicollis*]; Hippa and Vilkamaa (1994): 14 [as *Camptochaeta fulvicollis*]; Menzel and Mohrig (2000): 198; Mohrig et al. (2013): 176 [both as *Camptochaeta hirtula*].

##### Localities.

• Buskerud; Sigdal, Heimseteråsen (= ‘Sigdal’) • Finnmark; Kvalsund, Skaidi (= ‘Skaidi’) • Sør-Varanger, Bugøyfjord (= ‘Buköyfjord’) • Sør-Varanger, Neiden (= ‘Neiden’) • Troms; Nordreisa, Sappen (= ‘Sappen’).

##### Ecological note.

*Pinus sylvestris* dominated boreal forests with *Betula pubescens* and *Picea abies*. Phenology: Jun.–Aug.

#### Camptochaeta mimica

Taxon classificationAnimaliaDipteraSciaridae

Hippa & Vilkamaa, 1994

19D7ED35-7A8C-510A-8C30-E718D213DA20

##### Literature.

*Faunistics*: Mohrig et al. (2013): 176 [as *Camptochaeta mimica*]. *Taxonomy*: Hippa and Vilkamaa (1994): 39; Mohrig et al. (2013): 176 [both as *Camptochaeta mimica*].

##### Locality.

• Svalbard; Spitsbergen, Ny-Ålesund (= ‘Spitsbergen, Ny Ålesund’).

##### Ecological note.

Habitat not specified. Phenology: Jul.

#### Camptochaeta truncata

Taxon classificationAnimaliaDipteraSciaridae

Vilkamaa & Mohrig, 2013

6D2D06AB-EAB6-5DCD-AE91-ECD22EB8621A

##### Literature.

*Faunistics*: Vilkamaa et al. (2013a): 484 [as *Camptochaeta truncata*]. *Taxonomy*: Vilkamaa et al. (2013a): 484 [as *Camptochaeta truncata*].

##### Locality.

• Svalbard; Spitsbergen, S coast of Kongsfjorden along the N side of Brøggerhalvøya, W of Ny-Ålesund [= ‘Spitzbergen, southern cost of Königsfjord, west of Ny Ölesund’].

##### Ecological note.

Habitat not specified. Phenology: Jul.

#### Camptochaeta xystica

Taxon classificationAnimaliaDipteraSciaridae

Hippa & Vilkamaa, 1994

7BC04E7F-C61A-5720-B5B5-76D0DC96CBD0

##### Literature.

*Faunistics*: Hippa and Vilkamaa (1994): 44 [as *Camptochaeta xystica*]. *Taxonomy*: Hippa and Vilkamaa (1994): 44; Menzel and Mohrig (2000): 194 [both as *Camptochaeta xystica*].

##### Locality.

• Finnmark; Tana, Storfossen at the river Karasjohka near the Finnish border (= ‘Tana, Nedre Storfoss’).

##### Ecological note.

Habitat not specified. Phenology: Jul.

#### Chaetosciara estlandica

Taxon classificationAnimaliaDipteraSciaridae

(Lengersdorf, 1929)

1A06B91A-F187-5036-BA2A-B4391A2C9723

##### Synonym.

= *lengersdorfi* (Frey, 1948).

##### Literature.

*Faunistics*: Staverløkk and Sæthre (2007): 16, 36; Sæthre et al. (2010): 28, 31 [both as *Chaetosciara estlandica*]. *Taxonomy*: Tuomikoski (1960): 41; Menzel and Mohrig (2000): 202 [both as *Chaetosciara estlandica*].

##### Locality.

• Norway; without further locality details (= ‘Norway, imported from the Netherlands’).

##### Ecological note.

On plants of *Taxus media*. Phenology: Apr.

#### Claustropyga brevichaeta

Taxon classificationAnimaliaDipteraSciaridae

(Mohrig & Antonova, 1978)

F1850EBA-F1D9-53AA-8B3F-C06E3C508268

##### Literature.

*Faunistics*: Hippa et al. (2003): 488; Vilkamaa et al. (2013): 22 [both as *Claustropyga brevichaeta*]. *Taxonomy*: Menzel and Mohrig (2000): 222 [as *Corynoptera brevichaeta*]; Hippa et al. (2003): 488 [as *Claustropyga brevichaeta*].

##### Localities.

• Norway; without further locality details (= ‘Norway’) • Trøndelag; Oppdal, Kongsvoll near Kongsvold Fjeldstue in the Drivdalen (= ‘Oppdal, Kungsvoll’).

##### Ecological note.

Habitats not specified. Phenology: Jun.–Jul.

#### Claustropyga refrigerata

Taxon classificationAnimaliaDipteraSciaridae

(Lengersdorf, 1930)

89EF698B-E387-5ED0-B924-9F67CC4B2385

##### Synonym.

= scandinavica (Rudzinski, 1992).

##### Literature.

*Faunistics*: Lengersdorf (1930b): 3; Soot-Ryen (1942): 80 [both as *Neosciara refrigerata*]; Tuomikoski (1960): 47; Menzel and Mohrig (2000): 250 [both as *Corynoptera refrigerata*]; Hippa et al. (2003): 502 [as *Claustropyga refrigerata*]. *Taxonomy*: Tuomikoski (1960): 43, 46; Menzel and Mohrig (2000): 250 [both as *Corynoptera refrigerata*]; Hippa et al. (2003): 501 [as *Claustropyga refrigerata*].

##### Localities.

• Norway; without further locality details (= ‘Nordnorwegen’) • Finnmark; ? Tana, Hangalacærro mountain near Austertana (= ‘Finnmark, Caerro’) • Troms; Balsfjord, Fjellfrøsvatnet [Fjellfroskvannet] N of Øverbygd (= ‘Fjellfrøskvann’) • Balsfjord, Øverbygd (= ‘Øverbygd’) • Kvænangen (= ‘Kvaenangen’) • Tromsø (= ‘Tromsø’) • Tromsø, lake Prestvannet on the Tromsøya (= ‘Prestvann, Tromsø’) • Tromsø, Ramfjorden (= ‘Ramfjord’) [misinterpretation in Menzel and Mohrig (2000), not ‘Ramsøyfjord zwischen den Inseln Smøla und Hitra’].

##### Ecological note.

Habitats not specified. Phenology: Jun.–Aug.

#### Corynoptera boletiphaga

Taxon classificationAnimaliaDipteraSciaridae

(Lengersdorf, 1940)

A9A74932-16B1-5DF5-8ECC-26AC135045B0

##### Synonyms.

= filiceti (Frey, 1948); = *geogenia* Tuomikoski, 1960.

##### Literature.

*Faunistics*: Thunes et al. (2004): 72, 85 [as *Corynoptera boletiphaga*]; Hippa et al. (2010): 177 [as Corynoptera (Corynoptera) boletiphaga]. *Taxonomy*: Tuomikoski (1960): 49, 61; Mohrig (1978): 426; Menzel and Mohrig (2000): 250 [all as *Corynoptera boletiphaga*]; Hippa et al. (2010): 176 [as Corynoptera (Corynoptera) boletiphaga].

##### Localities.

• Buskerud; Sigdal, Heimseteråsen (= ‘Buskerud, Sigdal’; = ‘Sigdal’) • Finnmark; Sør-Varanger, near Neiden (= ‘nr. Neiden’) • Vardø, Vardø (= ‘Vardsø’).

##### Ecological note.

*Pinus sylvestris* dominated boreal forests with *Betula pubescens* and *Picea abies*; birch forest with shrubs. Phenology: Jun.–Aug.

#### Corynoptera brachypennis

Taxon classificationAnimaliaDipteraSciaridae

(Lengersdorf, 1926)

304127D0-915F-5332-8097-B821CFF478A8

##### Literature.

*Faunistics*: Lengersdorf (1926b): 4; Lengersdorf (1928–30): 22; Soot-Ryen (1942): 75 [all as *Bradysia brachypennis*]; Mohrig and Mamaev (1970): 353; Mohrig et al. (1978): 398; Menzel and Mohrig (2000): 260 [all as *Corynoptera brachypennis*]. *Taxonomy*: Menzel and Mohrig (2000): 260 [as *Corynoptera brachypennis*].

##### Localities.

• Norway; without further locality details (= ‘Norwegia’; = ‘Norwegen’) • Troms; Tromsø (= ‘Tromsö’; = ‘Tromsø’; = ‘Umgebung Tromsø’).

##### Ecological note.

Habitats not specified. Phenology: May.

#### Corynoptera defecta

Taxon classificationAnimaliaDipteraSciaridae

(Frey, 1948)

A2EB5D57-A28D-56C7-94CE-385D82FD6A0F

##### Literature.

*Faunistics*: Hippa et al. (2010): 174 [as Corynoptera (Corynoptera) defecta]. *Taxonomy*: Tuomikoski (1960): 49, 60 [as Plastosciara (Plastosciara) defecta under *Corynoptera bistrispina*]; Menzel and Mohrig (2000): 250 [in part as *Corynoptera bistrispina*; misidentification]; Hippa et al. (2010): 174 [as Corynoptera (Corynoptera) defecta].

##### Locality.

• Finnmark; Kvalsund, Skaidi (= ‘Skvalsund, Skaidi’).

##### Ecological note.

Habitat not specified. Phenology: Jul.

#### Corynoptera fatigans

Taxon classificationAnimaliaDipteraSciaridae

(Johannsen, 1912)

94C700AB-5922-5CC5-A134-92E4E386DF53

##### Synonyms.

= bicornis (Lengersdorf, 1943); = *perpusilla* Winnertz, 1867 [preocc.].

##### Literature.

*Faunistics*: Soot-Ryen (1942): 79 [as *Neosciara perpusilla*]. *Taxonomy*: Menzel and Mohrig (2000): 223; Hippa et al. (2010): 21 [both as *Corynoptera perpusilla*]; Mohrig et al. (2013): 183 [as *Corynoptera fatigans*].

##### Localities.

• Troms; Balsfjord, Fjellfrøsvatnet [Fjellfroskvannet] N of Øverbygd (= ‘Fjellfrøskvann’) • Tromsø, Ramfjorden (= ‘Ramfjord’) • Trøndelag; Verdal, Tromsdal SE of Lysthaugen (= ‘Tromsdal’).

##### Ecological note.

Habitats not specified. Phenology: Jun.–Jul.

#### Corynoptera flavicauda

Taxon classificationAnimaliaDipteraSciaridae

(Zetterstedt, 1855)

2F326FCB-88D0-5F59-BCDE-729697FA4AB0

##### Literature.

*Faunistics*: Lengersdorf (1926b): 3 [as *Sciara flavicauda*]; Soot-Ryen (1942): 77 [as *Neosciara flavicauda*]; Menzel et al. (1990): 382 [as *Corynoptera flavicauda*]. *Taxonomy*: Tuomikoski (1960): 48, 52; Menzel and Mohrig (2000): 255 [both as *Corynoptera flavicauda*]; Hippa et al. (2010): 119 [as Corynoptera (Corynoptera) flavicauda].

##### Localities.

• Norway; without further locality details (= ‘Norwegen’) • Oslo; Oslo, Tøyen (= ‘Tøien’; = ‘Tøyen’) • Troms; Tromsø (= ‘Tromsø’) • Tromsø, Ramfjorden (= ‘Ramfjord’).

##### Ecological note.

Habitats not specified. Phenology: Jun.–Jul.

#### Corynoptera forcipata

Taxon classificationAnimaliaDipteraSciaridae

(Winnertz, 1867)

AF73D7DE-3661-57DD-BD10-859C4F2387CF

##### Literature.

*Faunistics*: Köhler et al. (2014): 328 [as *Corynoptera forcipata*]. *Taxonomy*: Tuomikoski (1960): 64, 65; Menzel and Mohrig (2000): 247 [both as *Corynoptera forcipata*].

##### Localities.

• Hordaland; Kvam, point Skeianeset at the N shore of the Hardangerfjorden SW of Indre Ålvik (= ‘Kvam, Skeianeset’) • Telemark; Drangedal, 300 m SE of Henneseid (= ‘Drangedal, Henseid’) • Drangedal, woodland Steinknapp SW of Drangedal (= ‘Drangedal, Steinknapp’).

##### Faunistic note.

The first specimens of *Corynoptera forcipata* from Norway were identified in our NTI project 2014–2016.

##### Ecological note.

Oak canopies of *Quercus robur*. Phenology: Jun.–Jul.

#### Corynoptera hypopygialis

Taxon classificationAnimaliaDipteraSciaridae

(Lengersdorf, 1926)

78D59C9E-CF72-5AB7-8A2E-B2A7CFFCAED6

##### Synonyms.

= pachycerca (Frey, 1948); = *piniphila* (Lengersdorf, 1940).

##### Literature.

*Faunistics*: Tuomikoski (1960): 52 [as *Corynoptera piniphila*]; Köhler et al. (2014): 328 [as *Corynoptera hypopygialis*]. *Taxonomy*: Tuomikoski (1960): 48, 52 [as *Corynoptera piniphila*]; Menzel and Mohrig (1993b): 72; Menzel and Mohrig (2000): 256 [both as *Corynoptera hypopygialis*]; Hippa et al. (2010): 121 [as Corynoptera (Corynoptera) hypopygialis].

##### Localities.

• Finnmark; Vardø, Varangerhalvøya, Persfjorden (= ‘Vardö, Persfjord’) • Telemark; Drangedal, 300 m SE of Henneseid (= ‘Drangedal, Henseid’) • Drangedal, woodland Steinknapp SW of Drangedal (= ‘Drangedal, Steinknapp’).

##### Ecological note.

Oak canopies of *Quercus robur*. Phenology: Jun.–Aug.

#### Corynoptera irmgardis

Taxon classificationAnimaliaDipteraSciaridae

(Lengersdorf, 1930)

06A6C076-B261-5A52-8097-F5FBAAEAD2A1

##### Literature.

*Faunistics*: Köhler et al. (2014): 329 [as *Corynoptera irmgardis*]. *Taxonomy*: Tuomikoski (1960): 49, 57; Menzel and Mohrig (2000): 225 [both as *Corynoptera irmgardis*]; Hippa et al. (2010): 100 [as Corynoptera (Corynoptera) irmgardis].

##### Locality.

• Telemark; Porsgrunn, Mule Varde SE of Porsgrunn at the Eidangerfjorden (= ‘Porsgrunn, Mule Varde’).

##### Faunistic note.

The first specimen of *Corynoptera irmgardis* from Norway was identified in our NTI project 2014–2016.

##### Ecological note.

Oak canopies of *Quercus robur*. Phenology: Jul.

#### Corynoptera membranigera

Taxon classificationAnimaliaDipteraSciaridae

(Kieffer, 1903)

C49F8779-E075-533C-8F26-1AACA991EF1D

##### Synonym.

= *trispina* Tuomikoski, 1960.

##### Literature.

*Faunistics*: Köhler et al. (2014): 329 [as *Corynoptera membranigera*]. *Taxonomy*: Tuomikoski (1960): 49, 63 [as *Corynoptera trispina*]; Menzel and Mohrig (2000): 230 [as *Corynoptera membranigera*]; Hippa et al. (2010): 153 [as Corynoptera (Corynoptera) membranigera].

##### Localities.

• Hordaland; Kvam, point Skeianeset at the N shore of the Hardangerfjorden SW of Indre Ålvik (= ‘Kvam, Skeianeset’) • Telemark; Drangedal, Djupedal 1.5 km SE of Henneseid (= ‘Drangedal, Djupedal, Henseid’) • Drangedal, woodland Steinknapp SW of Drangedal (= ‘Drangedal, Steinknapp’) • Vestfold; Larvik, lake Skjærsjø near Kvelde NW of Larvik (= ‘Larvik, Skjærsjø’).

##### Faunistic note.

The first specimens of *Corynoptera membranigera* from Norway were identified in our NTI project 2014–2016.

##### Ecological note.

Oak canopies of *Quercus robur*. Phenology: Jun.–Jul.

#### Corynoptera minima

Taxon classificationAnimaliaDipteraSciaridae

(Meigen, 1818)

19B0785E-56B5-5807-84E6-6A2206642AFB

##### Synonyms.

= *brachyptera* (Lengersdorf, 1941); = brevipennis (Walker, 1848).

##### Literature.

*Faunistics*: Zetterstedt (1851): 3749; Siebke (1877): 213 [both as *Sciara minima*]; Soot-Ryen (1942): 78 [as *Neosciara minima*]; Thunes et al. (2004): 85 [as *Corynoptera minima*]; Hippa et al. (2010): 189 [as Corynoptera (Corynoptera) minima]. *Taxonomy*: Tuomikoski (1960): 61, 62 [as *Corynoptera brachyptera* in the discussion of *Corynoptera geogenia*]; Mohrig (1978): 427 [as *Corynoptera brachyptera*]; Menzel and Mohrig (2000): 253 [as *Corynoptera minima*]; Hippa et al. (2010): 188 [as Corynoptera (Corynoptera) minima].

##### Localities.

• Norway; without further locality details (= ‘Norwegiæ’) • Buskerud; Sigdal, Heimseteråsen (= ‘Sigdal’) • Oslo; Oslo, Botanisk hage (= ‘in horto botanico ad Christianiam’; = ‘Botanical Garden, Oslo’).

##### Ecological note.

*Pinus sylvestris* dominated boreal forests with *Betula pubescens* and *Picea abies*; in botanical gardens. Phenology: Apr., Jun.–Jul.

#### Corynoptera montana

Taxon classificationAnimaliaDipteraSciaridae

(Winnertz, 1869)

B8DDE8E8-5CDE-53AD-BF9E-7DBB5066708E

##### Synonym.

= fusca (Winnertz, 1871).

##### Literature.

*Faunistics*: Hippa et al. (2010): 57 [as Corynoptera (Corynoptera) montana]. *Taxonomy*: Tuomikoski (1960): 48, 50; Menzel and Mohrig (2000): 256 [both as *Corynoptera montana*]; Hippa et al. (2010): 57 [as Corynoptera (Corynoptera) montana].

##### Locality.

• Finnmark; Kvalsund, Kvalsund (= ‘Kvalsund’).

##### Ecological note.

Habitat not specified. Phenology: Jul.

#### Corynoptera penna

Taxon classificationAnimaliaDipteraSciaridae

(Pettey, 1918)

E7F5F3E5-B0C2-5AF1-BF23-83A1EBE8E9A0

##### Synonym.

= *alneti* Hippa, Vilkamaa & Heller, 2010.

##### Literature.

*Faunistics*: Hippa et al. (2010): 25 [as Corynoptera (Corynoptera) alneti]; Mohrig et al. (2013): 189 [as Corynoptera (Corynoptera) penna]. *Taxonomy*: Hippa et al. (2010): 25 [as Corynoptera (Corynoptera) alneti]; Mohrig et al. (2013): 189 [as Corynoptera (Corynoptera) penna].

##### Locality.

• Finnmark; Sør-Varanger, Kirkenes (= ‘Kirkenes’).

##### Ecological note.

Forest with birch, willow and bushes. Phenology: Jul.

#### Corynoptera roederi

Taxon classificationAnimaliaDipteraSciaridae

(Lengersdorf, 1931)

40B508E7-43EC-5A28-9828-F209A535F95D

##### Literature.

*Faunistics*: Lengersdorf (1931): 65 [as *Neosciara röderi*; recte *roederi*]; Bertram and Lack (1938): 51 [as *Sciara röderi*; recte *roederi*]; Menzel and Mohrig (1993a): 54 [as Lycoriella (Lycoriella) roederi]; Menzel and Mohrig (2000): 257 [as *Corynoptera roederi*]; Coulson and Refseth (2004): 103; Coulson (2008): 161; Coulson (2013): 154 [all as *Corynoptera röderi*; recte *roederi*]. *Taxonomy*: Menzel and Mohrig (1993a): 54 [as Lycoriella (Lycoriella) roederi]; Menzel and Mohrig (2000): 257 [as *Corynoptera roederi*].

##### Locality.

• Svalbard; Bjørnøya (= ‘Bäreninsel’; = ‘Bear Island’).

##### Ecological note.

Habitats not specified. Phenology: without data.

#### Corynoptera saetistyla

Taxon classificationAnimaliaDipteraSciaridae

Mohrig & Krivosheina, 1985

C100CD15-3D51-53FD-AE26-E365F0EC0900

##### Synonym.

= densiseta Mohrig & Menzel, 1990.

##### Literature.

*Faunistics*: Hippa et al. (2010): 114 [as Corynoptera (Corynoptera) saetistyla]. *Taxonomy*: Menzel and Mohrig (2000): 226 [as *Corynoptera saetistyla*]; Hippa et al. (2010): 113; Mohrig et al. (2013): 192 [both as Corynoptera (Corynoptera) saetistyla].

##### Localities.

• Norway; without further locality details (= ‘Norway’) • Finnmark; Vardø, Vardø (= ‘Vardsø’).

##### Ecological note.

Birch forest with shrubs. Phenology: Jul.

#### Corynoptera sphenoptera

Taxon classificationAnimaliaDipteraSciaridae

Tuomikoski, 1960

F7860C8C-80FF-52BA-AAB2-0C5C5D310C99

##### Literature.

*Faunistics*: Hippa et al. (2010): 35 [as Corynoptera (Corynoptera) sphenoptera]. *Taxonomy*: Tuomikoski (1960): 49, 58; Menzel and Mohrig (2000): 227 [both as *Corynoptera sphenoptera*]; Hippa et al. (2010): 34; Mohrig et al. (2013): 192 [both as Corynoptera (Corynoptera) sphenoptera].

##### Locality.

• Finnmark; Sør-Varanger, Kirkenes (= ‘Kirkenes’).

##### Ecological note.

Forest with birch, willow and bushes. Phenology: Jul.

#### Corynoptera spoeckeri

Taxon classificationAnimaliaDipteraSciaridae

(Lengersdorf, 1930)

252A8FAE-DF45-5594-B325-AC2F800C6717

##### Synonym.

= venerata Rudzinski, 1994.

##### Literature.

*Faunistics*: Menzel et al. (1990): 389 [as *Corynoptera spoeckeri*]. *Taxonomy*: Menzel and Mohrig (2000): 249 [as *Corynoptera spoeckeri*].

##### Locality.

• Norway; without further locality details (= ‘Norwegen’).

##### Ecological note.

Habitat not specified. Phenology: without data.

#### Corynoptera subtilis

Taxon classificationAnimaliaDipteraSciaridae

(Lengersdorf, 1929)

ADDFF3C4-2AB1-5995-8E2D-E8D88B04582A

##### Synonyms.

= *longicornis* (Bukowski & Lengersdorf, 1936); = signhildae (Frey, 1948).

##### Literature.

*Faunistics*: Hippa et al. (2010): 93 [as Corynoptera (Corynoptera) subtilis]. *Taxonomy*: Tuomikoski (1960): 49, 57 [as *Corynoptera longicornis*]; Menzel and Mohrig (2000): 228 [as *Corynoptera subtilis*]; Hippa et al. (2010): 92 [as Corynoptera (Corynoptera) subtilis].

##### Localities.

• Finnmark; Båtsfjord, Varangerhalvøya, Ytre Syltefjord 35 km SE of Båtsfjord (= ‘Varanger peninsula, Ytre, Syltefjord, 35 km SE Batsfjord’) • Sør-Varanger, Svanvik 40 km S of Kirkenes (= ‘Svanvik’) • Vardø, Vardø (= ‘Vardsø’) • Nordland; Nessna, Nessna in Helgeland (= ‘Nessna’).

##### Ecological note.

Mixed forest (pine, birch); birch forest with shrubs; dwarf-shrub tundra. Phenology: Jul.

#### Corynoptera subvariegata

Taxon classificationAnimaliaDipteraSciaridae

Rudzinski, 1992

A677CCC0-F230-5A45-B568-4CA6F421756D

##### Literature.

*Faunistics*: Vilkamaa et al. (2013b): 329 [as *Corynoptera subvariegata*]. *Taxonomy*: Menzel and Mohrig (2000): 221; Vilkamaa et al. (2013b): 329 [both as *Corynoptera subvariegata*].

##### Locality.

• Troms; Nordreisa, Sappen (= ‘Sappen’).

##### Ecological note.

Habitat not specified. Phenology: Jul.

#### Corynoptera trepida

Taxon classificationAnimaliaDipteraSciaridae

(Winnertz, 1867)

F52B48B7-D93E-58C5-98DA-6ECA01D93A07

##### Synonyms.

= *clinochaeta* Tuomikoski, 1960; = subflava (Lengersdorf, 1941).

##### Literature.

*Faunistics*: Thunes et al. (2004): 72, 85 [as *Corynoptera trepida*]; Hippa et al. (2010): 96 [as Corynoptera (Corynoptera) trepida]. *Taxonomy*: Tuomikoski (1960): 49, 52 [as *Corynoptera clinochaeta*]; Menzel and Mohrig (2000): 230 [as *Corynoptera trepida*]; Hippa et al. (2010): 95; Mohrig et al. (2013): 194 [both as Corynoptera (Corynoptera) trepida].

##### Localities.

• Buskerud; Sigdal, Heimseteråsen (= ‘Sigdal’) • Hedmark; Trysil, Fulufjellet mountain near Ljørdalen (= Ljørdal, way to Fulufjället’) • Rogaland; Finnøy, Finnøy Island, Lasteinvatnet SE of Lastein at the SE coast (= ‘RY, Finnöy, Ledsteinvatnet’).

##### Ecological note.

*Pinus sylvestris* dominated boreal forests with *Betula pubescens* and *Picea abies*. Phenology: Apr.–Aug.

#### Corynoptera waltraudis

Taxon classificationAnimaliaDipteraSciaridae

Mohrig & Mamaev, 1987

A3C80FF2-DCAA-5ADA-A341-E393BB00A680

##### Literature.

*Faunistics*: Hippa et al. (2010): 91 [as Corynoptera (Corynoptera) waltraudis]. *Taxonomy*: Menzel and Mohrig (2000): 221 [as *Corynoptera waltraudis*]; Hippa et al. (2010): 91 [as Corynoptera (Corynoptera) waltraudis].

##### Localities.

• Finnmark; Berlevåg, Varangerhalvøya, Kjølnes fyr (= ‘Varanger Peninsula, Kjölnes fyr’) • Sør-Varanger, Svanvik 40 km S of Kirkenes (= ‘Svanvik’) • Trøndelag; Oppdal, stream Sprenbekken NE of Kongsvold Fjeldstue in the Drivdalen (= ‘Oppdal, Kongsvoll, Sprenbekken’).

##### Ecological note.

Mixed forests (pine, birch); meadows at coasts. Phenology: Jul.–Aug.

#### Cratyna (Cratyna) ambigua

Taxon classificationAnimaliaDipteraSciaridae

(Lengersdorf, 1934)

668856E1-60D5-5C18-807A-5BB687D71362

##### Synonyms.

= *latiforceps* (Bukowski & Lengersdorf, 1936); = lignea (Lengersdorf, 1941); = prima (Frey, 1942).

##### Literature.

*Faunistics*: Köhler et al. (2014): 329 [as Cratyna (Cratyna) ambigua]. *Taxonomy*: Tuomikoski (1960): 32 [as Plastosciara (Decembrina) latiforceps]; Menzel and Mohrig (1998): 363; Menzel and Mohrig (2000): 272 [both as Cratyna (Cratyna) ambigua].

##### Locality.

• Hordaland; Kvam, ‘Berge landskapsvernområde’ [protected landscape area with the Bergsvatnet] NW of Tørvikbygd (= ‘Kvam, Berge’).

##### Faunistic note.

The first specimen of *Cratyna ambigua* from Norway was identified in our NTI project 2014–2016.

##### Ecological note.

Oak canopies of *Quercus robur*. Phenology: Jun.

#### Cratyna (Cratyna) atra

Taxon classificationAnimaliaDipteraSciaridae

Winnertz, 1867

FA786BE4-71CA-5DC9-9B25-548DA66E12F7

##### Synonyms.

= corticalis (Lengersdorf, 1930); = ericia (Pettey, 1918); = lugens (Johannsen, 1912); = macclurei (Shaw, 1941); = *pictiventris* (Kieffer, 1898).

##### Literature.

*Faunistics*: Lengersdorf (1926a): 253 [as *Sciara pictiventris*]; Lengersdorf (1926b): 4; Soot-Ryen (1942): 75 [both as *Plastosciara pictiventris*]. *Taxonomy*: Tuomikoski (1960): 33, 34 [as Plastosciara (Plastosciara) pictiventris]; Menzel and Mohrig (1998): 363; Menzel and Mohrig (2000): 271; Mohrig et al. (2013): 196 [all as Cratyna (Cratyna) atra].

##### Localities.

• Norway; without further locality details (= ‘N. = Norwegen’) • Finnmark; Alta, Bossekop in Alta (= ‘Bosekop’).

##### Ecological note.

Habitats not specified. Phenology: May–Jul.

#### Cratyna (Cratyna) hirticornis

Taxon classificationAnimaliaDipteraSciaridae

(Meigen, 1818)

D78E956A-CDB3-5C86-9046-CED08C2CD053

##### Literature.

*Faunistics*: Zetterstedt (1851): 3753; Siebke (1877): 214 [both as *Sciara hirticornis*]; Soot-Ryen (1942): 80 [in part as *Scatopsciara vitripennis* (only cited *hirticornis* specimen)]. *Taxonomy*: Menzel and Mohrig (1998): 363; Menzel and Mohrig (2000): 274 [both as Cratyna (Cratyna) hirticornis].

##### Locality.

• Trøndelag; Verdal, former poststation ‘Suulstuen’ SE of Vuku at the Jamtlandsvegen [road no. 72] (= ‘ad Suul’; = ‘ad Suul in Værdalen’; = ‘Sul, Værdal’).

##### Ecological note.

Habitat not specified. Phenology: Jul.

#### Cratyna (Cratyna) longipennis

Taxon classificationAnimaliaDipteraSciaridae

(Lengersdorf, 1931)

AA77D932-2911-56FF-9CE8-FD85ACCA5D6D

##### Literature.

*Faunistics*: Lengersdorf (1931): 66 [as *Plastosciara longipennis*]; Menzel and Mohrig (1993a): 56 [as Plastosciara (Plastosciara) longipennis]; Menzel and Mohrig (2000): 275 [as Cratyna (Cratyna) longipennis]. *Taxonomy*: Menzel and Mohrig (1993a): 56 [as Plastosciara (Plastosciara) longipennis]; Menzel and Mohrig (2000): 275 [as Cratyna (Cratyna) longipennis].

##### Locality.

• Svalbard; Bjørnøya (= ‘Bäreninsel’).

##### Ecological note.

Habitat not specified. Phenology: without data.

#### Cratyna (Cratyna) uliginosa

Taxon classificationAnimaliaDipteraSciaridae

(Lengersdorf, 1929)

87D22FEA-995B-5E92-B54E-CBE73079C7D9

##### Literature.

*Faunistics*: Thunes et al. (2004): 72, 85 [as *Cratyna uliginosa*]; Heller et al. (2016): 100 [as Cratyna (Cratyna) uliginosa]. *Taxonomy*: Tuomikoski (1960): 32, 33 [as Plastosciara (Decembrina) uliginosa]; Menzel and Mohrig (1998): 363; Menzel and Mohrig (2000): 277; Heller et al. (2016): 98 [all as Cratyna (Cratyna) uliginosa].

##### Localities.

• Akershus; Asker, Sem NW of Asker, Tangen Peninsula at the E side of Semsvannet (= ‘Asker, Sem, Tangen’) • Aust-Agder; Birkenes, Birkeland, Nordåsen. Lillesand, Lillesand, Furulia • Buskerud; Sigdal, Heimseteråsen (= ‘Sigdal’) • Finnmark; Sør-Varanger, Neiden • Hedmark; Elverum, Starmoen naturreservat SE of Elverum (= ‘Starrmoen NR’) • Stor-Elvdal, N of Krokmyra, at a cabin E of Fåfengtjørna (= ‘N Krokmyra – Ved hytta, E Fåfengtjørna’) • Hordaland; Bergen, Bergen, Fløyen mountain, mountain top Fløyfjellet (= Bergen, Fløyfjellet) • Stord, NE coast of Stord Island, SW part of Hageberg SE of Vistvik (= ‘Hageberg SV – SE of Vistvik, NE coast of Stord’) • Møre Og Romsdal; Ørskog, Nysætra, near the Nysætervatnet NE of Sjøholt (= ‘Nysætra – NE of Sjøholt, near Nysætervatnet’) • Sogn Og Fjordane; Jølster, Hamarsvika, Jølstravatnet NE of Vassenden (= ‘Hamarsvika – NE of Vassenden, Jølstravatnet’) • Vestfold; Larvik, Farmenrøysa mountain NE of Kvelde (= ‘Larvik, Farmenrøysa Ø’ [correctly: ‘Farmenrøysa, east-facing slope’]) • Larvik, hill Småås N of Larvik (= ‘Larvik, Småås’) • Larvik, Nevlungstranda W of Nevlunghavn, beach Mølen (= ‘Mølen’) • Re, Revetal, Våle.

##### Ecological note.

On sandy beaches and hillsides; east- and south-facing mountain slopes with damp meadows (downy birch, dwarf birch, scots pines, blueberry, rushes, sedges, mosses) and deadwood-rich mixed forests (grey alder, downy birch, rowan, Norway spruce); swampy old spruce forests; in the damp ground vegetation (blueberry, ferns, grasses, mosses) with small springs; *Pinus sylvestris* dominated boreal forests with *Betula pubescens* and *Picea abies*. Phenology: May–Sep.

#### Cratyna (Cratyna) uliginosoides

Taxon classificationAnimaliaDipteraSciaridae

Heller, Köhler & Menzel, 2016

E6751CCB-5C40-534A-80F3-884775E0B2F7

##### Literature.

*Faunistics*: Heller et al. (2016): 102, 103, 104 [as Cratyna (Cratyna) uliginosoides]. *Taxonomy*: Heller et al. (2016): 102 [as Cratyna (Cratyna) uliginosoides].

##### Localities.

• Akershus; Ullensaker, Sessvollmoen N of Moen (= ‘Sessvollmoen – N Moen’) • Aust-Agder; Evje og Hornnes, Klepsland • Buskerud; Sigdal, Heimseteråsen (= ‘Sigdal, Furukrone Nr. 1’ [correctly translated from Norwegian: ‘Sigdal, crown of pine tree no. 1’]) • Hordaland; Stord, NE coast of Stord Island, SW part of Hageberg SE of Vistvik (= ‘Hageberg SV – SE of Vistvik, NE coast of Stord’) • Sveio, Langemyr SE of Sveio (= ‘Langemyr – SE of Sveio’) • Møre Og Romsdal; Molde, N part of Julaksla mountain W of Mek (= ‘Julaksla N – W of Mek’) • Vestnes, Småøyane SE of Kristisetra, SE of Vestnes (= ‘Vestnes, Småøyane, SE of Kristisetra [SE of Vestnes]’) • Volda, at the Øyraelva. Sogn Og Fjordane; Høyanger, NE of Austreim at the N side of Sognefjorden, N of hill Furehaugen (= ‘N Furehaugen’) • Telemark; Bamble, Langøya in the Langesundsfjorden, bay at the E side of island (= ‘Langøya – Bukt på østsiden (Langøya I)’ [correctly translated from Norwegian: ‘Langøya, bay at the eastern side (Langøya I)’]) • Tinn, Hovin NW of Kongsberg, Spjeldset SW of Øvre Fjellstul (= ‘Hovin, Spjeldset’) • Trøndelag; Trondheim, Trondheim, Sommerlystvegen (= ‘Sør-Trøndelag, Trondheim, M. Sommerlystvegen – in the garden of nr. 22’) • Vestfold; Horten, Borre, Adaltjern naturreservat NW of Bakkenteigen (= ‘Adaltjern, Bakkenteigen’) • Larvik, hill Småås N of Larvik (= ‘Larvik, Småås’).

##### Faunistic note.

The first specimens of *Cratyna uliginosoides* from Norway were collected and/or identified in our NTI project 2014–2016. Erroneously Heller et al. (2016) listed the specimen with the no. BAB415020 twice: one time correctly as *Cratyna uliginosa* and one time falsely as paratype of *Cratyna uliginosoides*. Therefore the record of *Cr. uliginosoides* in Hedmark is not reliable.

##### Ecological note.

On woody hillsides and in mountain birch forests; pine forests (e.g. *Pinus sylvestris* dominated boreal forests with *Betula pubescens* and *Picea abies*); forests with oak, birch, juniper, blueberry and wavy hair-grass; mixed forests (scots pine, Norway spruce, downy birch, common hazel, juniper) with ferns and mosses; mixed forests on steep mountain slopes with crevices and cavities (scots pine, Norway spruce, downy birch, grey alder, rowan, juniper, heather, blueberry, cotton grass, marsh orchids, rushes, mosses); in bogs, otherwise muddy terrain and deadwood-rich carrs along streams and near rivers (downy birch, grey alder, rowan, juniper, rushes, sedges, mosses, lichens); deadwood-rich deciduous forests (common hazel, grey alder, sycamore maple, rowan, birch, ferns, mosses); in gardens with lawn and some larger trees, also on waste. Phenology: May–Sep.

#### Cratyna (Spathobdella) colei

Taxon classificationAnimaliaDipteraSciaridae

(Freeman, 1990)

6F240862-AA3C-55DB-A98F-862C007125ED

##### Literature.

*Faunistics*: Tuomikoski (1960): 27; Menzel et al. (1990): 321 [both as Plastosciara (Spathobdella) brachialis sensu Tuomikoski; misidentification]. *Taxonomy*: Tuomikoski (1960): 35, 37 [as Plastosciara (Spathobdella) brachialis; misidentification]; Rudzinski (1994): 17 [as *Plastosciara brachialis* sensu Tuomikoski; misidentification]; Freeman (1990): 52 [as Plastosciara (Spathobdella) colei]; Menzel and Mohrig (2000): 281 [as Cratyna (Spathobdella) colei].

##### Localities.

• Norway; without further locality details (= ‘Norwegen’) • Finnmark; Tana, Tanafjorden, fjord Vestertana (= ‘Finmark, Vestertana’) • Tana, upper part of the Langfjordelva (= ‘Finmark, am oberen Lauf des Flusses Langfjordelva’) • Vardø, Varangerhalvøya, Persfjorden (= ‘Finmark, Varangerhalbinsel, Persfjord’).

##### Ecological note.

Habitats not specified. Phenology: Aug.

#### Cratyna (Spathobdella) falcata

Taxon classificationAnimaliaDipteraSciaridae

(Tuomikoski, 1960)

B6C85254-B13D-57E8-B7E6-8B36E4BC54E6

##### Literature.

*Faunistics*: Tuomikoski (1960): 39; Menzel et al. (1990): 321 [both as Plastosciara (Spathobdella) falcata]. *Taxonomy*: Tuomikoski (1960): 35, 39; Mohrig (1978): 430 [both as Plastosciara (Spathobdella) falcata]; Menzel and Mohrig (2000): 270 [as Cratyna (Spathobdella) falcata].

##### Localities.

• Norway; without further locality details (= ‘Norwegen’) • Finnmark; Tana, Tanafjorden, fjord Vestertana (= ‘Finmark, Vestertana’) • Vardø, Varangerhalvøya, Persfjorden (= ‘Vardø, Persfjord’).

##### Ecological note.

Habitats not specified. Phenology: Aug.

#### Cratyna (Spathobdella) longispina

Taxon classificationAnimaliaDipteraSciaridae

(Pettey, 1918)

D63799F1-F265-5E7F-8D90-B904155DCD7E

##### Synonym.

= *tuberculata* (Tuomikoski, 1960).

##### Literature.

*Faunistics*: Tuomikoski (1960): 39 [as Plastosciara (Spathobdella) tuberculata]; Mohrig et al. (2013): 199 [as Cratyna (Spathobdella) tuberculata under Cratyna (Spathobdella) longispina]; Shin et al. (2014): 352 [as Cratyna (Spathobdella) longispina]. *Taxonomy*: Tuomikoski (1960): 37, 39 [as Plastosciara (Spathobdella) tuberculata]; Menzel and Mohrig (2000): 270 [as Cratyna (Spathobdella) tuberculata]; Mohrig et al. (2013): 199 [as Cratyna (Spathobdella) longispina].

##### Localities.

• Norway; without further locality details (= ‘Norway’) • Finnmark; Tana, upper part of the Langfjordelva between Porsangerfjorden and fjord Vestertana (= ‘Finmark, am oberen Lauf des Flusses Langfjordelva zwischen Porsangerfjord und Vestertana’; = ‘Finnmark, river Langfjordelva between Porsangerfjord and Vestertana’).

##### Ecological note.

Habitats not specified. Phenology: Aug.

#### Cratyna (Spathobdella) nobilis

Taxon classificationAnimaliaDipteraSciaridae

(Winnertz, 1867)

4683A52A-C91B-507A-A132-DFEE54806D09

##### Synonyms.

= *brachialis* (Winnertz, 1867); = cunctans (Winnertz, 1871).

##### Literature.

*Faunistics*: Lengersdorf (1926b): 3 [as *Sciara nobilis*]; Soot-Ryen (1942): 79 [as *Neosciara nobilis*]; Tuomikoski (1960): 39 [as Plastosciara (Spathobdella) nobilis]. *Taxonomy*: Tuomikoski (1960): 35, 38 [as Plastosciara (Spathobdella) nobilis]; Menzel and Mohrig (2000): 280 [as Cratyna (Spathobdella) nobilis].

##### Localities.

• Finnmark; Tana, Tanafjorden, fjord Vestertana (= ‘Finmark, Vestertana’) • Vardø, Varangerhalvøya, Persfjorden (= ‘Finmark, Vardø, Persfjord’; = ‘Vardö, Persfjord’) • Nordland; Sørfold, Røsvik at the S shore of Sørfolda (= ‘Røsvik’) • Rogaland; Sandnes, Sandnes S of Stavanger (= ‘Sandnes’) • Troms; Balsfjord, Labukt (= ‘Labukt’) • Balsfjord, Fjellfrøsvatnet [Fjellfroskvannet] N of Øverbygd (= ‘Fjellfrøskvann’) • Tromsø (= ‘Tromsø’) • Tromsø, lake Prestvannet on the Tromsøya (= ‘Prestvann, Tromsø’) • Trøndelag; Levanger, Hestøya NW of Alstahaug, southern tip Måkeskjær (= ‘Måkeskjær’).

##### Ecological note.

Habitats not specified. Phenology: Jul.–Sep.

#### Cratyna (Spathobdella) perplexa

Taxon classificationAnimaliaDipteraSciaridae

(Winnertz, 1867)

426744B3-6B89-5AEE-B008-2A028D9FC93E

##### Synonyms.

= *brevicornis* (Tuomikoski, 1957); = dispar (Beling, 1885) [preocc.]; = gregaria (Beling, 1872); = *pilosa* (Rübsaamen, 1894) [preocc.]; = *socialis* (Winnertz, 1871).

##### Literature.

*Faunistics*: Soot-Ryen (1942): 80 [as *Neosciara socialis*]; Menzel et al. (1990): 323 [as Plastosciara (Spathobdella) socialis]. *Taxonomy*: Tuomikoski (1957): 14 [as Plastosciara (Spathobdella) brevicornis]; Tuomikoski (1960): 35, 37 [as Plastosciara (Spathobdella) socialis]; Menzel and Mohrig (2000): 284 [as Cratyna (Spathobdella) perplexa].

##### Localities.

• Norway; without further locality details (= ‘Norwegen’) • Troms; Balsfjord, Fjellfrøsvatnet [Fjellfroskvannet] N of Øverbygd (= ‘Fjellfrøskvann’).

##### Ecological note.

Habitats not specified. Phenology: Jul.

#### Ctenosciara hyalipennis

Taxon classificationAnimaliaDipteraSciaridae

(Meigen, 1804)

C2772248-A618-5D1F-AAE2-46C194CF6C30

##### Synonyms.

= *annulata* (Meigen, 1818); = *autumnalis* (Winnertz, 1867); = electa (Grzegorzek, 1884); = eximia (Winnertz, 1867); = insularis (Frey, 1936); = rufa (Grzegorzek, 1884); = *sordidella* (Zetterstedt, 1851).

##### Literature.

*Faunistics*: Zetterstedt (1851): 3728 [as *Sciara hyalipennis*] and 3729 [as *Sciara sordidella*]; Siebke (1863): 176 [as *Sciara hyalipennis*]; Siebke (1877): 211 [as *Sciara hyalipennis*] and 212 [as *Sciara sordidella*]; Lengersdorf (1926b): 3 [as *Sciara autumnalis*];

Soot-Ryen (1942) 75 [as *Lycoria annulata*]; Tuomikoski (1960): 110; Menzel and Martens (1995): 121; Thunes et al. (2003): 493; Thunes et al. (2004): 72, 85 [all as *Ctenosciara hyalipennis*]. *Taxonomy*: Tuomikoski (1960): 110; Menzel and Mohrig (2000): 295 [both as *Ctenosciara hyalipennis*].

##### Localities.

Norway; without further locality details (= ‘Norwegen’) • Buskerud; Sigdal, Heimseteråsen (= ‘Sigdal’) • Finnmark; Alta, Bojobæskihytta in the Stabbursdalen between Karasjok and Alta (= ‘Bojobæske’) • Alta, Jotkajavre fjellstue on the Finnmarksvidda between Karasjok and Alta (= ‘Jotkajavre’) • Hordaland; Kvam, Geitaknottene naturreservat between Hardangerfjorden and Bjørnafjorden NE of Gjermundshamn (= ‘Kvam, Geitaknottane’) • Nordland; Herøy, Måsvær Island (= ‘Måsvær’) • Oslo; Oslo, Tøyen (= ‘ad Christianiam in Tøien’; = ‘in Töien prope Christianiam’; = ‘in Tøien; = ‘Tøyen, Oslo’) • Oslo, Ryenberg (= ‘monte Ryenbjerg’; = ‘Ryenberg, Oslo’) • Oppland; Dovre, Hjerkinn NW of Folldal in the Gudbrandsdalen (= ‘Hjerkin’) • Lesja, Fogstuen on the Dovrefjell plateau (= ‘Fogstuen’; = ‘Fokstuen’, Dovre’; = ‘in alpe Dovre ad Fokstuen’; = ‘in alpe Dovre’; = ‘Dovre’) • Troms; Nordreisa, woodland and farm Hallen at the E shore of Reisaelva SE of Storslett (= ‘Nordreisa, Hallen’) • Trøndelag; Levanger, Skogn SE of Levanger (= ‘ad diversorium Thyæs in parochia Skogn’; = ‘ad diversorium Thynäs prope Levanger’; = ‘Thynäs’) [= in the accommodation of Thy in Skogn] • Oppdal, Kongsvoll near Kongsvold Fjeldstue in the Drivdalen (= ‘Kongsvold’; = ‘in alpe Dovre ad Kongsvold’; = ‘in alpe Dovre’; = ‘Dovre’).

##### Ecological note.

*Pinus sylvestris* dominated boreal forests with *Betula pubescens* and *Picea abies*; rearing of adults from larvae found in rotten wood of gray alder (*Alnus incana*). Phenology: Jun.–Sep.

#### Ctenosciara lutea

Taxon classificationAnimaliaDipteraSciaridae

(Meigen, 1804)

85AE9822-80F8-528A-9C74-A08D2C9C0B01

##### Literature.

*Faunistics*: Siebke (1877): 215 [as *Sciara lutea*; in part]; Soot-Ryen (1942): 76 [as *Lycoria lutea*]. *Taxonomy*: Menzel et al. (1990): 329; Menzel and Mohrig (2000): 298 [both as *Ctenosciara lutea*].

##### Locality.

• Oppland; Øyer in the Gudbrandsdalen (= ‘in par. [parochia] Øier Gudbrandsdaliæ’; = ‘Øier Gudbrandsdaliæ’; = ‘Øyer’).

##### Ecological note.

Habitat not specified. Phenology: Jul.

#### Dichopygina aculeata

Taxon classificationAnimaliaDipteraSciaridae

Vilkamaa, Hippa & Komarova, 2004

7624EAEC-1FA0-522F-8717-05A7A2F17B57

##### Literature.

*Faunistics*: Leng et al. (2018): 19 [as *Dichopygina aculeata*]. *Taxonomy*: Vilkamaa et al. (2004): 110 [as *Dichopygina aculeata*].

##### Locality.

• Norway; without further locality details (= ‘Norway’) • Møre Og Romsdal; Vestnes, Småøyane SE of Kristisetra, SE of Vestnes (published as ‘Norway’; see faunistic note).

##### Faunistic note.

The first specimens of *Dichopygina aculeata* mentioned in Leng et al. (2018: 19) from ‘Norway’ (without locality details) were collected and identified in our NTI project 2014–2016, based on the following material: Norway • 2 ♂♂; ‘Møre og Romsdal; Vestnes, Småøyane SE of Kristisetra (SE of Vestnes)’; 62.5598N, 06.9944E; 170 m a.s.l.; 22 Aug. 2015; K. Heller leg.; sweep net; bog and deadwood rich carr between road and river (downy birch, grey alder, rowan, juniper, rushes, sedges, mosses, lichens); BFCO; BOLD ID SCINO1252-16 (BAB 421460, bf-sci-00981) and SCINO1253-16 (BAB 421463, bf-sci-00982).

##### Ecological note.

Bog and carr rich in dead wood (downy birch, grey alder, rowan, juniper, rushes, sedges, mosses, lichens). Phenology: Aug.

#### Dichopygina bernhardi

Taxon classificationAnimaliaDipteraSciaridae

Vilkamaa, Hippa & Komarova, 2004

E70EDF75-ACFE-5D6E-8049-6A2DB4E9B4E5

##### Literature.

*Faunistics*: Leng et al. (2018): 19, 23 [as *Dichopygina bernhardi*]. *Taxonomy*: Vilkamaa et al. (2004): 115 [as *Dichopygina bernhardi*].

##### Locality.

• Hedmark; Elverum, Starmoen naturreservat SE of Elverum (= ‘Elverum, S Starmoen’; see faunistic note).

##### Faunistic note.

The first specimen of *Dichopygina bernhardi* mentioned in Leng et al. (2018: 19, 23) was collected and identified in our NTI project 2014–2016, based on the following material: Norway • 1 ♂; ‘Hedmark; Elverum, S of Starmoen – I’; 60.8524N, 11.6951E; 205 m a.s.l.; 1–6 Sep. 2014; K.M. Olsen leg.; yellow pan trap; sand pit; BFCO; BOLD ID SCINO736-15 (BAB 410634, bf-sci-00696).

##### Ecological note.

sand pit with open vegetation. Phenology: Sep.

#### Dichopygina nigrohalteralis

Taxon classificationAnimaliaDipteraSciaridae

(Frey, 1948)

95B7FBB1-A1D3-59D8-B9D9-78EEC61BCAEA

##### Literature.

*Faunistics*: Leng et al. (2018): 19, 23 [as *Dichopygina nigrohalteralis*]. *Taxonomy*: Tuomikoski (1960): 70, 72; Menzel and Mohrig (2000): 259 [both as *Corynoptera nigrohalteralis*]; Vilkamaa et al. (2004): 116; Mohrig et al. (2013): 199 [both as *Dichopygina nigrohalteralis*].

##### Localities.

• Norway; without further locality details (= ‘Norway’) • Buskerud; Kongsberg, Haugplassen in the Rajedalen (published as ‘Norway’; see faunistic note) • Oppland; Sør-Aurdal, SE part of Moldberget naturreservat NW of Nes (published as ‘Norway’; see faunistic note) • Trøndelag; Trondheim, Trondheim, Sommerlystvegen (published as ‘Norway’; see faunistic note).

##### Faunistic note.

The first specimens of *Dichopygina nigrohalteralis* mentioned in Leng et al. (2018: 19, 23) were collected and/or identified in our NTI project 2014–2016, based on the following material: Norway • 1 ♂; ‘Sør-Trondelag; Trondheim, Sommerlystvegen 22’; 63.4049N, 10.3829E; 65 m a.s.l.; 11–25 May 2014; E. Stur and T. Ekrem leg.; Malaise trap; garden with lawn and some larger trees at the top of a wooded hill side; NTNU; BOLD ID GMNWF813-14 • 1 ♂; ‘Buskerud; Kongsberg, Haugplassen’; 59.5340N, 09.5677E; 520 m a.s.l.; 26 Sep. 2013; Malaise trap; NW portion of managed meadow with a lot of *Dactylorhiza sambucina* and *Primula veris*; K.M. Olsen leg.; BFCO; BOLD ID SCINO031-14 (bf-sci-00031, BAB 363266) • 1 ♂; ‘Oppland; Sør-Aurdal, Moldberget E’; 60.6199N, 09.8935E; 308 m a.s.l.; 3 Jun. 2014; K. Heller leg.; sweep net; coniferous forest; BFCO; BOLD ID SCINO192-15 (bf-sci-00193, BAB 374132).

##### Ecological note.

Managed meadows dominated by *Dactylorhiza sambucina* and *Primula veris*; gardens with lawn on wooded hills; coniferous forests. Phenology: May–Jun., Aug.–Sep.

#### Dichopygina ramosa

Taxon classificationAnimaliaDipteraSciaridae

Vilkamaa, Hippa & Komarova, 2004

26654424-3B7F-517C-84EF-2F0E40F23804

##### Literature.

*Faunistics*: Leng et al. (2018): 19 [as *Dichopygina ramosa*]. *Taxonomy*: Vilkamaa et al. (2004): 119 [as *Dichopygina ramosa*].

##### Localities.

• Norway; without further locality details (= ‘Norway’) • Akershus; Nesodden, Blåbærstien in Nesoddtangen (published as ‘Norway’; see faunistic note) • Telemark; Kragerø, pond Frydensborgtjenna in Kragerø (published as ‘Norway’; see faunistic note).

##### Faunistic note.

The first specimens of *Dichopygina ramosa* mentioned in Leng et al. (2018: 19) were identified in our NTI project 2014–2016, based on the following sciarid material: Norway • 1 ♂; ‘Akershus; Nesodden, Blåbærstien’; 59.8523N, 10.6698E; 25 March–7 Jun. 2012; O.J. Lønnve leg.; Malaise trap; residential area; BFCO; BOLD ID SCINO235-15 (bf-sci-00237, BAB 374552) • 1 ♂; ‘Telemark; Kragerø, Frydensborgtjenna’; 58.8748N, 09.3992E; 4 m a.s.l.; 17.08–28.09.2009; S. Olberg and A.E. Laugsand leg.; Malaise trap; pond with enhanced growth of aquatic vegetation (probably eutrophic); BFCO; BOLD ID SCINO497-15 (bf-sci-00500, BAB 393143).

##### Ecological note.

At ponds with rich aquatic vegetation; in settled areas. Phenology: Mar.–Jun., Aug.–Sep.

#### Dolichosciara flavipes

Taxon classificationAnimaliaDipteraSciaridae

(Meigen, 1804)

9142200E-0208-5F67-B67F-4483E4661F04

##### Synonyms.

= flavipes var. nigrithorax (Strobl, 1898); = fugax (Grzegorzek, 1884).

##### Literature.

*Faunistics*: Zetterstedt (1852): 4355 [as *Sciara flavipes*]; Siebke (1877): 214 [as *Sciara flavipes* Panzer; recte Meigen] and 215 [in part as *Sciara lutea*; misidentification]; Soot-Ryen (1942): 75 [as *Phorodonta flavipes*]. *Taxonomy*: Tuomikoski (1960): 108, 109; Mohrig and Menzel (1994): 186; Menzel and Mohrig (2000): 440 [all as Phytosciara (Dolichosciara) flavipes]; Vilkamaa (2000): 48 [as *Dolichosciara flavipes*].

##### Localities.

• Oslo; Oslo, Botanisk hage (= ‘in horto botanico ad Christianiam’; = ‘Botanical Garden, Oslo’) • Oslo, Tøyen (= ‘ad Töien’) • Trøndelag; Oppdal, Kongsvoll near Kongsvold Fjeldstue in the Drivdalen (= ‘ad Kongsvold in alpe Dovre’; = ‘Kongsvold, Dovre’; = ‘Dovre’).

##### Ecological note.

In botanical gardens. Phenology: Aug.–Sep.

#### Epidapus (Epidapus) alnicola

Taxon classificationAnimaliaDipteraSciaridae

(Tuomikoski, 1957)

4CCE1F42-9020-5290-BF77-BD48452771F2

##### Literature.

*Faunistics*: Tuomikoski (1960): 100 [as *Caenosciara alnicola*]; Menzel et al. (1990): 347 [as Caenosciara (Bonessia) alnicola]. *Taxonomy*: Tuomikoski (1957): 16 [as *Vimmeria alnicola*]; Tuomikoski (1960): 100 [as *Caenosciara alnicola*]; Mohrig (1970): 144 [as Caenosciara (Bonessia) alnicola]; Menzel and Mohrig (2000): 319 [as Epidapus (Epidapus) alnicola].

##### Localities.

• Norway; without further locality details (= ‘Norwegen’) • Troms; Nordreisa, woodland and farm Hallen on the E shore of Reisaelva SE of Storslett (= ‘Troms, Hallen’).

##### Ecological note.

From rotten wood of gray alder (*Alnus incana*). Phenology: without data.

#### Epidapus (Epidapus) gracilis

Taxon classificationAnimaliaDipteraSciaridae

(Walker, 1848)

AE176705-CA14-5584-8C08-3EABCDCD10B3

##### Synonyms.

= aptera (Kieffer, 1903); = *edwardsi* Freeman, 1983; = *gracilis* (Winnertz, 1853) [preocc.]; = *longicornis* (Lengersdorf, 1941); = pulicina (Frey, 1952).

##### Literature.

*Faunistics*: Thunes et al. (2004): 72, 85 [as *Epidapus gracilis*]; Köhler et al. (2014): 329 [as Epidapus (Epidapus) gracilis]. *Taxonomy*: Tuomikoski (1960): 97, 98; Mohrig (1969): 54 [both as Epidapus (Epidapus) gracilis (Winnertz)]; Freeman (1983): 170 [as *Epidapus edwardsi*]; Menzel and Mohrig (2000): 319 [as Epidapus (Epidapus) gracilis (Walker)].

##### Localities.

• Buskerud; Sigdal, Heimseteråsen (= ‘Sigdal’) • Vestfold; Larvik, lake Skjærsjø near Kvelde NW of Larvik (= ‘Larvik, Skjærsjø’).

##### Ecological note.

*Pinus sylvestris* dominated boreal forests with *Betula pubescens* and *Picea abies*; oak canopies of *Quercus robur*. Phenology: Jul.

#### Hemineurina abbrevinervis

Taxon classificationAnimaliaDipteraSciaridae

(Holmgren, 1869)

0387BEA1-4047-54FC-93E2-4B2944A34F36

##### Literature.

*Faunistics*: Holmgren (1869): 16, 54; Lengersdorf (1930a): 56 [both as *Sciara abbrevinervis*]; Frey (1948): 35, 85, 91 [as Bradysia (Bradysia) abbrevinervis]; Tuomikoski (1967): 48; Menzel and Mohrig (2000): 402; Coulson and Refseth (2004): 103; Coulson (2008): 161; Coulson (2013): 154 [all as Lycoriella (Hemineurina) abbrevinervis]. *Taxonomy*: Tuomikoski (1967): 48; Menzel and Mohrig (2000): 402 [both as Lycoriella (Hemineurina) abbrevinervis]; Vilkamaa and Menzel (2019): 50 [as *Hemineurina abbrevinervis*].

##### Localities.

• Svalbard; Spitsbergen, Kobbefjorden at the NW coast near the Danskøya (= ‘in Spetsbergia ad Kobbebay’; = ‘Spetsbergia ad Kobbebay’; = ‘Spetsbergia, Kobbebay’; = ‘Spitzbergen bei Kobbefjorden’) • Spitsbergen, without further locality details (= ‘Spetsbergen’; = ‘Spitsbergen’; = ‘Spitzbergen’).

##### Ecological note.

Bird cliffs. Phenology: Jul.

#### Hemineurina conspicua

Taxon classificationAnimaliaDipteraSciaridae

(Winnertz, 1867)

6BEF7EA0-A469-5A46-AF30-4BD50C70DD9F

##### Synonym.

= polychaeta (Pettey, 1918)

##### Literature.

*Faunistics*: Lengersdorf (1926b): 4 [as *Sciara conspicua*]; Soot-Ryen (1942): 77 [as *Neosciara conspicua*]; Menzel et al. (1990): 335 [as Lycoriella (Hemineurina) conspicua]. *Taxonomy*: Tuomikoski (1960): 75, 76; Menzel and Mohrig (2000): 400; Mohrig et al. (2013): 210 [all as Lycoriella (Hemineurina) conspicua]; Vilkamaa and Menzel (2019): 50 [as *Hemineurina conspicua*].

##### Localities.

• Norway; without further locality details (= ‘Norwegen’) • Hordaland; Modalen, Mo (= ‘Mo’) • Oslo; Oslo, Tøyen (= ‘Tøien’; = ‘Tøyen’) • Troms; Tromsø (= ‘Tromsøy’).

##### Ecological note.

Habitats not specified. Phenology: Aug.–Sep.

#### Hemineurina inflata

Taxon classificationAnimaliaDipteraSciaridae

(Winnertz, 1867)

8AC5BE28-4677-5A40-9652-C019BFB7594D

##### Synonyms.

= difficilis (Grzegorzek, 1884); = interdicta (Grzegorzek, 1884); = nitens (Winnertz, 1867); = subvenosa (Mohrig & Krivosheina, 1983).

##### Literature.

*Faunistics*: Zetterstedt (1851): 3758; Siebke (1877): 214 [both as *Sciara venosa*; misidentification]; Lengersdorf (1926b): 3 [as *Sciara inflata*]; Soot-Ryen (1942): 80 [in part as *Neosciara vittigera*; misidentification (only cited *inflata* specimens)]; Tuomikoski (1960): 77 [as Lycoriella (Hemineurina) venosa sensu Frey; misidentification]. *Taxonomy*: Tuomikoski (1960): 75, 77 [as Lycoriella (Hemineurina) venosa sensu Frey; misidentification]; Menzel and Mohrig (2000): 403 [as Lycoriella (Hemineurina) inflata]; Vilkamaa and Menzel (2019): 50 [as *Hemineurina inflata*].

##### Localities.

• Nordland; Sømna, Sømnes at the bay Sømnesvika N of Vik (= ‘Sømnes’) • Sørfold, Røsvik at the S shore of Sørfolda (= ‘Røsvik’) • Oslo; Oslo, Tøyen (= ‘in Töien prope Christianiam’; = ‘ad Christianiam in Tøien’; = ‘Tøyen, Oslo’) • Troms; Balsfjord/Målselv/Tromsø [former municipality ‘Malangen’] (= ‘Malangen’) • Tromsø (= ‘Tromsø’) • Tromsø, Ramfjorden (= ‘Ramfjord’) • Trøndelag; Verdal, former poststation ‘Suulstuen’ SE of Vuku at the Jamtlandsvegen [road no. 72] (= ‘in jugo alpino Norvegiæ ad Suulstuen’; = ‘ad Suulstuen Værdaliæ’; = ‘ad Suul Værdaliæ’; = ‘Sulstuen, Værdal’; = ‘ad Suul’).

##### Ecological note.

On mountains. Phenology: Jun.–Aug.

#### Hemineurina modesta

Taxon classificationAnimaliaDipteraSciaridae

(Staeger, 1840)

AB78887A-63D9-5752-B904-CA6BFCA9777A

##### Synonyms.

= *arctica* (Holmgren, 1869); = conglomerata (Pettey, 1918); = *ecalcarata* (Holmgren, 1869); = *frigida* (Holmgren, 1869) [preocc.]; = fumatella (Lundbeck, 1898); = *globiceps* (Becher, 1886); = *groenlandica* (Holmgren, 1872); = *holmgreni* (Rübsaamen, 1894).

##### Literature.

*Faunistics*: Holmgren (1869): 16, 52 [as *Sciara arctica* and *Sciara ecalcarata*] and 15, 53 [as *Sciara frigida*]; Becher (1886): 62; Edwards (1923): 236 [both as *Sciara globiceps*]; Edwards (1925): 354; Summerhayes and Elton (1928): 209, 220, 221, 225 [both as *Sciara holmgreni*]; Lengersdorf (1930a): 55 [as *Sciara arctica* and *Sciara frigida*]; Lengersdorf (1930c): 52 [as *Sciara groenlandica*]; Edwards (1935): 533; Bertram and Lack (1938): 51 [both as *Sciara holmgreni*]; Frey (1948): 91 [as Bradysia (Hemineurina) modesta var. frigida]; Tuomikoski (1967): 48 [as Lycoriella (Hemineurina) modesta], 49 [as *Sciara acrctica*] and 50 [as *Sciara ecalcarata*]; Menzel et al. (1990): 337 [as Lycoriella (Hemineurina) modesta]; Menzel and Mohrig (2000): 405 [as *Sciara acrctica*, *Sciara ecalcarata* and *Sciara frigida* under Lycoriella (Hemineurina) modesta] and 198 [in the discussion of *Camptochaeta delicata*; misidentification]; Coulson and Refseth (2004): 103 [as Lycoriella (Hemineurina) globiceps and Lycoriella (Hemineurina) modesta]; Thunes et al. (2004): 85 [as *Lycoriella globiceps*]; Coulson (2008): 161; Coulson (2013): 154 [both as Lycoriella (Hemineurina) modesta]; ? Coulson et al. (2013): 6 [as Lycoriella (Hemineurina) sp.]. *Taxonomy*: Tuomikoski (1960): 75, 77; Menzel and Mohrig (2000): 405; Mohrig et al. (2013) 213 [all as Lycoriella (Hemineurina) modesta]; Vilkamaa and Menzel (2019): 10, 51 [as *Hemineurina modesta*].

##### Localities.

• Norway; without further locality details (= ‘Norwegen’) • Buskerud; Sigdal, Heimseteråsen (= ‘Sigdal’).

• Jan Mayen: without further locality details (= ‘Jan Mayen’; = ‘Jan Mayen Island’) • Svalbard; Bjørnøya (= ‘Bear Island’) • Bjørnøya, bay Austervåg at the E coast (= ‘bei Austervåg (B.)’) • Bjørnøya, mining camp Tunheim at the NE coast (= ‘Bear Island, Tunheim’) • Bjørnøya, Røyevatnet in the SW part of island (= ‘Bear Island, Röyevatnet’) • Spitsbergen, Amsterdamøya, Smeerenburg at the SE coast (= ‘in Spetsbergia ad Smeerenberg’; = ‘Spitzbergen, Smeerenberg’) • Spitsbergen, Bellsund at the W coast (= ‘in Spetsbergia ad Belsund’) • Spitsbergen, Edgeøya at the Storfjorden, ? Kvalpynten at the N side of the mouth of Tjuvfjorden (= ‘in Spetsbergia ad Whales Point in Storfjorden’) • Spitsbergen, Grønfjorden (= ‘in Spetsbergia ad Green Harbour’; = ‘Spetsbergia, Green Harbour’; = ‘Spitzbergen, Green Harbour’; = ‘Grønfjorden’) • Spitsbergen, Grønfjorden, Barentsburg (= ‘Barentsburg’; = ‘bei Barentsburg (S.)’; = ‘Grønfjord, Barentsburg’) • Spitsbergen, Haakon VII Land, Gerdøya in Dyrevika at the head of Kongsfjorden (= ‘Head of King’s Bay, Deer Bay Island’) • Spitsbergen, Haakon VII Land, Reinsdyrflya, at the Liefdefjorden (= ‘Spitsbergen, Reindeer Peninsula, at the Liefde Bay’) • Spitsbergen, Hiorthhamn [former mining settlement] at the E side of Adventfjorden (= ‘Hjorthhamn’; = ‘Hiorthhamn (S.), bei Residensen’) • Spitsbergen, Kobbefjorden at the NW coast near the Danskøya (= ‘in Spetsbergia ad Kobbebay’; = ‘in Spetsbergia ad Kobbebay [Spitzbergen, bei Kobbefjorden]’; = ‘Kobbebay’; = ‘Kobbefjorden’) • Spitsbergen, Nordaustlandet (= ‘Spitsbergen, North-East Land’) • Spitsbergen, Nordenskiöld Land, Mälardalen at the N side of the mouth of Adventelva (= ‘Mælardalen’; = ‘Maelardalen [Mælardalen]’) • Spitsbergen, without further locality details (= ‘Spetsbergen; = ‘Spitzbergen’; = ‘Spitsbergen’).

##### Ecological note.

Lichen-moss heath; bogs (grass-swamp); mats of *Luzula confusa* and mosses; in hollows and slight crevices of erratic boulders with mosses and lichens; plant community ‘fjaeldmark’ (= feldmark; mountain field) with phanerogams, mosses, lichens and *Salix polaris*; in mosses and lichens; on grass-leaves, moss-hummocks and hard snowdrifts near streams; among stones; from plants in stony areas and on large boulders; mixture of discarded greenhouse soil and manure from animal houses (all Svalbard records); *Pinus sylvestris* dominated boreal forests with *Betula pubescens* and *Picea abies*. Phenology: Jun.–Sep.

#### Hemineurina postconspicua

Taxon classificationAnimaliaDipteraSciaridae

(Mohrig, 1985)

DF41F019-3CEA-5ED6-BE5F-E144D18FC6B5

##### Literature.

*Faunistics*: Hågvar et al. (2007): 67; Coulson (2008): 161; Coulson (2013): 154 [all as *Lycoriella postconspicua*]. *Taxonomy*: Mohrig (1985): 236 [as *Lycoriella postconspicua*]; Menzel and Mohrig (2000): 385 [as Lycoriella (Hemineurina) postconspicua]; Vilkamaa and Menzel (2019): 51 [as *Hemineurina postconspicua*].

##### Localities.

• Svalbard; Spitsbergen, Kapp Linné by the Isfjord, Isfjord Radio station (= ‘Svalbard, Isfjord Radio’) • Spitsbergen, Ny-Ålesund.

##### Ecological note.

On dry ridges and slopes, with *Saxifraga oppositifolia*, mosses and lichens; in the ground vegetation with *Poa* spec., *Oxyria digyna* and *Deschampsia cespitosa*. Phenology: Jun.–Jul.

#### Hemineurina venosa

Taxon classificationAnimaliaDipteraSciaridae

(Staeger, 1840)

CB2D4421-B676-5A90-B65A-A1A2DB7F61E8

##### Synonyms.

= crassivenosa (Lengersdorf, 1943); = *lepida* (Winnertz, 1867); = *praevenosa* (Mohrig & Menzel, 1990).

##### Literature.

*Faunistics*: Lengersdorf (1926b): 3 [as *Sciara lepida*]; Soot-Ryen (1942): 78 [as *Neosciara lepida*]. *Taxonomy*: Menzel et al. (1990): 337 [as Lycoriella (Hemineurina) praevenosa]; Menzel and Mohrig (2000): 407 [as Lycoriella (Hemineurina) venosa]; Vilkamaa and Menzel (2019): 51 [as *Hemineurina venosa*].

##### Locality.

• Troms; Målselv, farm Frihetsli in the Dividalen 32 km SE of Øverbygd (= ‘Frihetsli’).

##### Ecological note.

Habitat not specified. Phenology: Jul.–Aug.

#### Leptosciarella (Hirtipennia) hirtipennis

Taxon classificationAnimaliaDipteraSciaridae

(Zetterstedt, 1838)

74DA4162-0D8A-52B7-9868-CEE11DCD36C3

##### Synonyms.

= absurda (Winnertz, 1867); = hirtipennis var. minor (Frey, 1948); = jugicola (Strobl, 1898); = parcepilosa var. opacicollis (Strobl, 1902).

##### Literature.

*Faunistics*: Zetterstedt (1838): 826; Zetterstedt (1851): 3731; Siebke (1877): 212 [all as *Sciara hirtipennis*]; Lengersdorf (1926b): 9; Soot-Ryen (1942): 75 [both as *Trichosia hirtipennis*]; Menzel et al. (1990): 316 [as Trichosia (Leptosciarella) hirtipennis]. *Taxonomy*: Tuomikoski (1960): 20, 21 [as Trichosia (Leptosciarella) hirtipennis]; Mohrig and Menzel (1997): 45; Menzel and Mohrig (2000): 369 [both as Leptosciarella (Hirtipennia) hirtipennis].

##### Localities.

• Norway; without further locality details (= ‘Nord-Norwegen’) • Nordland; Narvik, Bjerkvik at the Ofotfjorden NE of Narvik (= ‘in Nordlandia ad diversorium Bjørkvik; = ‘ad diversorium Björkvik juxla Ofodenfjoid’; = ‘ad Bjŏrkvik Nordlandiae’; = ‘Bjørkvik, Ofoten’; = ‘Lappland (Norwegen)’ [misinterpretation in Menzel et al. (1990), correctly ‘Nordland (Norwegen)’]).

##### Ecological note.

Habitats not specified. Phenology: Jul.

#### Leptosciarella (Leptosciarella) fuscipalpa

Taxon classificationAnimaliaDipteraSciaridae

(Mohrig & Mamaev, 1979)

A2883F45-8828-5DE0-AF23-DF41B88F23BC

##### Literature.

*Faunistics*: Mohrig and Menzel (1997): 65; Komarova (2016a): 197; Komarova (2016b): 256 [all as Leptosciarella (Leptosciarella) fuscipalpa]. *Taxonomy*: Mohrig and Menzel (1997): 65; Menzel and Mohrig (2000): 360 [both as Leptosciarella (Leptosciarella) fuscipalpa].

##### Localities.

• Norway; without further locality details (= ‘Norwegia’; = ‘Norway’) • Finnmark; Berlevåg/Nesseby/Tana/Vadsø/Vardø, Varangerhalvøya (= ‘Finmark, Waranger-Halbinsel’) • Østfold; Hvaler, Hvaløerne (= ‘Hvaløerne’).

##### Ecological note.

Habitats not specified. Phenology: Jul.

#### Leptosciarella (Leptosciarella) hispida

Taxon classificationAnimaliaDipteraSciaridae

(Winnertz, 1867)

2694D486-DDF9-5A8A-AEB6-7AE0794F1079

##### Literature.

*Faunistics*: Zetterstedt (1871): 3721 [as *Sciara trochanterata*; in part misidentification]; Lengersdorf (1926b): 3 [as *Sciara hispida*]; Lengersdorf (1930a): 49; Lengersdorf (1941): 48 [both as *Sciara hispida* under *Sciara trochanterata*; misidentification]; Soot-Ryen (1942): 76 [in part as *Lycoria trochanterata*; misidentification (only cited specimen from ‘Festningsstuen’)]. *Taxonomy*: Lengersdorf (1941): 48 [as *Sciara hispida*]; Mohrig and Menzel (1997): 63; Menzel and Mohrig (2000): 366 [both as *Sciara hispida* under Leptosciarella (Leptosciarella) rejecta; misidentification].

##### Localities.

• Finnmark; Porsanger, farm Fæstningsstua near Lævnasjarvi W of Skoganvarre (= ‘Fæstningstuen’; = ‘Festningsstuen’) • Oslo; Oslo, Tøyen (= ‘in Töien prope Christianiam’; = ‘Toiën’; = ‘Toien’) • Trøndelag; Meråker, NE of mountain Kølhaugan near the Swedish border [maybe a collecting place in Sweden: Jämtland, village Skalstugan close to the border with Norway] (= ‘in Jemtlandia ad diversorium Skalstugan’; = ‘Skalstuga’).

##### Ecological note.

Habitats not specified. Phenology: Jun.–Aug.

#### Leptosciarella (Leptosciarella) nudinervis

Taxon classificationAnimaliaDipteraSciaridae

(Tuomikoski, 1960)

6B668EED-D502-55C8-9329-EAF25E66D939

##### Literature.

*Faunistics*: Mohrig and Menzel (1997): 81 [as Leptosciarella (Leptosciarella) nudinervis]. *Taxonomy*: Tuomikoski (1960): 21, 25 [as Trichosia (Leptosciarella) nudinervis]; Mohrig and Menzel (1997): 81; Menzel and Mohrig (2000): 365 [both as Leptosciarella (Leptosciarella) nudinervis].

##### Locality.

• Finnmark; Båtsfjord, Varangerhalvøya, Syltefjorden (= ‘Varranger-Halbinsel, Sylkefjord’).

##### Ecological note.

Habitats not specified. Phenology: Jul.

#### Leptosciarella (Leptosciarella) pilosa

Taxon classificationAnimaliaDipteraSciaridae

(Staeger, 1840)

F260F2DB-4BB6-55D4-9B86-31A73858189D

##### Literature.

*Faunistics*: Siebke (1863): 176; Siebke (1877): 217; Lengersdorf (1926b): 3 [all as *Sciara pilosa*]; Soot-Ryen (1942): 76 [as *Lycoria pilosa*]; Menzel et al. (1990): 314 [as Trichosia (Leptosciarella) pilosa]; Komarova (2016a): 197; Komarova (2016b): 256 [both as Leptosciarella (Leptosciarella) pilosa]. *Taxonomy*: Tuomikoski (1960): 21, 25 [as Trichosia (Leptosciarella) scutellata sensu Frey; misidentification]; Mohrig and Menzel (1997): 72; Menzel and Mohrig (2000): 365 [both as Leptosciarella (Leptosciarella) pilosa].

##### Localities.

• Norway; without further locality details (= ‘Norwegia’; = ‘Norway’; = ‘Norwegen’) • Finnmark; Alta, Jotkajavre fjellstue on the Finnmarksvidda between Karasjok and Alta (= ‘Jotkajavre’) • Trøndelag; Oppdal, Kongsvoll near Kongsvold Fjeldstue in the Drivdalen (= ‘ad Kongsvold in alpe Dovre’; = ‘Kongsvold’; = ‘in alpe Dovre’) • Troms; Balsfjord, Fjellfrøsvatnet [Fjellfroskvannet] N of Øverbygd (= ‘Fjellfrøskvann’) • Balsfjord, Øverbygd (= ‘Øverbygd’).

##### Ecological note.

Habitats not specified. Phenology: Jul.

#### Leptosciarella (Leptosciarella) scutellata

Taxon classificationAnimaliaDipteraSciaridae

(Staeger, 1840)

F8D73E41-3CE2-52E2-BC63-EC46A51A5CEC

##### Synonyms.

= *bilineata* (Staeger, 1840); = *elegans* (Winnertz, 1867); = inhonesta (Winnertz, 1867); = interrupta (Strobl, 1895); = obscuripennis (Winnertz, 1867).

##### Literature.

*Faunistics*: Siebke (1877): 210 [as *Sciara bilineata*]; Soot-Ryen (1942): 76 [as *Lycoria scutellata*]. *Taxonomy*: Tuomikoski (1960): 21 [as Trichosia (Leptosciarella) elegans]; Mohrig and Menzel (1997): 58; Menzel and Mohrig (2000): 361 [both as Leptosciarella (Leptosciarella) scutellata].

##### Locality.

• Oslo; Oslo, Bekkelaget (= ‘Bækkelaget ad Christianiam’; = ‘Bekkelaget’).

##### Ecological note.

Habitat not specified. Phenology: May.

#### Leptosciarella (Leptosciarella) trochanterata

Taxon classificationAnimaliaDipteraSciaridae

(Zetterstedt, 1851)

F7E5ED8A-D2AD-59C2-BD32-5908071E33F3

##### Synonyms.

= *coarctata* (Winnertz, 1867); = hirsutissima (Strobl, 1895); = prisca (Winnertz, 1867); = saltuum (Winnertz, 1868); = *splendens* (Winnertz, 1867) [*Sciara*].

##### Literature.

*Faunistics*: Zetterstedt (1871): 3721; Siebke (1877): 211 [both as *Sciara trochanterata*; in part]; Soot-Ryen (1942): 76 [as *Lycoria trochanterata*; in part]; Menzel et al. (1990): 314 [as Trichosia (Trichosia) trochanterata; in part]; Mohrig and Menzel (1997): 54; Menzel and Mohrig (2000): 367 [both as Leptosciarella (Leptosciarella) trochanterata]. *Taxonomy*: Tuomikoski (1960): 21, 24 [as Trichosia (Leptosciarella) coarctata]; Mohrig and Menzel (1997): 54; Menzel and Mohrig (2000): 367 [both as Leptosciarella (Leptosciarella) trochanterata].

##### Localities.

• Oslo; Oslo, Botanisk hage (= ‘in horto botanico ad Christianiam’) • Oslo, Tøyen (= ‘in Töien prope Christianiam’; = ‘Töien nahe Kristiania [Oslo]’; = ‘Töien [Oslo]’; = ‘Tøien, Oslo’) • Trøndelag; Verdal, near Sul, between Kongsstuggu [formerly ‘Kongsstuen fjeldstue’] and Høyfjellsbro (= ‘inter Kongsstuen et Höjfjeldbroe’; = ‘Kongstuen und Höjfjeldroe’; = ‘Höjfjeldbroe’; = ‘between Kongsstuen and Høifjellsbro’).

##### Ecological note.

On mountains; in botanical gardens. Phenology: Jun.–Jul.

#### Leptosciarella (Leptosciarella) truncata

Taxon classificationAnimaliaDipteraSciaridae

(Tuomikoski, 1960)

FE81D90F-3D25-5E14-BEB0-B16F8BB7E5E8

##### Literature.

*Faunistics*: Tuomikoski (1960): 27 [as Trichosia (Leptosciarella) truncata]; Mohrig and Menzel (1997): 80; Menzel and Mohrig (2000): 368; Komarova (2016a): 198; Komarova (2016b): 258 [all as Leptosciarella (Leptosciarella) truncata]. *Taxonomy*: Tuomikoski (1960): 21, 27 [as Trichosia (Leptosciarella) truncata]; Mohrig and Menzel (1997): 80; Menzel and Mohrig (2000): 368 [both as Leptosciarella (Leptosciarella) truncata].

##### Localities.

• Norway; without further locality details (= ‘Norwegia’; = ‘Norway’) • Finnmark; Båtsfjord, Varangerhalvøya, Syltefjorden (= ‘Varranger-Halbinsel, Syltefjord’) • Tana, upper part of the Langfjordelva E of the Porsangerfjorden (= ‘Finmark, Langfjordelva’ [= ‘Finmark, am oberen Lauf des Flusses Langfjordelva östlich vom Porsangerfjord’; = ‘Oberlauf des Flusses Langfjordelva östlich vom Porsangerfjord’]).

##### Ecological note.

Habitats not specified. Phenology: Jun., Aug.

#### Lycoriella brevipila

Taxon classificationAnimaliaDipteraSciaridae

Tuomikoski, 1960

24600B91-089E-5EAC-B734-DBDD544CC1F9

##### Literature.

*Faunistics*: Tuomikoski (1960): 82 [as Lycoriella (Lycoriella) brevipila]. *Taxonomy*: Tuomikoski (1960): 79, 82 [as Lycoriella (Lycoriella) brevipila]; Menzel and Mohrig (2000): 393 [as Lycoriella (Lycoriella) brevipila under Lycoriella (Lycoriella) ingenua; misidentification]; Menzel and Heller (2007): 220 [as Lycoriella (Lycoriella) brevipila]; Vilkamaa and Menzel (2019): 51 [as *Lycoriella brevipila*].

##### Locality.

• Troms; Nordreisa, Sappen (= ‘Sappen’).

##### Ecological note.

Habitat not specified. Phenology: Aug.

#### Lycoriella ingenua

Taxon classificationAnimaliaDipteraSciaridae

(Dufour, 1839)

C4318167-BB9B-57E4-B816-B43C976B7125

##### Synonyms.

= caesar (Johannsen, 1929); = bigoti (Laboulbène, 1863); = celer (Winnertz, 1867); = debilis (Winnertz, 1867); = decliva (Winnertz, 1867); = flammulinae (Sasakawa, 1983); = flaviventris (Winnertz, 1867); = humilis (Winnertz, 1867); = jauva (Rapp, 1946); = mali (Fitch, 1856); = mycorum (Frey, 1948); = pauciseta (Felt, 1897); = pleuroti Yang & Zhang, 1987; = ramicola (Kieffer, 1919), = segnis (Winnertz, 1871); = *solani* (Winnertz, 1871); = velox (Winnertz, 1867); = venusta (Winnertz, 1867); = womersleyi (Séguy, 1940).

##### Literature.

*Faunistics*: Siebke (1863): 177 [as *Sciara fenestralis*; misidentification]; Siebke (1877): 214 [as *Sciara fenestralis*; misidentification] and 215 [as *Sciara pectoralis*; misidentification]; Lengersdorf (1930a): 51 [as *Sciara solani* under *Sciara sordidella*; misidentification]; Soot-Ryen (1942): 77 [as *Neosciara fenestralis*; misidentification]; Kjœrandsen (1993): 155 [as Lycoriella cf. solani]; Komarov (2009): 102; Menzel and Müller (2011): 164 [both as Lycoriella (Lycoriella) castanescens]; Menzel et al. (2013): 291 [as Lycoriella (Lycoriella) ingenua]; Østbye and Lauritzen (2013): 46, 48 [as Lycoriella cf. solani]; Köhler et al. (2014): 329 [as Lycoriella (Lycoriella) ingenua]. *Taxonomy*: Tuomikoski (1960): 79, 84 [as Lycoriella (Lycoriella) solani]; Menzel and Mohrig (2000): 393; Menzel et al. (2013): 291; Mohrig et al. (2013): 211 [all as Lycoriella (Lycoriella) ingenua]; Broadley et al. (2018): 215; Vilkamaa and Menzel (2019): 52 [both as *Lycoriella ingenua*].

##### Localities.

• Norway; without further locality details (= ‘Norwegen’; = ‘Norway’; = ‘Norwegia’; = ‘almindelig overalt’ [ordinary everywhere]) • Akershus: Eidsvoll, Minnesund (= ‘ad Eidsvold’; = ‘ad Eidsvoll’; = ‘Eidsvoll’) • Hordaland; Bergen, Gymmeland (= ‘Bergen, Gymmeland, GR [gruve] 1:50M’) • Osterøy, Nonås mine filed (= ‘Osterøy, Nonås, gruve 1’) • Nordland; Øksnes, in the NW part of Langøya of the Vesterålen archipelago (= ‘Øksnes’) • Oppland; Dovre, Hjerkinn NW of Folldal in the Gudbrandsdalen (= ‘in alpe Dovre, ad Jerkin’; = ‘Hjerkin, Dovre’) • Lesja, Fogstuen on the Dovrefjell plateau (= ‘in alpe Dovre ad Fokstuen’; = ‘Dovre ad Fogstuen’; = ‘Fokstuen, Dovre’) • Oslo; Oslo, Botanisk hage (= ‘Botanical Garden, Oslo’) • Oslo, Tøyen (= ‘ad Christianiam in Tøien’; = ‘Tøyen, Oslo’) • Telemark; Drangedal, Djupedal 1.5 km SE of Henneseid (= ‘Drangedal, Djupedal, Henseid’) • Troms; Balsfjord, Fjellfrøsvatnet [Fjellfroskvannet] N of Øverbygd (= ‘Fjellfrøskvann’) • Karlsøy, Finnkroken at the SW tip of Reinøya (= ‘Finnkroken’) • Tromsø (= ‘Tromsø’) • Tromsø, Ramfjorden (= ‘Ramfjord’) • Trøndelag; Levanger, Skogn SE of Levanger (= ‘Thynas’; = ‘Tynes’) [= in the accommodation of Thy in Skogn] • Oppdal, Kongsvoll near Kongsvold Fjeldstue in the Drivdalen (= ‘in alpe Dovre ad Kongsvold’; = ‘Kongsvold, Dovre’; = ‘ad Kongsvold’).

##### Ecological note.

Oak canopies of *Quercus robur*; in houses; as well as in caves and mines. Phenology: Jun.–Sep.; Mar. and Jul. in caves and mines.

#### Lycoriella latilobata

Taxon classificationAnimaliaDipteraSciaridae

Menzel & Mohrig, 2000

F6F1F570-0301-53B8-97B8-6C4275028CCD

##### Literature.

*Faunistics*: Thunes et al. (2004): 85 [as *Lycoriella latilobata*]. *Taxonomy*: Tuomikoski (1960): 79, 86 [as Lycoriella (Lycoriella) obscuratipes; misidentification]; Menzel and Mohrig (2000): 396 [as Lycoriella (Lycoriella) latilobata]; Vilkamaa and Menzel (2019): 52 [as *Lycoriella latilobata*].

##### Locality.

• Buskerud; Sigdal, Heimseteråsen (= ‘Sigdal’).

##### Ecological note.

*Pinus sylvestris* dominated boreal forests with *Betula pubescens* and *Picea abies*. Phenology: Jun.–Jul.

#### Lycoriella parva

Taxon classificationAnimaliaDipteraSciaridae

(Holmgren, 1869)

71F4BB5D-A8AA-58E7-9A7C-41334F24BFF6

##### Synonyms.

= *curvispina* Tuomikoski, 1960; = difficilis var. obscuratipes (Frey, 1948).

##### Literature.

*Faunistics*: Holmgren (1869): 16, 52; Lengersdorf (1930a): 56; Edwards (1935): 535; Bertram and Lack (1938): 51 [all as *Sciara parva*]; Frey (1948): 35, 85 [as Bradysia (Bradysia) parva]; Tuomikoski (1967): 49; Menzel and Mohrig (2000): 398; Coulson and Refseth (2004): 103; Coulson (2008): 162; Coulson (2013): 154; Mohrig et al. (2013): 271 [all as Lycoriella (Lycoriella) parva]. *Taxonomy*: Tuomikoski (1960): 79, 85 [as Lycoriella (Lycoriella) curvispina]; Tuomikoski (1967): 49; Menzel and Mohrig (2000): 398; Mohrig et al. (2013): 271 [all as Lycoriella (Lycoriella) parva]; Vilkamaa and Menzel (2019): 52 [as *Lycoriella parva*].

##### Localities.

• Svalbard; Bjørnøya (= ‘Bear Island’) • Bjørnøya, Laksvatnet in the N part of island (= ‘Bear Island, Laksvatnet’) • Spitsbergen, Kobbefjorden at the NW coast near the Danskøya (= ‘in Spetsbergia ad Kobbebay’; = ‘Spitzbergen bei Kobbefjorden’) • Spitsbergen, without further locality details (= ‘Spitzbergen’; = ‘Spitsbergen’).

##### Ecological note.

Habitats not specified. Phenology: Jul.–Aug.

#### Lycoriella piristylata

Taxon classificationAnimaliaDipteraSciaridae

Vilkamaa, Hippa & Heller, 2013

4FBD417B-136F-558D-9B6E-8F7AFEF493E6

##### Literature.

*Faunistics*: Vilkamaa et al. (2013c): 52 [as Lycoriella (Hemineurina) piristylata]; Vilkamaa and Menzel (2019): 12 [as *Lycoriella piristylata*]. *Taxonomy*: Vilkamaa and Menzel (2019): 12, 52 [as *Lycoriella piristylata*].

##### Locality.

• Finnmark; Båtsfjord, Varangerhalvøya, Ytre Syltefjord 35 km SE of Båtsfjord (= ‘Varanger Peninsula, Ytre Syltefjord 35 km SE Batsfjord’; = ‘Norway’).

##### Ecological note.

Dwarf-shrub tundra. Phenology: Jul.

#### Lycoriella sativae

Taxon classificationAnimaliaDipteraSciaridae

(Johannsen, 1912)

F9E5E4B3-FA7E-5371-B549-B8ED45C49452

##### Synonyms.

= agarici Loudon, 1978; = auberti (Séguy, 1940); = brevipetiolata (Shaw, 1941); = *castanescens* (Lengersdorf, 1940); = difficilis (Frey, 1948) [preocc.]; = *fucorum* (Frey, 1948); = jeanneli (Séguy, 1940); = kaiseri (Shaw, 1941); = paucisetulosa (Frey, 1948); = rufotincta Tuomikoski, 1959; = similans (Johannsen, 1925); = solispina (Hardy, 1956); = trifolii (Pettey, 1918).

##### Literature.

*Faunistics*: Soot-Ryen (1942): 77 [as *Neosciara auripila*; misidentification]; Tuomikoski (1960): 88; Menzel et al. (1990): 342 [both as Lycoriella (Lycoriella) fucorum]; Menzel and Müller (2011): 164 [as Lycoriella (Lycoriella) castanescens]; Menzel et al. (2013): 292 [as Lycoriella (Lycoriella) sativae]. *Taxonomy*: Tuomikoski (1960): 82, 88 [as Lycoriella (Lycoriella) fucorum]; Menzel and Mohrig (2000): 386 [as Lycoriella (Lycoriella) castanescens]; Menzel et al. (2013): 292; Mohrig et al. (2013): 216 [both as Lycoriella (Lycoriella) sativae]; Broadley et al. (2018): 216; Vilkamaa and Menzel (2019): 52 [both as *Lycoriella sativae*].

##### Localities.

• Norway; without further locality details (= ‘Norwegen’; = ‘Norway’) • Finnmark; Porsanger, two localities on the Porsangerfjorden (= ‘2 Stellen am Porsangerfjord’) • Troms; Tromsø (= ‘Tromsø’) • Trøndelag; Levanger, Hestøya NW of Alstahaug, southern tip Måkeskjær (= ‘Måkeskjær’).

##### Ecological note.

In accumulations of seaweed on sea shores. Phenology: Aug.

#### Pseudolycoriella paludum

Taxon classificationAnimaliaDipteraSciaridae

(Frey, 1948)

3A112957-4C9D-5555-B836-95D1301A9E96

##### Synonyms.

= *leucocera* (Mohrig & Menzel, 1990); = *polliciformis* (Freeman, 1990).

##### Literature.

*Faunistics*: Köhler et al. (2014): 329 [as *Pseudolycoriella paludum*]. *Taxonomy*: Tuomikoski (1960): 44, 47 [as *Corynoptera paludum*]; Menzel et al. (1990): 336 [as Lycoriella (Hemineurina) leucocera]; Freeman (1990): 54 [as *Corynoptera polliciformis*]; Menzel and Mohrig (1998): 369; Menzel and Mohrig (2000): 474 [both as *Pseudolycoriella paludum*].

##### Locality.

• Telemark; Drangedal, Djupedal 1.5 km SE of Henneseid (= ‘Drangedal, Djupedal, Henseid’).

##### Faunistic note.

The first specimens of *Pseudolycoriella paludum* from Norway were identified in our NTI project 2014–2016.

##### Ecological note.

Oak canopies of *Quercus robur*. Phenology: Jul.

#### Scatopsciara (Scatopsciara) atomaria

Taxon classificationAnimaliaDipteraSciaridae

(Zetterstedt, 1851)

2D30A2B3-D5C5-552E-A69B-BFA7575F3D03

##### Synonyms.

= *borealis* (Rübsaamen, 1898); = falsaria (Winnertz, 1867); = hybrida (Winnertz, 1867); = mundula (Winnertz, 1867); = *nacta* (Johannsen, 1912); = pagana (Winnertz, 1867); = pratinicola (Winnertz, 1867); = radialis (Shaw, 1934); = silvestris (Frey, 1936); = soluta (Winnertz, 1867); = *vivida* (Winnertz, 1867).

##### Literature.

*Faunistics*: Zetterstedt (1851): 3761; Siebke (1877): 214 [both as *Sciara atomaria*]; Lengersdorf (1926b): 4 [as *Sciara vivida*]; Lengersdorf (1930c): 52 [as *Sciara borealis* Rübsamer; recte Rübsaamen]; Soot-Ryen (1942): 76 [as *Neosciara atomaria*], 77 [as *Neosciara borealis*] and 80 [as *Neosciara vivida*]; Menzel et al. (1997): 140 [as *Scatopsciara atomaria*]; Menzel and Mohrig (2000): 494 [as Scatopsciara (Scatopsciara) atomaria]; Thunes et al. (2004): 85 [as *Scatopsciara atomaria*]; Mohrig et al. (2013): 235; Köhler et al. (2014): 329 [both as Scatopsciara (Scatopsciara) atomaria]. *Taxonomy*: Tuomikoski (1960): 151, 153 [as *Scaptosciara vivida*; recte *Scatopsciara*]; Menzel and Mohrig (2000): 494; Mohrig et al. (2013): 235 [both as Scatopsciara (Scatopsciara) atomaria]; Broadley et al. (2018): 234 [as *Scatopsciara atomaria*].

##### Localities.

• Akershus; Frogn, Sønderstøa-Degerud (= ‘Degerud’) • Buskerud; Sigdal, Heimseteråsen (= ‘Sigdal’) • Finnmark; Alta, Bossekop in Alta (= ‘Bosekop’) • Alta, Jotkajavre fjellstue on the Finnmarksvidda between Karasjok and Alta (= ‘Jotkajavre’) • Karasjok, Karasjok at the river Karasjohka (= ‘Karasjok’) • Hordaland; Kvam, ‘Berge landskapsvernområde’ [protected landscape area with the Bergsvatnet] NW of Tørvikbygd (= ‘Kvam, Berge’) • Telemark; Drangedal, woodland Steinknapp SW of Drangedal (= ‘Drangedal, Steinknapp’) • Porsgrunn, Mule Varde SE of Porsgrunn at the Eidangerfjorden (= ‘Porsgrunn, Mule Varde’) • Troms; Tromsø (= ‘Tromsø’) • Tromsø, lake Prestvannet on the Tromsøya (= ‘Prestvann, Tromsø’) • Trøndelag; Levanger, Hestøya NW of Alstahaug, southern tip Måkeskjær (= ‘Måkeskjær’) • Levanger, Skogn SE of Levanger (= ‘ad diversorium Thynäs’; = ‘ad Thyæs in Skogn’; = ‘Thynäs’; = ‘Tynes, Værdal’) [= in the accommodation of Thy in Skogn].

• Svalbard; Bjørnøya, Mosevatnet near Kapp Forsberg (= ‘bei Mosevatnet (B.)’).

##### Taxonomic note.

The syntypes (two females) of *Sciara borealis* Rübsaamen were studied by the senior author and identified as a junior synonym of *Scatopsciara atomaria* (Zetterstedt). More detailed information will be presented in a separate publication about the *Sciara* species described by Rübsaamen (1898).

##### Ecological note.

*Pinus sylvestris* dominated boreal forests with *Betula pubescens* and *Picea abies*; oak canopies of *Quercus robur*. In mosses, lichens and *Salix* plants (Svalbard records). Phenology: Jun.–Oct.

#### Scatopsciara (Scatopsciara) brevicornis

Taxon classificationAnimaliaDipteraSciaridae

(Zetterstedt, 1851)

3073003B-2228-5C07-AE2D-3252338CB3A5

##### Literature.

*Faunistics*: Zetterstedt (1851): 3748; Zetterstedt (1860): 6526; Siebke (1877): 213 [all as *Sciara brevicornis*]; Soot-Ryen (1942): 79 [in part as *Neosciara nitidula*; misidentification (only cited *brevicornis* specimens)]. Menzel et al. (1990): 326 [as *Scatopsciara nacta* sensu Tuomikoski; misidentification]; Menzel and Mohrig (2000): 490 [as Scatopsciara (Scatopsciara) brevicornis]. *Taxonomy*: Tuomikoski (1960): 151, 153 [as *Scaptosciara nacta*; misidentification; recte *Scatopsciara*]; Menzel and Mohrig (2000): 490 [as Scatopsciara (Scatopsciara) brevicornis] and 498 [in part as Scaptosciara (Scatopsciara) nacta sensu Tuomikoski; misidentification]; Mohrig et al. (2013): 236 [as Scatopsciara (Scatopsciara) brevicornis in the taxonomic note of Scatopsciara (Scatopsciara) atomaria].

##### Localities.

• Norway; without further locality details (= ‘Norvegia’; = ‘Norwegen’) • Oslo; Oslo, Tøyen (= ‘Tøyen, Oslo’) • Trøndelag; Levanger, Skogn SE of Levanger (= ‘Tynes, Værdal’) [= in the accommodation of Thy in Skogn] • Trondheim (= ‘ad Throndhjem’; = ‘ad Trondhjem’; = ‘ad Trondhjem [bei Trondheim]’; = ‘Trondheim’).

##### Ecological note.

Habitats not specified. Phenology: Jul.

#### Scatopsciara (Scatopsciara) calamophila

Taxon classificationAnimaliaDipteraSciaridae

Frey, 1948

02DEDFC5-59C9-5489-8036-BAAB813896A5

##### Literature.

*Faunistics*: Köhler et al. (2014): 329 [as Scatopsciara (Scatopsciara) calamophila]. *Taxonomy*: Tuomikoski (1960): 151, 154 [as *Scaptosciara calamophila*; recte *Scatopsciara*]; Menzel and Mohrig (2000): 496 [as Scatopsciara (Scatopsciara) calamophila].

##### Localities.

• Telemark; Drangedal, 300 m SE of Henneseid (= ‘Drangedal, Henseid’) • Drangedal, Djupedal 1.5 km SE of Henneseid (= ‘Drangedal, Djupedal, Henseid’) • Drangedal, woodland Steinknapp SW of Drangedal (= ‘Drangedal, Steinknapp’) • Porsgrunn, Mule Varde SE of Porsgrunn at the Eidangerfjorden (= ‘Porsgrunn, Mule Varde’).

##### Faunistic note.

The first specimens of *Scatopsciara calamophila* from Norway were identified in our NTI project 2014–2016.

##### Ecological note.

Oak canopies of *Quercus robur*. Phenology: Jun.–Jul.

#### Scatopsciara (Scatopsciara) fluviatilis

Taxon classificationAnimaliaDipteraSciaridae

(Lengersdorf, 1940)

26CDB2A0-8D66-5BD6-B9BD-B2758A33E53F

##### Synonyms.

= *coei* Freeman, 1983; = pulchra (Lengersdorf, 1940); = robusticornis (Frey, 1948).

##### Literature.

*Faunistics*: Menzel et al. (1990): 326 [as *Scatopsciara fluviatilis*]; Tuomikoski (1960): 155 [as *Scaptosciara fluviatilis*; recte *Scatopsciara*]. *Taxonomy*: Tuomikoski (1960): 151, 155 [as *Scaptosciara fluviatilis*; recte *Scatopsciara*]; Freeman (1983): 167 [as *Scatopsciara coei*]; Menzel and Mohrig (2000): 486 [as Scatopsciara (Scatopsciara) fluviatilis].

##### Localities.

• Norway; without further locality details (= ‘Norwegen’) • Finnmark; Tana, Tanafjorden, fjord Vestertana (= ‘Finmark, Vestertana’) • Troms; Tromsø (= ‘Tromsø’).

##### Ecological note.

Habitats not specified. Phenology: Aug.

#### Scatopsciara (Scatopsciara) multispina

Taxon classificationAnimaliaDipteraSciaridae

(Bukowski & Lengersdorf, 1936)

A4540463-CFB6-52ED-AFDF-C860C16D594B

##### Literature.

*Faunistics*: Köhler et al. (2014): 329 [as Scatopsciara (Scatopsciara) multispina]. *Taxonomy*: Tuomikoski (1960): 150, 152 [as *Scaptosciara multispina*; recte *Scatopsciara*]; Menzel and Mohrig (2000): 492 [as Scatopsciara (Scatopsciara) multispina].

##### Localities.

• Hordaland; Kvam, ‘Berge landskapsvernområde’ [protected landscape area with the Bergsvatnet] NW of Tørvikbygd (= ‘Kvam, Berge’) • Telemark; Drangedal, woodland Steinknapp SW of Drangedal (= ‘Drangedal, Steinknapp’).

##### Faunistic note.

The first specimens of *Scatopsciara multispina* from Norway were identified in our NTI project 2014–2016.

##### Ecological note.

Oak canopies of *Quercus robur*. Phenology: Jun.

#### Scatopsciara (Scatopsciara) nana

Taxon classificationAnimaliaDipteraSciaridae

(Winnertz, 1871)

8C92CF85-D661-5B94-8C43-629464A01847

##### Synonym.

= felti (Pettey, 1918).

##### Literature.

*Faunistics*: Lengersdorf (1926b): 3 [as *Sciara nana*] and 4 [as *Sciara intermista*; misidentification]; Soot-Ryen (1942): 79 [in part as *Neosciara nitidula*; misidentification (only cited *nana* and *intermista* specimens)]. *Taxonomy*: Menzel and Mohrig (2000): 492; Mohrig et al. (2013): 239 [both as Scatopsciara (Scatopsciara) nana].

##### Localities.

• Finnmark; Alta, Jotkajavre fjellstue on the Finnmarksvidda between Karasjok and Alta (= ‘Jotkajavre’) • Karasjok, Karasjok at the river Karasjohka (= ‘Karasjok’) • Rogaland; Sandnes, Sandnes S of Stavanger (= ‘Sandnes’) • Troms; Balsfjord, Nordkjosbotn 70 km SE of Tromsø (= ‘Nordkjosbotn’) • Karlsøy, Torsvåg at the NW coast of Vannøya 15 km N of Tromsø (= ‘Torsvåg’) • Målselv, farm Frihetsli in the Dividalen 32 km SE of Øverbygd (= ‘Frihetsli’) • Tromsø, lake Prestvannet on the Tromsøya (= ‘Prestvand’; = ‘Prestvann, Tromsø’) • Trøndelag; Levanger, Levanger (= ‘ad Levanger’; = ‘Levanger’).

##### Ecological note.

Habitats not specified. Phenology: Jun.–Aug.

#### Scatopsciara (Scatopsciara) neglecta

Taxon classificationAnimaliaDipteraSciaridae

Menzel & Mohrig, 1998

F7A63524-80D2-526E-89A1-41723BE3F50D

##### Literature.

*Faunistics*: Köhler et al. (2014): 329 [as Scatopsciara (Scatopsciara) neglecta]. *Taxonomy*: Menzel and Mohrig (1998): 370; Menzel and Mohrig (2000): 498 [both as Scatopsciara (Scatopsciara) neglecta].

##### Locality.

• Telemark; Drangedal, 300 m SE of Henneseid (= ‘Drangedal, Henseid’).

##### Faunistic note.

The first specimen of *Scatopsciara neglecta* from Norway was identified in our NTI project 2014–2016.

##### Ecological note.

Oak canopies of *Quercus robur*. Phenology: Jul.

#### Scatopsciara (Scatopsciara) pusilla

Taxon classificationAnimaliaDipteraSciaridae

(Meigen, 1818)

957A8D10-28DB-554E-8416-C12A1B362280

##### Synonyms.

= paludicicola (Lengersdorf, 1940); = *pavida* (Winnertz, 1867); = pusilliformis Mohrig & Mamaev, 1986; = zygoneuroides Frey, 1948.

##### Literature.

*Faunistics*: Lengersdorf (1926b): 3 [as *Sciara pavida*]; Soot-Ryen (1942): 80 [as *Neosciara pusilla*]. Menzel et al. (1990): 327 [as *Scatopsciara pusilla*]; Köhler et al. (2014): 329 [as Scatopsciara (Scatopsciara) pusilla]. *Taxonomy*: Tuomikoski (1960): 151, 155 [as *Scaptosciara pusilla*; recte *Scatopsciara*]; Menzel and Mohrig (1998): 370; Menzel and Mohrig (2000): 499 [both as Scatopsciara (Scatopsciara) pusilla].

##### Localities.

• Norway; without further locality details (= ‘Norwegen’) • Finnmark; Alta, Jotkajavre fjellstue on the Finnmarksvidda between Karasjok and Alta (= ‘Jotkajavre’) • Telemark; Drangedal, woodland Steinknapp SW of Drangedal (= ‘Drangedal, Steinknapp’).

##### Ecological note.

Oak canopies of *Quercus robur*. Phenology: Jun., Aug.

#### Scatopsciara (Scatopsciara) vitripennis

Taxon classificationAnimaliaDipteraSciaridae

(Meigen, 1818)

FAF81DDC-12B7-5DDB-914B-503DD442BBAC

##### Synonyms.

= actuosa (Johannsen, 1912); = aucta (Winnertz, 1867); = basaliseta (Yang & Zhang, 1987); = *coracina* (Zetterstedt, 1851); = *intermista* (Winnertz, 1867); = *nitidula* (Zetterstedt, 1851); = *quinquelineata* (Macquart, 1834); = superba (Winnertz, 1867).

##### Literature.

*Faunistics*: Zetterstedt (1851): 3739 [as *Sciara coracina*] and 3760 [as *Sciara nitidula*]; Siebke (1863): 176; Siebke (1866a): 388 [both as *Sciara quinquelineata* Macquart; recte *quinquelineata* Macquart]; Siebke (1877): 213 [as *Sciara coracina* and *Sciara quinquelineata*] and 214 [as *Sciara nitidula*]; Soot-Ryen (1942): 80 [as *Scatopsciara vitripennis*; in part (only cited *coracina*, *quinquelineata* and *vitripennis* specimens)]; Tuomikoski (1960): 152 [as *Scaptosciara vitripennis*; recte *Scatopsciara*]; Menzel and Mohrig (2000): 487 [as *Sciara coracina* and *Sciara nitidula* under Scatopsciara (Scatopsciara) vitripennis]; Köhler et al. (2014): 329 [as Scatopsciara (Scatopsciara) vitripennis]. *Taxonomy*: Tuomikoski (1960): 150, 151 [as *Scaptosciara vitripennis*; recte *Scatopsciara*]; Menzel and Mohrig (2000): 487; Mohrig et al. (2013): 240 [both as Scatopsciara (Scatopsciara) vitripennis].

##### Localities.

• Finnmark; Tana, between Porsangerfjorden and fjord Vestertana (= ‘Finmark, zwischen Porsangerfjord und Vestertana’) • Hordaland; Kvam, ‘Berge landskapsvernområde’ [protected landscape area with the Bergsvatnet] NW of Tørvikbygd (= ‘Kvam, Berge’) • Møre Og Romsdal; Haram, ? Ormeneset (= in Romsdalia ad Ormen’; = ‘Romsdals Amt, omkring Ormen’; = ‘Ormem, Romsdal’) • Oppland; Lesja, Fogstuen on the Dovrefjell plateau (= ‘Fogstuen’; = ‘Fokstuen, Dovre’; = ‘in alpe Dovre ad Fogstuen’; = ‘in alpe Dovre’) • Oslo; Oslo, Botanisk hage (= ‘ad Christianiam in horto botanico’; = ‘Botanical Garden, Oslo’) • Oslo, Tøyen (= ‘ad Christianiam in Tøien’; = ‘in Töien prope Christianiam’; = ‘Töien [Oslo]’; = ‘Töien’) • Telemark; Drangedal, woodland Steinknapp SW of Drangedal (= ‘Drangedal, Steinknapp’) • Trøndelag; Levanger, Levanger (= ‘ad urbem Levanger’) • Levanger, Skogn SE of Levanger (= ‘ad diversorium Thynæs et urbem Levanger in paroecia Skogn’; = ‘ad Thyæs in parochia Skogn’; = ‘Thynäs’) [= in the accommodation of Thy in Skogn] • Oppdal, Kongsvoll near Kongsvold Fjeldstue in the Drivdalen (= ‘Kongsvold’; = ‘Kongsvold, Dovre’= ‘in alpe Dovre ad Kongsvold’; = ‘in alpe Dovre’).

##### Ecological note.

On coasts and in botanical gardens; oak canopies of *Quercus robur*. Phenology: May–Aug.

#### Schwenckfeldina carbonaria

Taxon classificationAnimaliaDipteraSciaridae

(Meigen, 1830)

DDDBBAE9-BDD0-520B-8BB9-6BFB8A0D4748

##### Synonyms.

= frauenfeldi (Winnertz, 1867); = illepida (Winnertz, 1867); = indigena (Winnertz, 1867), = *pilosa* Antonova, 1975.

##### Literature.

*Faunistics*: Zetterstedt (1851): 3717; Siebke (1870): 304; Siebke (1877): 210 [all as *Sciara carbonaria*]; Soot-Ryen (1942): 77 [as *Neosciara carbonaria*]. *Taxonomy*: Tuomikoski (1960): 29; Menzel and Mohrig (2000): 510 [both as *Schwenckfeldina carbonaria*].

##### Localities.

• Buskerud: Bjøberg in the Hemsedalsfjella between Hemsedal and Lærdal (= ‘Bjøberg paa Hemsedalsfjeldet’; = ‘ad Bjøberg in alpe Hemsedalsfjeld’; = ‘Bjøberg, Hemsedal’) • Røyken (= ‘in parochia Røken’; = ‘Røken’; = ‘Røyken’) • Oslo; Oslo (= ‘ad Christianiam’; = ‘Oslo’) • Oslo, Skøyen (= ‘Skøien’; = ‘Skøyen’) • Oslo, Tøyen (= ‘Tøien’; = ‘Tøyen’).

##### Ecological note.

Habitats not specified. Phenology: Jun.–Jul.

#### Schwenckfeldina tridentata

Taxon classificationAnimaliaDipteraSciaridae

(Rübsaamen, 1898)

4E1FA848-3F9D-5FBA-8546-B4C1EF486AD3

##### Synonyms.

= *atrata* (Holmgren, 1869) [preocc.]; = *holmgreni* (Jacobson, 1898) [preocc.]; = *incisiforceps* (Frey, 1948), = *laguncularis* (Lengersdorf, 1930); = validicornis (Lundbeck, 1898).

##### Literature.

*Faunistics*: Holmgren (1869): 15, 51 [as *Sciara atrata*]; Edwards (1922): 196; Edwards (1923): 236; Summerhayes and Elton (1923): 240 [all as *Sciara tridentata*]; Lengersdorf (1930a): 55 [as *Sciara atrata*]; Lengersdorf (1930c): 52 [as *Rhynchosciara laguncularis*]; Thor (1930): 31; Edwards (1935): 532 [both as *Sciara tridentata*]; Frey (1948): 77, 89 [as Bradysia (Neosciara) incisiforceps]; Tuomikoski (1967): 46 [as *Schwenckfeldina tridentata*]; Menzel and Mohrig (2000): 513 [as *Sciara atrata* and *Rhynchosciara laguncularis* under *Schwenckfeldina tridentata*]; Coulson and Refseth (2004): 103; Hågvar et al. (2007): 67; Coulson (2008): 162; Coulson (2013): 155; Mohrig et al. (2013): 246 [all as *Schwenckfeldina tridentata*]. *Taxonomy*: Tuomikoski (1966): 137; Tuomikoski (1967): 45; Menzel and Mohrig (2000): 513; Mohrig et al. (2013): 246 [all as *Schwenckfeldina tridentata*].

##### Localities.

• Jan Mayen: without further locality details (= ‘Jan Mayen Island’) • Svalbard; Bjørnøya (= ‘Bear Island’) • Bjørnøya, bay Austervåg at the E coast (= ‘bei Austervåg (B.)’; = ‘Spitzbergen, bei Austervåg’) • Bjørnøya, Brettingsdalen at the E side of Miseryfjellet (= ‘Bear Island, Brettingsdalen’) • Bjørnøya, Nordcapp at the NE coast (= ‘Spetsberg, Nordcap’) [misinterpretation in Menzel and Mohrig (2000), not ‘Spetsberg, Nordcap (= Spitzbergen, Nordfjorden)’] • Spitsbergen, Adventdalen near Adventfjorden at the W coast (= ‘Adventdalen’) • Spitsbergen, Amsterdamøya, Smeerenburg at the SE coast (= ‘in Spetsbergia ad Smeerenberg’; = ‘Spitzbergen, Smeerenberg’; = ‘Nordfjorden, Smeerenburg’) • Spitsbergen, Bellsund at the W coast (= ‘in Spetsbergia ad Belsund’; = ‘Bellsund’; = ‘Belsund’) • Spitsbergen, Grønfjorden (= ‘in Spetsbergia ad Green Harbour’; = ‘Spetsbergia, Green Harbour’; = ‘Spetsbergia ad Green Harbour [Spitzbergen, bei Green Harbour]’; = ‘Green Harbour’) • Spitsbergen, Kapp Linné by the Isfjord, Isfjord Radio station (= ‘Svalbard, Isfjord Radio’) • Spitsbergen, Kobbefjorden at the NW coast near the Danskøya (= ‘in Spetsbergia ad Kobbebay’; = ‘Spitzbergen, Kobbebay’; = ‘Kobbebay’) • Spitsbergen, Nordaustlandet (= ‘North-East Land’) • Spitsbergen, Nordaustlandet, Murchisonfjorden (= ‘North-East Land, Murchison Bay’) • Spitsbergen, Nordfjorden between Bohemanneset and Kapp Thordsen (= ‘in Spetsbergia ad Nordfjorden’) • Spitsbergen, Prins Karls Forland at the W coast of Oscar II Land (= ‘Prince Charles Foreland (S.)’) • Spitsbergen, Prins Karls Forland at the W coast of Oscar II Land, between Richardlaguna and Carmichaelpynten (= ‘Spitsbergen, Prince Charles Foreland (North Eastern Region), from Richard Lagoon to Point Carmichael’) • Spitsbergen, Prins Karls Forland at the W coast of Oscar II Land, Carmichaelpynten (= ‘Spitsbergen, Prince Charles’ Foreland, Pt. Carmichael, Freshwater Bay district, N.E. of island’) • Spitsbergen, Prins Karls Forland at the W coast of Oscar II Land, Ferskvassbukta at the NE coast (= ‘Prince Charles Foreland, Freshwater Bay’) • Spitsbergen, S coast of Kongsfjorden, W of Ny-Ålesund (= ‘NW Spitsbergen, South coast of Kongsfjord, W of Ny Ålesund’) • Spitsbergen, without further locality details (= ‘Spetsbergen; = ‘Spitsbergen’; = ‘Spitzbergen’).

##### Ecological note.

In dry ridges and slopes with *Saxifraga oppositifolia*, mosses and lichens; in mosses and lichens; among stones and plants (e.g. *Buellia sorotia*, *Dicranoweisia crispula*, *Parmelia alpicola*, *Saxifraga oppositifolia*, *Salix polaris*); on stones of shingly raised beaches (all Svalbard records). Phenology: Jun.–Jul., Sep.

#### Sciara flavimana

Taxon classificationAnimaliaDipteraSciaridae

Zetterstedt, 1851

814EADB6-40E2-5D1A-8B47-FBB0C8FF7FCD

##### Synonyms.

= fulgens Winnertz, 1867, = mannii Winnertz, 1867.

##### Literature.

*Faunistics*: Siebke (1866a): 385; Siebke (1877): 211 [both as *Sciara flavimana*]; Soot-Ryen (1942): 76 [as *Lycoria flavimana*]; Menzel et al. (1990): 311; Menzel (1992): 267; Komarova (2006): 54 [all as *Sciara flavimana*]. *Taxonomy*: Antonova (1978): 182, 185; Menzel and Mohrig (2000): 530 [both as *Sciara flavimana*].

##### Localities.

• Norway; without further locality details (= ‘Norwegia’; = ‘Norwegen’) • Møre Og Romsdal; Rauma, between Veblungsnes and Romsdalshornet Mountain in the Romsdalsalpene SE of Åndalsnes (= ‘Romsdals Amt, mellem Veblungsnæsset og Romsdalshorn’) • Rauma, Veblungsnes at the Romsdalsfjorden SW of Åndalsnes (= ‘ad Veblungsnæs Romsdaliæ; = ‘Veblungsnes, Romsdal’) • Oslo; Oslo, Tøyen (= ‘in Tøien ad Christ.’; = ‘Tøyen, Oslo’).

##### Ecological note.

Habitats not specified. Phenology: Jul.–Aug.

#### Sciara hemerobioides

Taxon classificationAnimaliaDipteraSciaridae

(Scopoli, 1763)

80129874-0DF2-54FE-B5BB-0659682EFB86

##### Synonyms.

= lateralis Meigen, 1818; = *morio* (Fabricius, 1794); = *thomae* (Linnaeus, 1767); = valida Winnertz, 1867.

##### Literature.

*Faunistics*: Zetterstedt (1851): 3714; Siebke (1853): 305; Zetterstedt (1855): 4888; Siebke (1866a): 384, 387; Siebke (1866b): 417; Siebke (1870): 304; Siebke (1872): 96; Siebke (1877): 210; Strand (1904): 9; Lengersdorf (1926b): 3 [all as *Sciara thomae*]; Soot-Ryen (1942): 76 [as *Lycoria thomae*]; Menzel et al. (1990): 313 [as *Sciara thomae*]. *Taxonomy*: Antonova (1978): 181, 182 [as *Sciara thomae*]; Menzel and Mohrig (2000): 520; Sutou et al. (2004): 179; Komarova (2006): 52 [all as *Sciara hemerobioides*].

##### Localities.

• Norway; without further locality details (= ‘Norwegen’; = ‘Norwegia’) • Buskerud; Ål (= ‘Aal’) • Ringerike, farm Tanberg in Norderhov 5 km S of Hønefoss (= ‘Tandberg i Nordrehaug’) • Ringerike, Norderhov 5 km S of Hønefoss (= ‘in par. [parochia] Nordrehaug Ringerikiæ’; = ‘Nordrehaug Ringerikiæ’; = ‘Norderhov, Ringerike’; = ‘in Ringerike’) • Hedmark; Åmot, in the Østerdalen (= ‘Østerdalen, Aamodt’; = ‘Aamodt’) • Tynset, Tylldalen in the Østerdalen (= ‘Tyldal Østerdaliæ’; = ‘Østerdalen, Tyld. len’; = ‘Tyldal’) • Møre Og Romsdal; Rauma, Horgheim SE of Åndalsnes in the Romsdalen (= ‘Romsdals Amt, Horgheim’) • Rauma, Rauma in the Romsdalen (= ‘Romsdals Amt, i Rauma’) • Rauma, in the Romsdalen (= ‘ad Fladmark, Romsdaliæe’; = ‘Fladmark, Romsdal’) • Oppland; Nord-Fron or Sør-Fron in the Gudbrandsdalen (= ‘Gudbrandsdalen, Fron’) • Øyer in the Gudbrandsdalen (= ‘Øier Gudbrandsdaliæ’; = ‘Gudbrandsdalen, Öier’; = ‘Øyer’) • Oslo; Oslo (= ‘circa Christianiam’; = ‘Kristiania’; = ‘Oslo’) • Oslo, Tøyen (= ‘circa Christianiam’; = ‘circa Christianiam ... in Tøien’; = ‘Tøyen, Oslo’) • Østfold; Halden, Halden SE of Fredrikstad (= ‘ad Fredrikshald’; = ‘Fredrikshald’) • Sarpsborg, Sarpsborg NE of Fredrikstad (= ‘Sarpsborg’).

##### Ecological note.

On flowers of *Pimpinella saxifraga* and *Scabiosa*; between stones on sandy soil. Phenology: Jul.–Sep.

#### Sciara humeralis

Taxon classificationAnimaliaDipteraSciaridae

Zetterstedt, 1851

7E048A67-305F-5FE9-A6C2-519552AEEC42

##### Synonyms.

= analis var. bezzii Del Guercio, 1905; = armata Winnertz, 1867; = hamatilis Yang, Zhang & Yang, 1993.

##### Literature.

*Faunistics*: Zetterstedt (1851): 3718; Siebke (1877): 210 [both as *Sciara humeralis*]; Soot-Ryen (1942): 75 [as *Lycoria humeralis*]; Hansen and Falck (2000): 18; Menzel et al. (1990): 312; Menzel and Mohrig (1991): 13; Menzel and Mohrig (2000): 528; Sutou et al. (2004): 187; Komarova (2006): 54 [all as *Sciara humeralis*]. *Taxonomy*: Antonova (1978): 182, 187; Menzel and Mohrig (1991): 13; Menzel and Mohrig (2000): 528; Sutou et al. (2004): 187 [all as *Sciara humeralis*].

##### Localities.

• Norway; without further locality details (= ‘Norwegia’; = ‘Norwegen’) • Buskerud; Ringerike NE of Oslo (= ‘in Ringerige Norwegiæ’; = ‘Ringerige Norwegiae’; = ‘Ringerige’; = ‘Ringerike’) • Oslo; Oslo, Botanisk hage (= ‘ad Christianiam in horto botanico’; = ‘Botanical Garden, Oslo’) • Østensjø, lake Østensjøvannet SE of Oslo (= ‘Østensjøvannet vel 5 km fra Oslo sentrum’).

##### Ecological note.

In botanical gardens. Phenology: May, Aug.

#### Sciara ruficauda

Taxon classificationAnimaliaDipteraSciaridae

Meigen, 1818

66CB1EF7-1D20-5DD3-B189-6271DDACB677

##### Synonyms.

= boleti Winnertz, 1867; = mamaevi Antonova, 1978; = vigilax Winnertz, 1867.

##### Literature.

*Faunistics*: Zetterstedt (1852): 4354; Siebke (1863): 176; Siebke (1877): 210; Lengersdorf (1926b): 3 [all as *Sciara ruficauda*]; Soot-Ryen (1942): 76 [as *Lycoria ruficauda*]; Menzel et al. (1990): 312 [as *Sciara ruficauda*]. *Taxonomy*: Antonova (1978): 182, 186 [as Sciara*mamaevi*]; Menzel and Mohrig (2000): 530 [as *Sciara ruficauda*].

##### Localities.

• Norway; without further locality details (= ‘Norwegen’) • Oppland; Lesja, Fogstuen on the Dovrefjell plateau (= ‘ad Fogstuen’; = ‘in alpe Dovre ad Fokstuen’; = ‘Fokstuen, Dovre’) • Oslo; Oslo, Tøyen (= ‘ad Christianiam in Tøien’; = ‘ad Tøien’; = ‘Tøyen, Oslo’) • Troms; Målselv, farm Frihetsli in the Dividalen 32 km SE of Øverbygd (= ‘Frihetsli’).

##### Ecological note.

Habitats not specified. Phenology: Jun.–Jul.

#### Trichocoelina brevicubitalis

Taxon classificationAnimaliaDipteraSciaridae

(Lengersdorf, 1926)

70968F44-D926-5739-8244-D52491D3360A

##### Literature.

*Faunistics*: Lengersdorf (1926b): 6 [as *Sciara brevicubitalis*]; Soot-Ryen (1942): 77 [as *Neosciara brevicubitalis*]; Menzel and Mohrig (2000): 408 [as Lycoriella (Hemineurina) brevicubitalis]. *Taxonomy*: Menzel and Mohrig (2000): 408 [as Lycoriella (Hemineurina) brevicubitalis]; Vilkamaa and Menzel (2019): 19, 53 [as *Trichocoelina brevicubitalis*].

##### Localities.

• Finnmark; Alta, Bojobæskihytta in the Stabbursdalen between Karasjok and Alta (= ‘Bojobæske’) • Alta, Jotkajavre fjellstue on the Finnmarksvidda between Karasjok and Alta (= ‘Jotkajavre’) • Karasjok, Karasjok at the river Karasjohka (= ‘Karasjok’) • Nordland; Sørfold, Røsvik on the S shore of Sørfolda (= ‘Røsvik’).

##### Ecological note.

Habitats not specified. Phenology: Jul.–Aug.

#### Trichocoelina cochleata

Taxon classificationAnimaliaDipteraSciaridae

(Rübsaamen, 1898)

B2EC4015-CC33-5E4D-B728-406F301A98FC

##### Synonym.

= haemorrhoidalis (Lundbeck, 1898).

##### Literature.

*Faunistics*: Soot-Ryen (1942): 77 [as *Neosciara cochleata*]; Tuomikoski (1960): 76; Tuomikoski (1967): 47; Coulson and Refseth (2004): 103; Coulson (2008): 161; Coulson (2013): 154; Mohrig et al. (2013): 270 [all as Lycoriella (Hemineurina) cochleata]. *Taxonomy*: Tuomikoski (1960): 75, 76; Menzel and Mohrig (2000): 409; Mohrig et al. (2013): 270 [all as Lycoriella (Hemineurina) cochleata]; Vilkamaa and Menzel (2019): 16, 21, 53 [as *Trichocoelina cochleata*].

##### Localities.

• Finnmark; Vardø, Varangerhalvøya, Persfjorden (= ‘Vardö, Persfjord’).

• Svalbard; Spitsbergen, Longyearbyen (= ‘Longyearbyen’) • Spitsbergen, without further locality details (= ‘Spitsbergen’).

##### Ecological note.

Habitats not specified. Phenology: Jul.–Aug.

#### Trichocoelina ithyspina

Taxon classificationAnimaliaDipteraSciaridae

Vilkamaa & Menzel, 2019

4748E743-9E5B-526C-88BE-2E2E9820C239

##### Literature.

*Faunistics*: Vilkamaa and Menzel (2019): 29 [as *Trichocoelina ithyspina*]. *Taxonomy*: Vilkamaa and Menzel (2019): 15, 29, 53 [as *Trichocoelina ithyspina*].

##### Locality.

• Hedmark; Stor-Elvdal, at the river Atna, Solbakken NW of Koppang (= ‘Stor-Elvdal, Atna River, Solbakken’).

##### Faunistic note.

The first specimen (holotype) of *Trichocoelina ithyspina* from Norway was prepared and identified in our NTI projects 2014–2018.

##### Ecological note.

Habitats not specified. Phenology: Jun.–Jul.

#### Trichocoelina jukkai

Taxon classificationAnimaliaDipteraSciaridae

Vilkamaa & Menzel, 2019

B0893CC2-983F-5BC8-9E93-052D08D234BA

##### Literature.

*Faunistics*: Vilkamaa and Menzel (2019): 33 [as *Trichocoelina jukkai*]. *Taxonomy*: Vilkamaa and Menzel (2019): 15, 33, 53 [as *Trichocoelina jukkai*].

##### Locality.

• Troms; Tromsø, Nakkedalen, S of Estengammen.

##### Faunistic note.

The first specimens (2 paratypes) of *Trichocoelina jukkai* from Norway were identified in our NTI project 2017–2018.

##### Ecological note.

Habitats not specified. Phenology: Jul.

#### Trichocoelina obesula

Taxon classificationAnimaliaDipteraSciaridae

Vilkamaa & Menzel, 2019

71F1687B-3B92-5D54-942D-B686293ACAF0

##### Literature.

*Faunistics*: Vilkamaa and Menzel (2019): 35 [as *Trichocoelina obesula*]. *Taxonomy*: Vilkamaa and Menzel (2019): 15, 35, 53 [as *Trichocoelina obesula*].

##### Locality.

• Svalbard; Bjørnøya, at the Engelskelva in the NE part of island (= ‘Svalbard, Engelskelva’) • Bjørnøya, at the Lakselva (= ‘Svalbard, Lakselva’).

##### Faunistic note.

The first specimens (holotype, 2 paratypes) of *Trichocoelina obesula* from Norway were identified in our NTI project 2017–2018.

##### Ecological note.

Habitats not specified. Phenology: Jul.

#### Trichocoelina oricillifera

Taxon classificationAnimaliaDipteraSciaridae

Vilkamaa & Menzel, 2019

49427ADF-BF0B-5C42-BA7F-3DFACE2A15A4

##### Literature.

*Faunistics*: Vilkamaa and Menzel (2019): 40 [as *Trichocoelina oricillifera*]. *Taxonomy*: Vilkamaa and Menzel (2019): 15, 40, 53 [as *Trichocoelina oricillifera*].

##### Localities.

• Finnmark; Karasjok, Karasjok at the river Karasjohka (= ‘Karasjok’) • Tana, Storfossen at the river Karasjohka near the Finnish border (= ‘Tana, Nedre Storfoss’).

##### Faunistic note.

The first specimens (2 paratypes) of *Trichocoelina oricillifera* from Norway were identified in our NTI project 2017–2018.

##### Ecological note.

Habitats not specified. Phenology: Jul.–Aug.

#### Trichocoelina semisphaera

Taxon classificationAnimaliaDipteraSciaridae

Vilkamaa & Menzel, 2019

92A6A673-8E92-5D0E-AC90-425E0973FD9A

##### Literature.

*Faunistics*: Vilkamaa and Menzel (2019): 43 [as *Trichocoelina semisphaera*]. *Taxonomy*: Vilkamaa and Menzel (2019): 16, 43, 53 [as *Trichocoelina semisphaera*].

##### Locality.

• Svalbard; Bjørnøya, at the Lakselva (= ‘Svalbard, Lakselva’).

##### Faunistic note.

The first specimen (paratype) of *Trichocoelina semisphaera* from Norway was identified in our NTI project 2017–2018.

##### Ecological note.

Habitats not specified. Phenology: Jul.

#### Trichocoelina vitticollis

Taxon classificationAnimaliaDipteraSciaridae

(Holmgren, 1883)

419A062A-0BDD-5A02-938F-60B2FBC8009F

##### Synonyms.

= *glacialis* (Lundbeck, 1898) [preocc.]; = *permutata* (Lundbeck, 1900).

##### Literature.

*Faunistics*: Tuomikoski (1967): 48 [as Lycoriella (Hemineurina) permutata]; Menzel and Mohrig (2000): 411 [as *Sciara permutata* under Lycoriella (Hemineurina) vitticollis]; Coulson and Refseth (2004): 103; Coulson (2008): 162; Coulson (2013): 154; Mohrig et al. (2013): 271 [all as Lycoriella (Hemineurina) vitticollis]; Vilkamaa and Menzel (2019): 47 [as *Trichocoelina vitticollis*]. *Taxonomy*: Tuomikoski (1960): 75, 76 [as Lycoriella (Hemineurina) permutata]; Menzel and Mohrig (2000): 411; Mohrig et al. (2013): 271 [both as Lycoriella (Hemineurina) vitticollis]; Vilkamaa and Menzel (2019): 16, 47, 53 [as *Trichocoelina vitticollis*].

##### Localities.

• Svalbard; Bjørnøya (= ‘Bear Island’) • Bjørnøya, at the Lakselva (= ‘Svalbard, Lakselva’) • Spitsbergen, Adventdalen near Adventfjorden at the W coast (= ‘Adventdalen’) • Spitsbergen, Albert I Land, Lillehøkfjorden, E part of Mitrahalvøya, Nilspynten (= ‘Svalbard, Lillehoeoekfjorden, Nilspynten’) • Spitsbergen, Fjortende Julibukta on the E side of Krossfjorden (= ‘Svalbard, Krossfjorden, 14. juli bukta’) • Spitsbergen, Kobbefjorden at the NW coast near the Danskøya (= ‘Kobbefjorden [Kobbebay]’) • Spitsbergen, Nordenskiöld Land, Bjørndalen W of Adventfjorden (= ‘Svalbard, Bjorndalen’) • Spitsbergen, Nordenskiöld Land, Bolterdalen on the S side of Adventdalen (= ‘Svalbard, Bolterdalen’) • Spitsbergen, Nordenskiöld Land, Colesbukta on the S side of Isfjorden (= ‘Svalbard, Colesbukta’) • Spitsbergen, Nordenskiöld Land, Hanaskogdalen on the E side of Adventfjorden (= ‘Svalbard, Hanaskogdalen’) • Spitsbergen, Nordenskiöld Land, Longyearbyen in the Longyeardalen S of Adventfjorden (= ‘Svalbard, Longyearbyen’) • Spitsbergen, S coast of Kongsfjorden, W of Ny-Ålesund (= ‘NW part of Spitsbergen, S coast of Kongsfjord, W of Ny Ålesund’; = ‘NW-Spitzbergen, Kongsfjord, Südküste, westlich von Ny Ålesund’) • Spitsbergen, Virgohamna at the N coast of Danskøya (= ‘Danskøya, Virgohamna’) • Spitsbergen, without further locality details (= ‘Spitsbergen’).

##### Ecological note.

Under stones (some Svalbard records). Phenology: Jul.–Aug.

#### Trichosia (Mouffetina) expolita

Taxon classificationAnimaliaDipteraSciaridae

(Coquillett, 1900)

545BC388-7215-5193-BA2F-3B297E8752D7

##### Synonyms.

= abdita (Johannsen, 1912); = clavata (Garrett, 1925); = *filispina* Menzel & Mohrig, 1997.

##### Literature.

*Faunistics*: Menzel and Mohrig (1997): 32 [as Trichosia (Mouffetina) filispina]; Mohrig et al. (2013): 256 [as Trichosia (Mouffetina) filispina under Trichosia (Mouffetina) expolita]; Vilkamaa et al. (2013): 25 [as *Mouffetina expolita*]. *Taxonomy*: Menzel and Mohrig (1997): 32; Menzel and Mohrig (2000): 551 [both as Trichosia (Mouffetina) filispina]; Mohrig et al. (2013): 256 [as Trichosia (Mouffetina) expolita].

##### Localities.

• Norway; without further locality details (= ‘Norway’) • Finnmark; Sør-Varanger, Pasvik Valley near lake Vaggatem (= ‘Pasvik-Tal bei Vaggatem’; = ‘Pasvik Valley near Vaggatem’).

##### Ecological note.

Habitats not specified. Phenology: without data.

#### Trichosia (Trichosia) caudata

Taxon classificationAnimaliaDipteraSciaridae

(Walker, 1848)

9AC7B5C4-AE90-57EE-AF82-FE6ECBD3CD7F

##### Synonyms.

= dziedzickii (Grzegorzek, 1884); = *longiventris* (Zetterstedt, 1851); = mikii (Grzegorzek, 1884); = sznablii (Grzegorzek, 1884).

##### Literature.

*Faunistics*: Zetterstedt (1851): 3727; Siebke (1863): 110; Siebke (1866a): 387; Siebke (1866b): 417; Siebke (1870): 304; Siebke (1872): 97; Siebke (1877): 211; Strand (1904): 10; Lengersdorf (1926b): 3 [all as *Sciara longiventris*]; Soot-Ryen (1942): 76 [as *Lycoria longiventris*]; Tuomikoski (1960): 19; Menzel et al. (1990): 314 [both as Trichosia (Trichosia) caudata]; Menzel and Mohrig (1997): 20 [as *Sciara longiventris* under Trichosia (Trichosia) morio sensu Menzel and Mohrig] and 21 [as Trichosia (Trichosia) morio sensu Menzel and Mohrig; misidentification]; Menzel and Mohrig (2000): 558 [as *Sciara longiventris* under Trichosia (Trichosia) morio sensu Menzel and Mohrig; misidentification]. *Taxonomy*: Tuomikoski (1957): 16 [as *Trichosia caudata*]; Tuomikoski (1960): 18, 19 [as Trichosia (Trichosia) caudata]; Menzel and Mohrig (1997): 19; Menzel and Mohrig (2000): 558 [both as Trichosia (Trichosia) morio sensu Menzel and Mohrig; misidentification].

##### Localities.

• Norway; without further locality details (= ‘Norwegen’) • Akershus; Skedsmo, Lillestrøm E of Oslo (= ‘Lillestrømmen in par. [parochia] Skedsmo’; = ‘ad Christianiam, Lillestrømmen’; = ‘Lillestrømmen’; = ‘Skedsmo’) • Buskerud; Krødsherad (= ‘Krødsherred’; = ‘Krydsherred’) • Hedmark; Åmot, Åset 7.5 km N of Åmot in the Østerdalen (= ‘in parochiis Aamodt Østerdaliæ (ad Aaset)’; = ‘Åset, Åmot’) • Åmot, in the Østerdalen (= ‘Østerdalen, Aamodt’) • Møre Og Romsdal; Rauma, Rauma in the Romsdalen (= ‘Romsdals Amt, i Rauma’) • Rauma, in the Romsdalen (= ‘ad Fladmark, Romsdaliæe’; = ‘Fladmark, Romsdal’) • Oppland; Nord-Aurdal, Aurdal (= Aurdal in Valders’; = ‘Aurdal, Valdres’; = ‘Aurdal’) • Vågå, farm Sve NE of Vågåmo in the Gudbrandsdalen (= ‘Vaage Gudbrandsdaliæ ad Svee’; = ‘i Vaage’; = ‘Sve, Våge’) • Oslo; Oslo (= ‘ad Christianiam’; = ‘Oslo’; = ‘Moe.’ [misinterpretation in Menzel and Mohrig (1997), correctly ‘leg. M. Moe’]) • Oslo, Tøyen (= ‘Tøien’; = ‘Tøyen, Oslo’) • Østfold; Hvaler, Hvaløerne (= ‘Hvaløerne’) • Telemark; Porsgrunn, Porsgrunn (= ‘Porsgrund’) • Troms; Nordreisa, woodland and farm Hallen at the E shore of Reisaelva SE of Storslett (= ‘Nordreisa, Hallen’) • Trøndelag; Fosnes, Jøa Island, montain Mulfjellet SE of Dun (= ‘Mulfjellet’) • Levanger, Skogn SE of Levanger (= ‘ad Thynæs’; = ‘ad Thynäs’; = Tynes) [= in the accommodation of Thy in Skogn] • Stjørdal, farm Hammermoen NE of Stjørdal (= ‘ad Hammermoen)’; = ‘Hammermoen’) • Verdal, former poststation ‘Suulstuen’ SE of Vuku at the Jamtlandsvegen [road no. 72] (= ‘ad Suulstuen Værdaliæ’; = ‘Suulstuen Værdaliæ’; = ‘Suul. [Suulstuen Vaerdaliae]’; = ‘Sulstuen’) • Verdal, Kong Carl Johans Klev at the Jamtlandsvegen [road no. 72] SE of Vuku (= ‘ad Kong Carl Johans Klev’; = ‘Kong Carl Joh. Klev. [Kong Carl Johans Klev]’; = ‘ad Carl Johans Klev’; = ‘Karl Johans Klev’) • Verdal, Østre Nes at the Jamtlandsvegen [road no. 72] between Verdal and Lysthaugen (= ‘Østre Værdaliæ’; = ‘Østre’; = ‘Østre Nes’; = ‘Østre Næs’; = ‘Östre-Näs’; = ‘Näs’; = ‘Näs [Östre-Näs]’).

##### Ecological note.

Between stones on sandy soil; larvae in rotten wood of gray alder (*Alnus incana*); on mountains. Phenology: Apr., Jun.–Aug.

#### Trichosia (Trichosia) confusa

Taxon classificationAnimaliaDipteraSciaridae

Menzel & Mohrig, 1997

B97DBE5D-99F9-5E64-BEF2-7D3A475FE043

##### Literature.

*Faunistics*: Zetterstedt (1871): 3721 [as *Sciara trochanterata*; in part misidentification]; Lengersdorf (1941): 48 [in part as *Sciara trochanterata*; misidentification (also discussed as *Sciara edwardsi*)]; Tuomikoski (1960): 19; Menzel et al. (1990): 314 [both as Trichosia (Trichosia) trochanterata sensu Edwards; in part]. *Taxonomy*: Tuomikoski (1957) 27 [as *Trichosia edwardsi* sensu Frey; misidentification]; Tuomikoski (1960) 18, 19 [as Trichosia (Trichosia) trochanterata sensu Edwards; misidentification]; Menzel and Mohrig (1997) 14; Menzel and Mohrig (2000) 555 [both as Trichosia (Trichosia) confusa].

##### Locality.

• Trøndelag; Verdal, near Sul, Kongsstuggu [formerly ‘Kongsstuen fjeldstue’] (= ‘Kongsstuen’; = ‘Kongstuen’).

##### Ecological note.

On mountains. Phenology: Jun.

#### Trichosia (Trichosia) edwardsi

Taxon classificationAnimaliaDipteraSciaridae

(Lengersdorf, 1930)

936A95FC-19B4-5E64-9B2E-E7AC54A69AD9

##### Literature.

*Faunistics*: Camaño Portela et al. (2008): 93 [as *Trichosia edwardsi*]. *Taxonomy*: Menzel and Mohrig (1997): 20; Menzel and Mohrig (2000): 559 [both as *Lycoria edwardsi* under Trichosia (Trichosia) morio; misidentification]; Menzel and Heller (2006): 52 [as Trichosia (Trichosia) edwardsi]; Heller et al. (2016): 105 [as *Trichosia edwardsi*].

##### Locality.

• Norway; without further locality details (= ‘Norway’) • Finnmark; Båtsfjord, Varangerhalvøya, Ytre Syltefjord 35 km SE of Båtsfjord (published as ‘Norway’; see faunistic note).

##### Faunistic note.

The single Norwegian record of *Trichosia edwardsi* published in Camaño Portela et al. (2008) as ‘Norway’ (without collecting data) is based on the following material: Norway • 9 ♂♂; ‘Varanger Peninsula, Ytre, Syltefjord, 35 km S Batsfjord’; 7 Jul. 1994; M. Jaschhof leg.; aspirator; PWMP.

##### Ecological note.

Dwarf-shrub tundra. Phenology: Jul.

#### Trichosia (Trichosia) flavicoxa

Taxon classificationAnimaliaDipteraSciaridae

Tuomikoski, 1960

4AA271BB-F78B-53C1-84B7-F11AC9168A0C

##### Literature.

*Faunistics*: Köhler et al. (2014): 329 [as Trichosia (Trichosia) flavicoxa]. *Taxonomy*: Tuomikoski (1960): 18, 19; Menzel and Mohrig (1997): 24; Menzel and Mohrig (2000): 556 [all as Trichosia (Trichosia) flavicoxa].

##### Locality.

• Telemark; Drangedal, woodland Steinknapp SW of Drangedal (= ‘Drangedal, Steinknapp’).

##### Faunistic note.

The first specimen of *Trichosia flavicoxa* from Norway was identified in our NTI project 2014–2016.

##### Ecological note.

Oak canopies of *Quercus robur*. Phenology: Jun.

#### Trichosia (Trichosia) lengersdorfi

Taxon classificationAnimaliaDipteraSciaridae

Heller, Köhler & Menzel, 2016

FBE37803-5D37-50A7-9881-E8E85499ABFA

##### Literature.

*Faunistics*: Heller et al. (2016): 106, 109 [as Trichosia (Trichosia) lengersdorfi]. *Taxonomy*: Heller et al. (2016): 106 [as Trichosia (Trichosia) lengersdorfi].

##### Localities.

• Akershus; Nesodden, Blåbærstien in Nesoddtangen (= ‘Nesodden, Blåbærstien’; = ‘Nesodden, Blåbærstien – Østvendt skråning’ [correctly translated from Norwegian: ‘Blåbærstien, east-facing slope’]) • Nesodden, Ommen at the W side of Nesodden (= ‘Ommen – Sørvendt rasmark’ [correctly translated from Norwegian: ‘Ommen, south-facing scree’]) • Nesodden, W of abandoned settlement Flatebybråten (= ‘Flatebybråten vest’) • Aust-Agder; Birkenes, Birkeland, Nordåsen • Buskerud; Kongsberg, Haugplassen near Raje in the Rajedalen (= ‘Kongsberg, Haugplassen’) • Ringerike, W of Hønefoss, small river Veksalbekken E of Veksalplassen [mouth of the Veksalbekken in the river Sogna] (= ‘Veksalbekken’) • Ringerike, Synneren naturreservat SW of Hønefoss (= ‘Synneren NR’) • Ringerike, W of Hallingby, S of the marsh Langmyra along the stream Sibekken (= ‘S Langmyra – Langs Sibekken’ [correctly translated from Norwegian: ‘S of Langmyra along Sibekken’]) • Hedmark; Kongsvinger, Abborhøgda in the forest Varaldskogen S of Øyermoen [near the Swedish border] (= ‘Abborhøgda’) • Hordaland; Bergen, Bergen, Fløyen mountain, mountain top Fløyfjellet (= Bergen, Fløyfjellet) • Bergen, Bergen, residential area Skansemyren (= ‘Skansemyren’) • Bergen, N of Langetoen (= ‘N Langetoen’) • Bergen, NW of hill Litlelia SE of Bergen, in the Sædalen N of Sædalen school (= ‘Litlelia – Valley Sædalen N of Sædalen skole’) • Eidfjord, settlement Tveit in the Simadalen NE of Eidfjord (= ‘Eidfjord, Simadalen, Tveit’) • Oslo; Oslo, Gaustad in the borough Nordre Aker (= ‘Gaustad – Jubileumsenga’) • Oslo, Ljabru in the borough Nordstrand, at the Ljanselva (= ‘Ljabru, Ljanselva’) • Oslo, borough Nordstrand, at the Ljanselva in the Liadalen (= ‘Nordstrand, Ljanselva, Liadalen’) • Sogn Og Fjordane; Høyanger, NE of Austreim at the N side of Sognefjorden, N of hill Furehaugen (= ‘Høyanger, N Furehaugen’) • Høyanger, Vårstølen NE of Bjordal (= ‘Vårstølen – Nedenfor veien’ [correctly translated from Norwegian: ‘Vårstølen, below the road’) • Lærdal, Eråksdalen SE of Lærdalsøyri (= ‘Eråksdalen’) • Lærdal, near settlement Voldum N of Borgund (= ‘Lærdal, Eisurda’) • Luster, NE of Gjerde, between river Jostedøla and road no. 334 near the stream Flatelvi (= ‘Luster, Flatelvi – Ved Rv334’ [correctly translated from Norwegian: ‘by the road no. 334’]) • Luster, NW of Gjerde, at the N shore of Nigardsbrevatnet near the Nigardsbreen parking area (= ‘N Nigardsbrevatnet’) • Luster, NW of Gaupne, near Hurrene at the E bank of river Jostedøla (= ‘SW Hurrene’) • Luster, SE of Gjerde, N from the farm Hesjevoll (= ‘N Hesjevoll’) • Sogndal, NE of Sogndal, above the road no. 55 W of the settlement Steig (= ‘Sogndal, W Steig – Ovenfor veien’ [correctly translated from Norwegian: ‘W of Steig, above the road’) • Telemark; Bamble, Langøya in the Langesundsfjorden, bay at the E side of island (= ‘Langøya – Bukt på østsiden (Langøya I)’ [correctly translated from Norwegian: ‘Langøya, bay at the eastern side (Langøya I)’]) • Porsgrunn, Brevik, forest Dammane in the W part of Brevik (= ‘Brevik, Dammane’) • Tinn, Hovin NW of Kongsberg, Spjeldset SW of Øvre Fjellstul (= ‘Hovin, Spjeldset’) • Tokke, E of Dalen, headland Gunnarshelle at the N coast of the west end of lake Bandak (= ‘WNW Gunnarshelle’) • Trøndelag; Trondheim, Trondheim, Sommerlystvegen (= ‘Sør-Trøndelag, Trondheim, M. Sommerlystvegen – in the garden of nr. 22’) • Vestfold; Larvik, Farmenrøysa mountain NE of Kvelde (= ‘Farmenrøysa Ø’ [correctly: ‘Farmenrøysa, east-facing slope’]) • Larvik, hill Småås N of Larvik (= ‘Larvik, Småås’) • Larvik, N part of Jordstøyp naturreservat in the Lågendalen W of Kvelde (= ‘Jordstøyp N’) • Larvik, Nevlungstranda W of Nevlunghavn, beach Mølen (= ‘Nevlungstranda – Mølen II’) • Larvik, SE of Kvelde, settlement Fjære W of the Fjæreelva (= ‘Fjære’).

##### Faunistic note.

The first specimens of *Trichosia lengersdorfi* from Norway were collected and/or identified in our NTI project 2014–2016.

##### Ecological note.

East- and South-facing mountainsides; on scree of steep slopes and on the tops of woody hillsides; eroded mountains with sandy areas at the foot; on steep slopes with large elms and valuable hardwood trees; mountain birch forests; forests along streams in otherwise muddy terrain; gardens with lawn and some larger trees. Phenology: May–Sep.

#### Trichosia (Trichosia) splendens

Taxon classificationAnimaliaDipteraSciaridae

Winnertz, 1867

E95B0A38-2586-5EDB-B9CE-6F7865753E7D

##### Synonyms.

= maxima Strobl, 1880; = winnertzi Nowicki, 1868.

##### Literature.

*Faunistics*: Menzel and Mohrig (1997): 22; Menzel and Mohrig (2000): 560 [both as Trichosia (Trichosia) splendens in the discussion of Trichosia (Trichosia) morio]. *Taxonomy*: Tuomikoski (1960): 17, 18; Menzel and Mohrig (1997): 10; Menzel and Mohrig (2000): 552 [all as Trichosia (Trichosia) splendens]; Vilkamaa (2000): 71 [as *Trichosia splendens*].

##### Localities.

• Norway; without further locality details (= ‘Norway’; see faunistic note) • Trøndelag; Fosnes, Jøa Island, mountain Mulfjellet SE of Dun (= ‘Mulfjellet’).

##### Faunistic note.

The Norwegian specimen of *Trichosia splendens*, recorded without collecting details in Vilkamaa (2000), could not be found anymore in the UZMH collection (Vilkamaa, pers. comm.).

##### Ecological note.

Habitats not specified. Phenology: without data.

#### Xylosciara (Xylosciara) heptacantha

Taxon classificationAnimaliaDipteraSciaridae

Tuomikoski, 1957

636EC52E-19CC-5FE8-A297-AA36793AA0EA

##### Literature.

*Faunistics*: Hippa and Vilkamaa (2004): 25 [as Xylosciara (Xylosciara) heptacantha]. *Taxonomy*: Tuomikoski (1957): 10 [as *Xylosciara heptacantha*]; Tuomikoski (1960): 92, 96; Menzel and Mohrig (2000): 574; Hippa and Vilkamaa (2004): 23 [all as Xylosciara (Xylosciara) heptacantha].

##### Localities.

• Finnmark; Alta, Leirbotn SE of Kviby, Lakselva at the E side of Altafjorden (= ‘Leirbotn, Lakselva’) • Kvalsund, Skaidi (= ‘Skaidi’) • Rogaland; Finnøy, Finnøy Island, Lasteinvatnet SE of Lastein at the SE coast (= ‘Finnöy, Ledsleinvatnet’) • Trøndelag; Oppdal, Kongsvoll near Kongsvold Fjeldstue in the Drivdalen (= ‘Oppdal, Kongsvall’).

##### Ecological note.

Habitats not specified. Phenology: May–Jul.

#### Xylosciara (Xylosciara) spinata

Taxon classificationAnimaliaDipteraSciaridae

(Pettey, 1918)

069CCA79-505E-5718-A05F-EBC0DF44F1F2

##### Synonym.

= *betulae* Tuomikoski, 1960.

##### Literature.

*Faunistics*: Hippa and Vilkamaa (2004): 20 [as Xylosciara (Xylosciara) betulae]. *Taxonomy*: Tuomikoski (1960): 92, 95; Menzel and Mohrig (2000): 568; Hippa and Vilkamaa (2004): 20 [all as Xylosciara (Xylosciara) betulae]; Mohrig et al. (2013): 264 [as Xylosciara (Xylosciara) spinata].

##### Localities.

• Finnmark; Kvalsund, Skaidi (= ‘Skaidi’) • Rogaland; Finnøy, Finnøy Island, Lasteinvatnet SE of Lastein at the SE coast (= ‘Finnöy, Ledsleinvatnet’) • Trøndelag; Oppdal, Kongsvoll near Kongsvold Fjeldstue in the Drivdalen (= ‘Oppdal, Kungsvoll’).

##### Ecological note.

Habitats not specified. Phenology: May–Jul.

#### Xylosciara (Xylosciara) trimera

Taxon classificationAnimaliaDipteraSciaridae

Tuomikoski, 1960

C2E843CE-15A9-58B2-8DE6-17A4DE059A4F

##### Literature.

*Faunistics*: Köhler et al. (2014): 329 [as Xylosciara (Xylosciara) trimera]. *Taxonomy*: Tuomikoski (1960): 90 [as Xylosciara (Trixylosciara) trimera]; Menzel and Mohrig (2000): 573; Hippa and Vilkamaa (2004): 11 [both as Xylosciara (Xylosciara) trimera].

##### Locality.

• Vestfold; Larvik, lake Skjærsjø near Kvelde NW of Larvik (= ‘Larvik, Skjærsjø’).

##### Faunistic note.

The first specimen of *Xylosciara trimera* from Norway was identified in our NTI project 2014–2016.

##### Ecological note.

Oak canopies of *Quercus robur*. Phenology: Jul.

#### Xylosciara (Xylosciara) validinervis

Taxon classificationAnimaliaDipteraSciaridae

Tuomikoski, 1960

8C9EEA64-8117-5370-B204-9AE874A4508F

##### Literature.

*Faunistics*: Tuomikoski (1960): 95; Hippa and Vilkamaa (2004): 16; Mohrig et al. (2013): 265 [all as Xylosciara (Xylosciara) validinervis]. *Taxonomy*: Tuomikoski (1960): 92, 95; Menzel and Mohrig (2000): 569; Hippa and Vilkamaa (2004): 16; Mohrig et al. (2013): 265 [all as Xylosciara (Xylosciara) validinervis].

##### Locality.

• Finnmark; Tana, Tanafjorden, fjord Vestertana (= ‘Finmark, Vestertana’; = ‘Finnmark, Vestertana’).

##### Ecological note.

Habitats not specified. Phenology: Aug.

#### Zygoneura (Zygoneura) sciarina

Taxon classificationAnimaliaDipteraSciaridae

Meigen, 1830

A16333D2-3924-5158-8C92-113E45B35363

##### Literature.

*Faunistics*: Siebke (1877): 215; Lengersdorf (1926b): 4; Soot-Ryen (1942): 80 [all as *Zygoneura sciarina*]; Shin et al. (2014): 566 [as Zygoneura (Zygoneura) sciarina]. *Taxonomy*: Tuomikoski (1960): 156 [as *Zygoneura sciarina*]; Menzel and Mohrig (2000): 582 [as Zygoneura (Zygoneura) sciarina].

##### Localities.

• Norway; without further locality details (= ‘Norway’) • Oppland; Lunner, Brovoll N of Oslo (= ‘Brovold ad Christianiam’; = ‘Brovold, Oslo’; = ‘Brovold’).

##### Ecological note.

Habitats not specified. Phenology: Sep.

### Doubtful species

The names included in this category are to be understood as ‘unplaced species’ within the Sciaridae. A reliable interpretation of the species names and their unequivocal placement within the Sciaridae on the basis of Meigen’s original descriptions is not possible without revision of the types. Consequently, these may either be synonymous names, or the Norwegian specimens may have been misidentified by previous authors.

#### Sciara fuscipennis

Taxon classificationAnimaliaDipteraSciaridae

Meigen, 1818

D1A43783-93D4-590C-8C78-46335ACE1E3E

##### Literature.

*Faunistics*: Zetterstedt (1855): 4890 [as *Sciara fuscipennis*]; Soot-Ryen (1942): 78 [as *Neosciara fuscipennis*]. *Taxonomy*: Menzel and Mohrig (2000): 600 [as *Sciara fuscipennis*].

##### Locality.

• Norway; without further locality details (= ‘Norwegia’; = ‘Norge’).

##### Ecological note.

Habitats not specified. Phenology: without data.

#### Sciara longipes

Taxon classificationAnimaliaDipteraSciaridae

Meigen, 1818

F21178C6-5549-5C39-93C0-ADCF43044490

##### Literature.

*Faunistics*: Zetterstedt (1851): 3757; Siebke (1877): 214 [both as *Sciara longipes*]; Soot-Ryen (1942): 78 [as *Neosciara longipes*]. *Taxonomy*: Menzel and Mohrig (2000): 600 [as *Sciara longipes*].

##### Locality.

• Oslo; Oslo, Tøyen (= ‘in Tøien ad Christianiam’; = ‘ad Töien’; = ‘Tøyen, Oslo’).

##### Ecological note.

Habitats not specified. Phenology: Sep.

#### Sciara nigripes

Taxon classificationAnimaliaDipteraSciaridae

Meigen, 1830

CBD7F949-D79A-5955-9C1C-44964A8EFDA0

##### Literature.

*Faunistics*: Zetterstedt (1851): 3719 [as *Sciara nigripes*]; Siebke (1863): 176 [as *Sciara nigripes* Zetterstedt; recte Meigen]; Zetterstedt (1871): 3719; Siebke (1877): 210 [both as *Sciara nigripes*]; Soot-Ryen (1942): 79 [as *Neosciara nigripes*]. *Taxonomy*: Tuomikoski (1960): 52 [as *Sciara nigripes* in the discussion of *Corynoptera montana*]; Menzel and Mohrig (2000): 600 [as *Sciara nigripes*].

##### Localities.

• Nordland; Bodø, Bodø, Bjerkeng (= ‘Bjerkeng’) • Oppland; Lesja, Fogstuen on the Dovrefjell plateau (= ‘Fogstuen’; = ‘Fokstuen, Dovre’; = ‘in alpe Dovre ad Fokstuen’; = ‘in alpe Dovre’) • Oslo; Oslo, Tøyen (= ‘ad Christianiam in Tøien’; = ‘Tøyen, Oslo’) • Trøndelag; Verdal, Østre Nes at the Jamtlandsvegen [road no. 72] between Verdal and Lysthaugen (= ‘ad Oestre-Näs Værdaliæ’; = ‘ad Østre Næs Værdaliæ’; = ‘Østre Nes, Værdal’).

##### Ecological note.

Habitats not specified. Phenology: Jul.–Aug.

#### Sciara pulicaria

Taxon classificationAnimaliaDipteraSciaridae

Meigen, 1818

498CB18B-3F29-5DDB-A9D1-5CB0CA1ED41E

##### Literature.

*Faunistics*: Zetterstedt (1838): 827; Zetterstedt (1851): 3741; Zetterstedt (1855): 4890; Siebke (1866a): 385; Siebke (1877): 213; Lengersdorf (1926b): 9 [all as *Sciara pulicaria*]; Soot-Ryen (1942): 79 [as *Neosciara pulicaria*]. *Taxonomy*: Menzel and Mohrig (2000): 600 [as *Sciara pulicaria*].

##### Localities.

• Norway; without further locality details (= ‘Nord-Norwegen’) • Møre Og Romsdal; Rauma, between Veblungsnes and Romsdalshornet Mountain in the Romsdalsalpene SE of Åndalsnes (= ‘Romsdals Amt, mellem Veblungsnæsset og Romsdalshorn’) • Rauma, Veblungsnes at the Romsdalsfjorden SW of Åndalsnes (= ‘ad Veblungsnæs Romsdaliæ; = ‘Veblungsnes, Romsdal’) • Oslo; Oslo (= ‘ad Christianiam’) • Oslo, Bekkelaget (= ‘Bækkelgaet’; = ‘Bekkelaget’) • Oslo, Tøyen (= ‘circa Christianiam ... in Tøien’; = ‘Tøyen, Oslo’) • Troms; Berg/Lenvik/Tranøy/Torsken, Senja Island (= ‘Nordlandiæ Norwegieæ insula Senjen’; = Nordlandiæ, insula Senjen’; = ‘insula Senjen Nordlandiæ’; = ‘Senja’).

##### Ecological note.

Habitats not specified. Phenology: May–Aug.

### Checklist of Norwegian Sciaridae

***Bradysia* Winnertz, 1867**

*affinis* (Zetterstedt, 1838)

*alpicola* (Winnertz, 1867)

*angustipennis* Winnertz, 1867

*bicolor* (Meigen, 1818)

*brevispina* Tuomikoski, 1960

*confinis* (Winnertz, 1867)

*distincta* (Staeger, 1840)

*fenestralis* (Zetterstedt, 1838)

*flavipila* Tuomikoski, 1960

*forficulata* (Bezzi, 1914)

*fungicola* (Winnertz, 1867)

*giraudii* (Egger, 1862)

*hilariformis* Tuomikoski, 1960

*hilaris* (Winnertz, 1867)

*impatiens* (Johannsen, 1912)

*inusitata* (Tuomikoski, 1960)

*iridipennis* (Zetterstedt, 1838)

*lapponica* (Lengersdorf, 1926)

*longicubitalis* (Lengersdorf, 1924)

*nervosa* (Meigen, 1818)

*nitidicollis* (Meigen, 1818)

*opaca* (Winnertz, 1871)

*pallipes* (Fabricius, 1787)

*pauperata* (Winnertz, 1867)

*placida* (Winnertz, 1867)

*praecox* (Meigen, 1818)

*quercina* Menzel & Köhler, 2014

*rufescens* (Zetterstedt, 1852)

*sordida* (Zetterstedt, 1838)

*strenua* (Winnertz, 1867)

*strigata* (Staeger, 1840)

*tilicola* (Loew, 1850)

*trivittata* (Staeger, 1840)

*vernalis* (Zetterstedt, 1851)

***Bradysiopsis* Tuomikoski, 1960**

*vittigera* (Zetterstedt, 1851)

***Camptochaeta* Hippa & Vilkamaa, 1994**

*bournei* (Shaw, 1941)

*camptochaeta* (Tuomikoski, 1960)

*consimilis* (Holmgren, 1869)

*delicata* (Lengersdorf, 1935)

*fallax* Hippa & Vilkamaa, 1994

*hirtula* (Lengersdorf, 1934)

*mimica* Hippa & Vilkamaa, 1994

*truncata* Vilkamaa & Mohrig, 2013

*xystica* Hippa & Vilkamaa, 1994

***Chaetosciara* Frey, 1942**

*estlandica* (Lengersdorf, 1929)

***Claustropyga* Hippa, Vilkamaa & Mohrig, 2003**

*brevichaeta* (Mohrig & Antonova, 1978)

*refrigerata* (Lengersdorf, 1930)

***Corynoptera* Winnertz, 1867**

*boletiphaga* (Lengersdorf, 1940)

*brachypennis* (Lengersdorf, 1926)

*defecta* (Frey, 1948)

*fatigans* (Johannsen, 1912)

*flavicauda* (Zetterstedt, 1855)

*forcipata* (Winnertz, 1867)

*hypopygialis* (Lengersdorf, 1926)

*irmgardis* (Lengersdorf, 1930)

*membranigera* (Kieffer, 1903)

*minima* (Meigen, 1818)

*montana* (Winnertz, 1869)

*penna* (Pettey, 1918)

*roederi* (Lengersdorf, 1931)

*saetistyla* Mohrig & Krivosheina, 1985

*sphenoptera* Tuomikoski, 1960

*spoeckeri* (Lengersdorf, 1930)

*subtilis* (Lengersdorf, 1929)

*subvariegata* Rudzinski, 1992

*trepida* (Winnertz, 1867)

*waltraudis* Mohrig & Mamaev, 1987

***Cratyna* Winnertz, 1867**

SGCratyna Winnertz, 1867 s. str.

*ambigua* (Lengersdorf, 1934)

*atra* Winnertz, 1867

*hirticornis* (Meigen, 1818)

*longipennis* (Lengersdorf, 1931)

*uliginosa* (Lengersdorf, 1929)

*uliginosoides* Heller, Köhler & Menzel, 2016

SGSpathobdella Frey, 1948

*colei* (Freeman, 1990)

*falcata* (Tuomikoski, 1960)

*longispina* (Pettey, 1918)

*nobilis* (Winnertz, 1867)

*perplexa* (Winnertz, 1867)

***Ctenosciara* Tuomikoski, 1960**

*hyalipennis* (Meigen, 1804)

*lutea* (Meigen, 1804)

***Dichopygina* Vilkamaa, Hippa & Komarova, 2004**

*aculeata* Vilkamaa, Hippa & Komarova, 2004

*bernhardi* Vilkamaa, Hippa & Komarova, 2004

*nigrohalteralis* (Frey, 1948)

*ramosa* Vilkamaa, Hippa & Komarova, 2004

***Dolichosciara* Tuomikoski, 1960**

*flavipes* (Meigen, 1804)

***Epidapus* Haliday, 1851**

SGEpidapus Haliday, 1851 s. str.

*alnicola* (Tuomikoski, 1957)

*gracilis* (Walker, 1848)

***Hemineurina* Frey, 1942**

*abbrevinervis* (Holmgren, 1869)

*conspicua* (Winnertz, 1867)

*inflata* (Winnertz, 1867)

*modesta* (Staeger, 1840)

*postconspicua* (Mohrig, 1985)

*venosa* (Staeger, 1840)

***Leptosciarella* Tuomikoski, 1960**

SGHirtipennia Mohrig & Menzel, 1997

*hirtipennis* (Zetterstedt, 1838)

SGLeptosciarella Tuomikoski, 1960 s. str.

*fuscipalpa* (Mohrig & Mamaev, 1979)

*hispida* (Winnertz, 1867)

*nudinervis* (Tuomikoski, 1960)

*pilosa* (Staeger, 1840)

*scutellata* (Staeger, 1840)

*trochanterata* (Zetterstedt, 1851)

*truncata* (Tuomikoski, 1960)

***Lycoriella* Frey, 1942**

*brevipila* Tuomikoski, 1960

*ingenua* (Dufour, 1839)

*latilobata* Menzel & Mohrig, 2000

*parva* (Holmgren, 1869)

*piristylata* Vilkamaa, Hippa & Heller, 2013

*sativae* (Johannsen, 1912)

***Pseudolycoriella* Menzel & Mohrig, 1998**

*paludum* (Frey, 1948)

***Scatopsciara* Edwards, 1927**

SGScatopsciara Edwards, 1927 s. str.

*atomaria* (Zetterstedt, 1851)

*brevicornis* (Zetterstedt, 1851)

*calamophila* Frey, 1948

*fluviatilis* (Lengersdorf, 1940)

*multispina* (Bukowski & Lengersdorf, 1936)

*nana* (Winnertz, 1871)

*neglecta* Menzel & Mohrig, 1998

*pusilla* (Meigen, 1818)

*vitripennis* (Meigen, 1818)

***Schwenckfeldina* Frey, 1942**

*carbonaria* (Meigen, 1830)

*tridentata* (Rübsaamen, 1898)

***Sciara* Meigen, 1803**

*flavimana* Zetterstedt, 1851

*hemerobioides* (Scopoli, 1763)

*humeralis* Zetterstedt, 1851

*ruficauda* Meigen, 1818

***Trichocoelina* Vilkamaa & Menzel, 2019**

*brevicubitalis* (Lengersdorf, 1926)

*cochleata* (Rübsaamen, 1898)

*ithyspina* Vilkamaa & Menzel, 2019

*jukkai* Vilkamaa & Menzel, 2019

*obesula* Vilkamaa & Menzel, 2019

*oricillifera* Vilkamaa & Menzel, 2019

*semisphaera* Vilkamaa & Menzel, 2019

*vitticollis* (Holmgren, 1883)

***Trichosia* Winnertz, 1867**

SGMouffetina Frey, 1942

*expolita* (Coquillett, 1900)

SGTrichosia Winnertz, 1867 s. str.

*caudata* (Walker, 1848)

*confusa* Menzel & Mohrig, 1997

*edwardsi* (Lengersdorf, 1930)

*flavicoxa* Tuomikoski, 1960

*lengersdorfi* Heller, Köhler & Menzel, 2016

*splendens* Winnertz, 1867

***Xylosciara* Tuomikoski, 1957**

SGXylosciara Tuomikoski, 1957 s. str.

*heptacantha* Tuomikoski, 1957

*spinata* (Pettey, 1918)

*trimera* Tuomikoski, 1960

*validinervis* Tuomikoski, 1960

***Zygoneura* Meigen, 1830**

SGZygoneura Meigen, 1830 s. str.

*sciarina* Meigen, 1830

**Doubtful species**

*fuscipennis* Meigen, 1818 [*Sciara*]

*longipes* Meigen, 1818 [*Sciara*]

*nigripes* Meigen, 1830 [*Sciara*]

*pulicaria* Meigen, 1818 [*Sciara*]

## Discussion

In this literature review we document the knowledge on the Sciaridae of Norway accumulated up to 31 December 2019, which was basically the status quo before we started our nationwide taxonomic inventory funded by the NBIC. Nonetheless, data compiled here are the result of a meticulous study of the literature in the past six years, and thus a direct outcome of our NTI projects.

**Figure 5.**
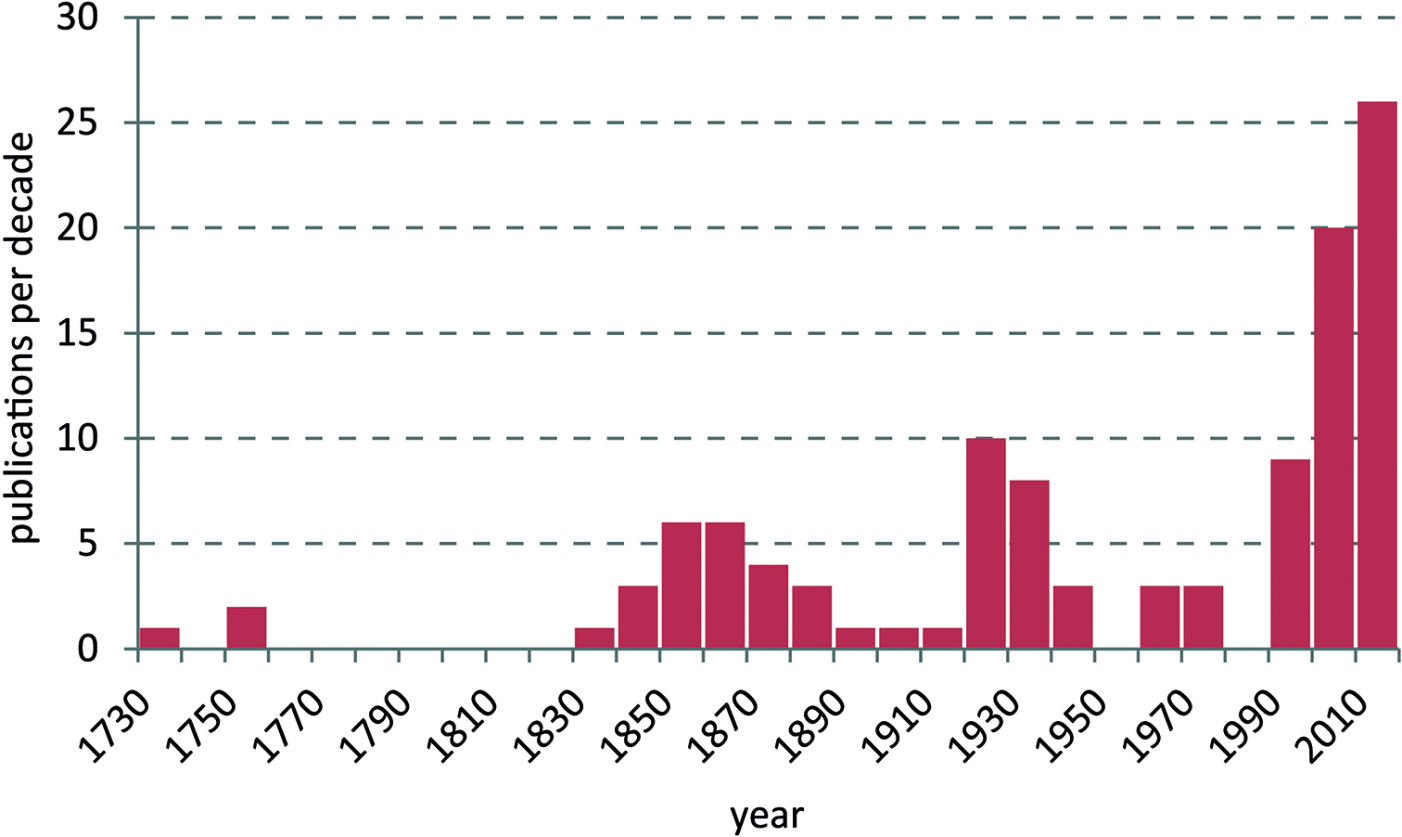
Number of publications on Norwegian sciarid fauna per decade between 1730 and 2020.

**Figure 6.**
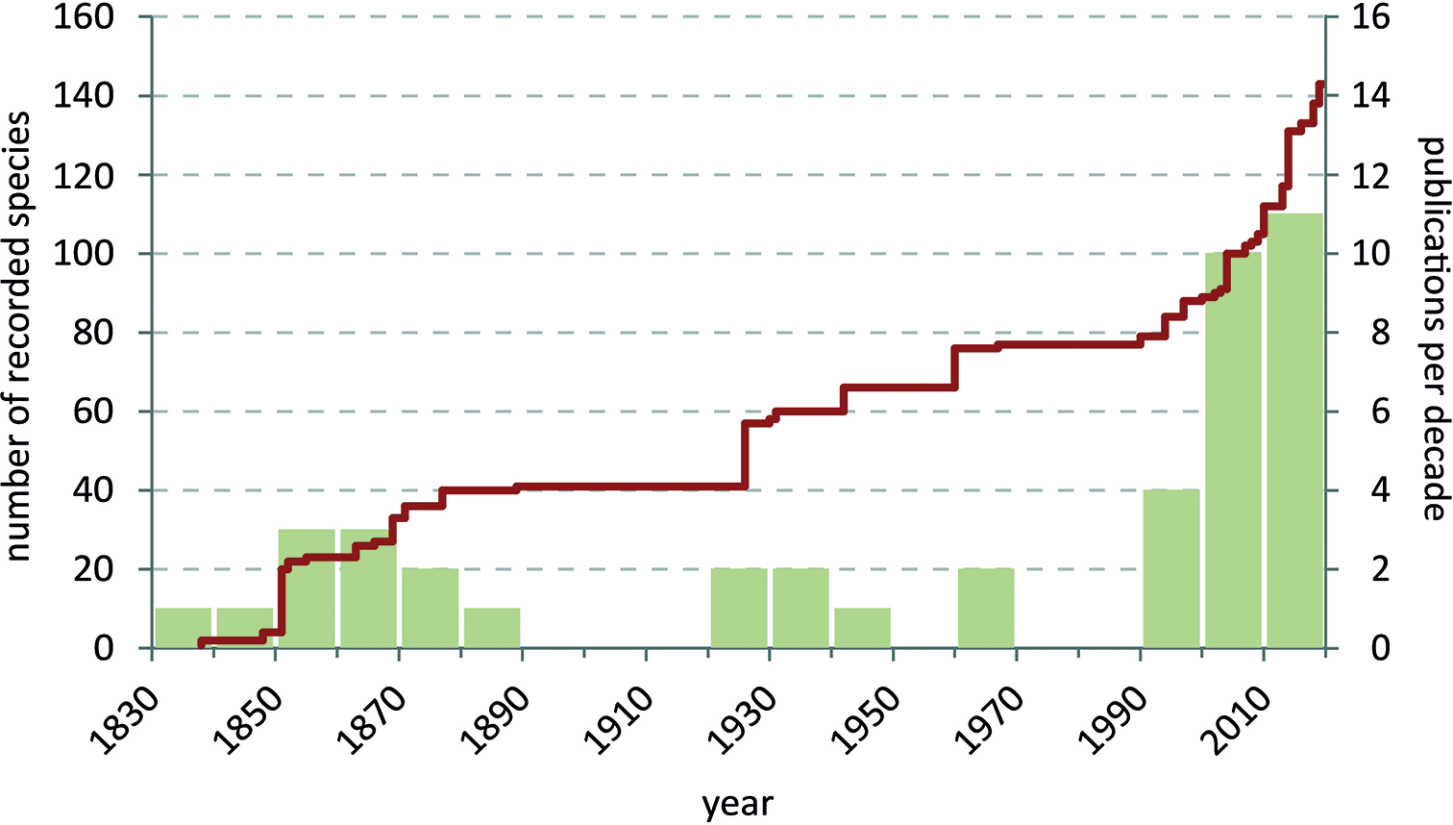
Cumulative increase of the number of Norwegian species records of Sciaridae (red line) and the number of corresponding publications (bars) containing first records per decade until 31 December 2019.

**History of data collection.** The first mention of black fungus gnats in Norway was by Ramus (1735). In our literature study we evaluated 111 literature sources published during a period of 285 years (Fig. 5). Of these, 43 papers contain first records of species identified between 1838 and 2019 (Fig. 6). Most publications reported the occurrence of ‘army worms’ until the middle of the 19^th^ century and it was only with Zetterstedt (1838) that faunistic investigations began to be based on detailed Norwegian data at the species level. Of the 147 species now registered, the taxonomic status of four recorded by Zetterstedt (1838, 1851, 1855) is still unclear and these are treated here as doubtful species.

Not surprisingly, knowledge about the composition of the Norwegian sciarid fauna did not increase continuously. Roughly three different time periods of taxonomic work can be distinguished (Fig. 6). The first period began with the work of Johann Wilhelm Zetterstedt (1785–1874), who described the first two Norwegian species in 1838. Later he published four additional books containing Norwegian records (Zetterstedt 1851, 1852, 1855, 1871). Other famous entomologists such as Francis Walker (1809–1874), Johan Heinrich Spalckhawer Siebke (1816–1875), August Emil Holmgren (1829–1888), and Wilhelm Maribo Schøyen (1844–1918) also contributed to an inventory of the Norwegian fauna. After 51 years, 41 sciarid species were recorded, representing 28% of the currently known species inventory. After an intermission of over 35 years, the second period began in 1926. Between 1926 and 1931 Franz Lengersdorf (1880–1965) added 19 new records to the faunistic inventory. Shortly thereafter, in the timescale of taxonomic and faunistic studies, Tron Soot-Ryen (1896–1986) and the founder of modern sciarid taxonomy Risto Kalevi Tuomikoski (1911–1989) recorded a further 17 species. Thus, in the second period, 36 species were recorded for the first time in Norway, representing a quarter of the known fauna. In the early 1990s, the number of publications and consequently the number of recorded species rose steeply. The increase was almost 86%, from 77 before 1990 to the current 143. The majority of these new records were provided by the dipterists Heikki Hippa, Frank Menzel, and Pekka Vilkamaa.

For the closely related family Mycetophilidae (fungus gnats), Gammelmo and Søli (2006) described a similar curve of knowledge increase. Here, also, the beginning of the recording of the Norwegian fauna goes back to the middle of the 19^th^ century. Through several fundamental works by J.W. Zetterstedt and J.H.S. Siebke, Siebke (1877) was already able to list an inventory of 53 Norwegian species. After this period the Mycetophilidae received only little attention until a few publications appeared in the 1970s. This was followed in 1994 by a steep increase in faunistic knowledge, leading to more than 600 fungus gnat species having been recorded from Norway to date (see Gammelmo and Søli (2006), and subsequent papers). Records of approximately 200 additional species discovered in recent studies will soon be published (J. Kjærandsen, pers. comm.).

**Diversity in Northern Europe.** It is obvious that the 143 species summarised here are only a part of the extant sciarid fauna in Norway. We know of numerous additional species that will be dealt with in subsequent publications, including many new to science. We anticipate that the number of species in Norway is at least similar to that in Finland (370) (Vilkamaa 2014, Heller et al. 2015, Salmela et al. 2015, Hippa and Vilkamaa 2016, Vilkamaa and Menzel 2019) and Sweden (299) (Heller et al. 2009, 2015, 2016; Heller and Menzel 2013; Vilkamaa et al. 2013a–c; Vilkamaa and Menzel 2019), although the inventory for Sweden in particular is highly incomplete (Menzel et al. 2020). Even so, the results of our projects should not be regarded as exhaustive; there are still large areas, including very promising habitats, in which sciarids remain poorly sampled or have not been collected at all. Also, as the senior author’s experience with the German fauna (more than 650 species) has shown, the high proportion of rare, or rarely collected, species makes it impossible to achieve a comprehensive inventory during a study period of only five years. Due to the diversity of landscape structures, climate conditions, and habitats, the number of sciarid species in Norway, including the Arctic islands, must be 20% higher than that in Sweden and Finland. Consequently, knowledge on the black fungus gnats in Norway summarised here is still incomplete and represents only 30% of the estimated inventory of ca. 450–500 species. The numbers mentioned above are an indication that we are still far from having a complete knowledge of sciarid diversity in Scandinavia, and that extensive research will be needed in the future.

**Figure 7.**
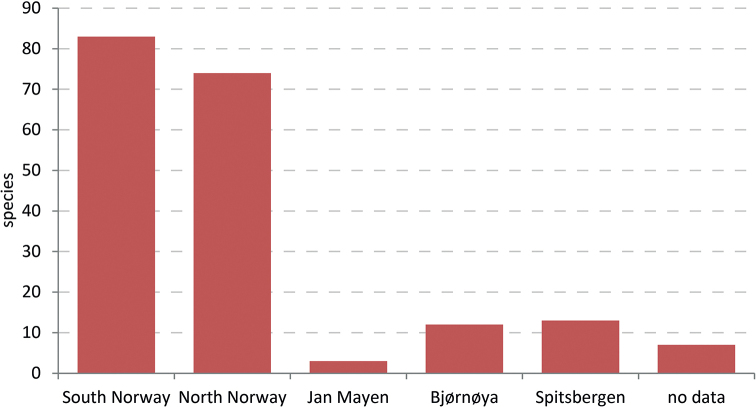
Distribution of sciarid species in Norway based on published records until 31 December 2019. ‘North Norway’ includes the counties Nordland, Troms and Finnmark, while the remaining counties of the Norwegian mainland are grouped as ‘South Norway’.

**Distribution and phenology in Norway.** A rough summary of recorded species by mainland counties south and north of the Arctic Circle, including the offshore islands (Fig. 7) shows that a majority of 83 species were found in southern Norway while the northern mainland supports 74 species. The known species inventory of the Arctic islands ranges from three (Jan Mayen) to 13 (Spitsbergen). Our literature survey shows that some species are very common and widely distributed on the Norwegian mainland (e.g., *Bradysia nitidicollis*, *B. rufescens*, *Ctenosciara hyalipennis*, *Lycoriella ingenua*, *Scatopsciara atomaria*, *Sc. vitripennis*), similar to the situation in other European countries. Some species not only inhabit the entire mainland, but also reach the arctic islands (e.g., *Bradysia nervosa*, *B. praecox*). In addition, there are also species with a relatively large number of records, which are apparently adapted to a harsh climate with a short vegetation-growth period. These species (e.g., *Camptochaeta consimilis*, *Cam. delicata*, *Schwenckfeldina tridentata*, *Trichocoelina cochleata* and *Trichoc. vitticollis*) were only found in the far north (Troms, Finnmark) and/or on the Arctic islands of Jan Mayen, Bjørnøya and Spitsbergen. On the other hand, several species seem to occur only in southern Norway (e.g., *Cratyna uliginosoides*, *Sciara hemerobioides*, *Trichosia lengersdorfi*). The areas south of the Arctic Circle in particular have not been sufficiently investigated. At least 350 sciarid species are expected in southern Norway including the high mountains. By contrast, the number of species on the Arctic islands will be probably increase only slightly (up to 20).

The very species-rich genera *Bradysia* Winnertz, *Corynoptera* Winnertz and *Scatopsciara* Edwards are still largely underrepresented in the papers published so far (see checklist). Many Holarctic species of the genera *Claustropyga* Hippa, Vilkamaa & Mohrig, *Hemineurina* Frey, and *Lycoriella* Frey are also still missing. Only one or two species of *Epidapus* Haliday, *Dolichosciara* Tuomikoski and *Pseudolycoriella* Menzel and Mohrig have been reported from Norway so far. The genera *Cosmosciara* Frey, *Hyperlasion* Schmitz, *Phytosciara* Frey, *Prosciara* Frey, *Pnyxia* Johannsen, *Pnyxiopsis* Tuomikoski, *Scythropochroa* Enderlein and *Stenacanthella* Vilkamaa and Menzel are not known yet from Norway. They were recorded from many countries in central and northern Europe, mostly with few species, and may also be present in Norway.

**Figure 8.**
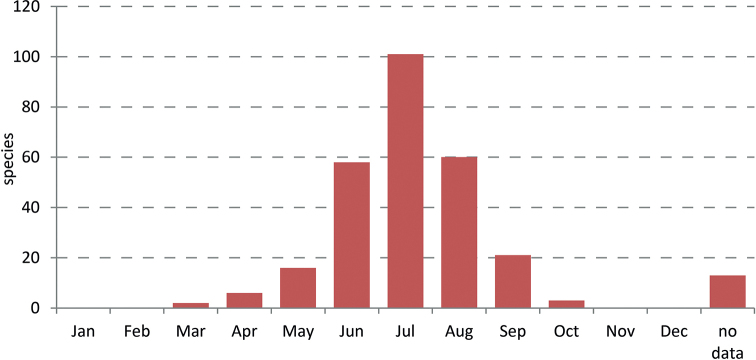
Phenology of sciarid species in Norway based on the flight times of adults summarised from the published records until 31 December 2019.

According to all literature sources, sciarids were found from March to October with a clear peak in July (Fig. 8). However, this is far from providing a realistic picture of the phenology. Together with the higher ‘accumulation of species’ in June and August, it reflects the preferred collecting period of entomologists in the summer rather than the actual phenology of Sciaridae. The sciarid records considered here are mostly from by-catches, whereas targeted long-term studies carried out with standardised trapping methods over several years in the same habitat type in Norway have not yet been undertaken.

Thiede (1977) and Feldmann (1992), for example, studied the emergence times and activity patterns of sciarids over a two to three year period in selected beech, spruce and pine forests in Germany. They found that sciarid phenology can vary significantly between studied forest ecosystems based on the species identified. Under temperate climatic conditions (e.g., in Central Europe) adults usually have two activity phases: mid-March to early June and early August to late September, with two peaks in April and September. Depending on precipitation and temperature, the first peak may shift to March or May and the second peak to June/July or October/November. Numerous ecological studies have shown that some species complete two generations per year in Central Europe, in spring and late summer or autumn. Other species are univoltine, with only one generation in spring, summer or late summer (Thiede 1977).

Unfortunately, data on Norwegian sciarids are still too sparse for a solid evaluation. Some common species are present from spring to autumn, similar to those in Central Europe (e.g., *Bradysia nitidicollis*, *Cratyna uliginosa*, *Cr. uliginosoides*, *Lycoriella ingenua*, *Scatopsciara atomaria*, *Scatopsciara vitripennis*, *Trichosia lengersdorfi*). In southern Norway some species could be bivoltine (e.g., *Bradysia iridipennis*, *Trichosia caudata*). It is to be expected for Norway that the phenologies of species adapted to temperate habitats will differ clearly from those of subpolar sites. The period of adult activity probably shortens significantly with increasing northern latitude, shifting to the summer months of June to August due to the short vegetation-growth period in the far north and Arctic islands (e.g. *Camptochaeta consimilis*, *C. delicata*, *Trichocoelina vitticollis*).

**Outlook.** The Sciaridae is still one of the most poorly studied families of Diptera in Norway, especially in the interior of the country, which is mostly unexplored. The life history of most Norwegian sciarid species (including immature stages and life cycles) are largely unknown. In addition, at present only little information exists on habitat preferences of the northern European species, especially those with a subarctic and arctic distribution. As a consequence, the family was not included in the new Red List for Norway (Gammelmo et al. 2015). Knowledge on Sciaridae at the species level is important for understanding the complexity of terrestrial ecosystems, in particular woodland decomposition processes. The first step in establishing such knowledge must be to determine which species occur in Norway and in which habitats they thrive.

The above-mentioned NTI projects (including the present study) aimed to survey sciarids that are found in the wide array of natural habitats in Norway and in the ‘Natur i Norge’ (NiN) system. Another objective is to provide the Norwegian scientific community with tools for identifying Norwegian sciarids, including identification keys, reference collections and genetic resources. Both projects will also contribute to global biodiversity initiatives by providing data on species occurrence, genetic diversity and geographic distribution. Knowledge on the sciarid fauna in Norway is thus expected to increase considerably in the next few years. Continuous collecting efforts and taxonomic studies will provide a solid new base of knowledge on Sciaridae in Norway. Finally, we hope that the present study will contribute to a better understanding of an interesting insect group and close existing gaps of knowledge in biodiversity research, especially on the sciarid fauna of Scandinavia.
